# Palladium-catalyzed difluoroalkylative carbonylation of styrenes toward difluoropentanedioates†

**DOI:** 10.1039/d2sc02665a

**Published:** 2022-08-03

**Authors:** Zhi-Peng Bao, Youcan Zhang, Xiao-Feng Wu

**Affiliations:** Dalian National Laboratory for Clean Energy, Dalian Institute of Chemical Physics, Chinese Academy of Sciences 116023 Dalian Liaoning China xwu2020@dicp.ac.cn; Leibniz-Institut für Katalyse e.V. Albert-Einstein-Straße 29a 1 8059 Rostock Germany Xiao-Feng.Wu@catalysis.de

## Abstract

The introduction of fluorine atoms into organic molecules is an attractive but challenging topic. In this work, an interesting palladium-catalyzed difluoroalkylative carbonylation of aryl olefins has been developed. A wide range of aryl olefins were transformed into the corresponding difluoropentanedioate compounds with good functional-group tolerance and excellent regioselectivity. Inexpensive ethyl bromodifluoroacetate acts both as a difluoroalkyl precursor and a nucleophile here. Additionally, a scale–up reaction was also performed successfully, and further transformations of the obtained product were shown as well.

## Abstract 2

An interesting palladium-catalyzed difluoroalkylative carbonylation of aryl olefins has been developed. A wide range of aryl olefins were transformed into the corresponding difluoropentanedioate compounds with good functional-group tolerance and excellent regioselectivity.

## Graphical abstract

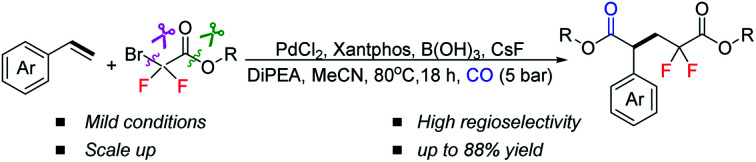

## Introduction

**Scheme 1:**
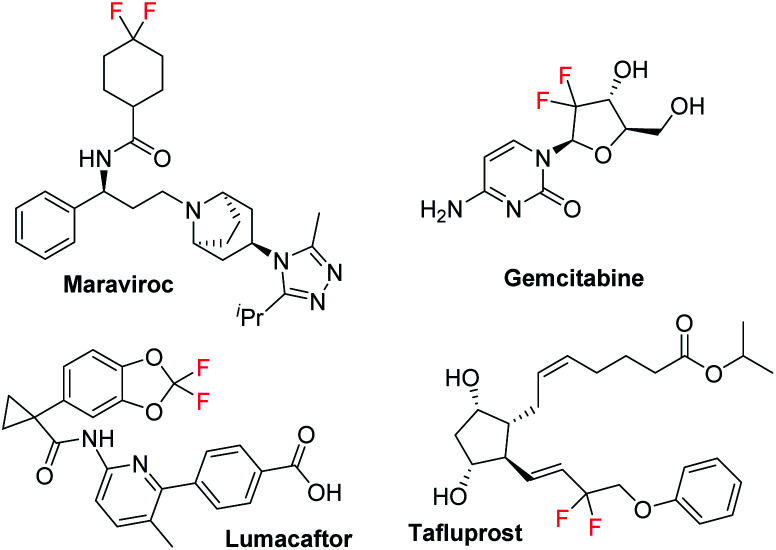
Examples of drug molecules containing a difluoromethyl moiety.

Organic fluorides play an important role in organic synthesis, medicinal chemistry, and materials science due to their special physical and chemical properties.^1^ The introduction of a fluorine atom into an organic molecule can often change the biological activity and physical properties of the compound. Among the fluorine containing moieties, the difluoromethylene group has good metabolic stability, and its electron-withdrawing character can affect the electronic properties, chemical properties, and biological reactivity of the adjacent functional groups among the fluorine-containing compounds, thus it exists in diverse drug molecules.^2^ For example, Maraviroc is a CCR5 co-receptor antagonist used for treating CCR5-tropic HIV-1 infection together with other antiretroviral medications.^3^ Gemcitabine, a nucleoside metabolic inhibitor, is used as an adjunct therapy in the treatment of certain types of ovarian cancer, non-small cell lung carcinoma, metastatic breast cancer, and as a single agent for pancreatic cancer.^4^ Lumacaftor is a protein chaperone, used for the treatment of cystic fibrosis in patients who are homozygous for the F508del mutation in the CFTR gene by combining with ivacaftor.^5^ Tafluprost, an ophthalmic prostaglandin analogue, has been used to lower intraocular pressure in patients with ocular hypertension or open-angle glaucoma (Scheme 1).^6^

**Scheme 2:**
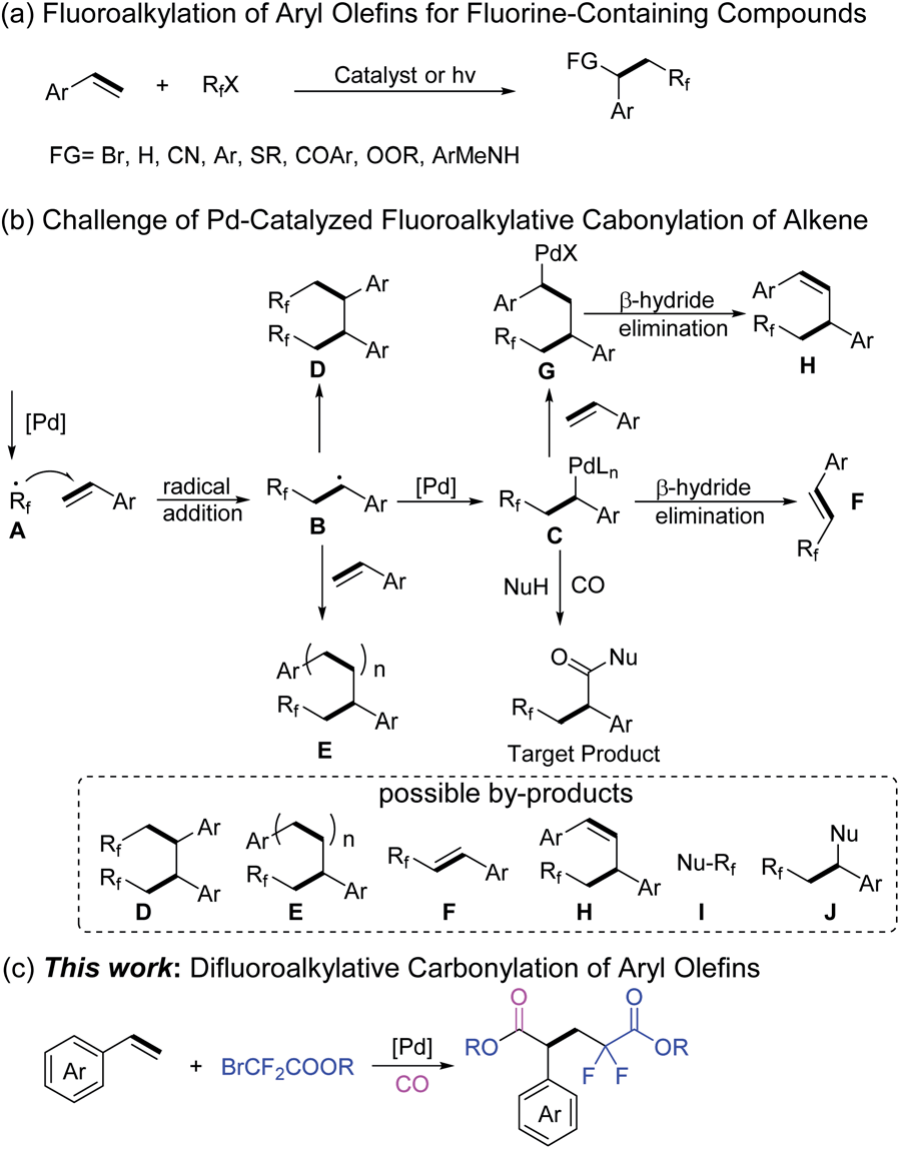
Palladium-catalyzed fluoro functionalization of aryl olefins.

Due to the double bond of aryl alkenes conjugated with an aromatic ring, they are quite activated and will usually lead to selectivity and reactivity issues in organic transformations. On the other hand, it represents an attractive route to construct fluorinated compounds by using aryl olefins and commercially available fluoroalkyl halides as the starting materials.^7^ Various transition-metal-catalyzed-,^8^ photoinduced-^9^ or *N*-heterocyclic carbene (NHC) catalyzed-^10^ fluoroalkylation reactions of aryl alkenes with fluoroalkyl halides have been developed for the preparation of difunctional saturated fluorine-containing compounds (Scheme 2a).

Transition metal-catalyzed carbonylation is an effective strategy for preparing various functionalized carbonyl-containing compounds.^11^ In recent years, more and more procedures for fluoroalkylative carbonylations of olefins have been developed for the construction of fluorine-containing carbonylated compounds. For example, Liu's group reported a novel cooperative strategy based on palladium-catalyzed and iodine(iii)-mediated β-fluorocarboxylation of alkenes;^12^ palladium-catalyzed multi-component perfluoroalkylative carbonylation for the synthesis of β-perfluoroalkyl esters and amides was also realized;^13^ and a copper-catalyzed 1,2-trifluoromethylation carbonylation of unactivated alkenes to get β-trifluoromethylated aliphatic carboxylic acid derivatives has been reported recently.^14^ However, most of them are based on unactivated aliphatic olefins, and the transformation of aryl olefins in the fluoroalkylative carbonylation reaction to give the corresponding ester product is still not reported.

Under all the above discussed backgrounds, we became interested in developing a new fluoroalkylative carbonylation procedure for aryl olefins to construct fluorine-containing carbonylated compounds. However, this strategy faces several challenges. As depicted in Scheme 2b, palladium-mediated single-electron reduction of fluoroalkyl halide forms radical A, which subsequently adds to the aryl olefins to generate a new benzylic carbon radical B. The intermediate B recombines with palladium to form a new active benzylic intermediate C. Meanwhile, B may dimerize to form D, which may even continue to polymerize with other aryl olefins to form product E. Intermediate C can give product F after β-hydride elimination.^7*a*^ Intermediate C can also continue to add to another olefin to form G, and then β-hydride elimination takes place to give product H.^15^ Theoretically, fluoroalkyl halide can also react directly with a nucleophile to give compound I, and C may be quenched by a nucleophile to form J. Hence, a selective and efficient procedure for fluoroalkylative carbonylation of aryl olefins to construct fluorine-containing carbonylated compounds is a challenging topic.

After systematic optimizations, herein, we developed an efficient palladium-catalyzed difluoroalkylative carbonylation reaction for aryl olefins. Ethyl bromodifluoroacetate acts both as a difluoroalkyl precursor and a nucleophile in this system. A wide range of aryl olefins were transformed into the corresponding difluoropentanedioate compounds in good yields with broad functional group tolerance and excellent regioselectivity (Scheme 2c).

## Results and discussion

Initially, styrene 1a and cheap ethyl bromodifluoroacetate 2a were chosen as the model substrates to evaluate the feasibility of this difluoroalkylation carbonylation reaction. To our delight, with DiPEA as the base and assisted by B(OH)_3_ in dioxane at 80 °C, the desired product 3aa was obtained in 57% yield in the presence of PdCl_2_ and using Xantphos as the ligand (Table 1, entry 1). Subsequently, various bases were studied, and a lower yield was observed with Na_2_CO_3_ (Table 1, entry 2). The desired product 3aa was not detected when using NaO^*t*^Bu as the base (Table 1, entry 3). We then studied the effect of palladium pre-catalysts, but unfortunately, reduced yields were obtained when Pd(OAc)_2_, Pd(PPh_3_)_4_, or Pd(TFA)_2_ were tested (Table 1, entries 4–6). Further screening showed that 0.4 mmol of boric acid was proven to be the best for the target transformation (Table 1, entry 7 *vs.* 6). The yield of 3aa dropped to 17% in the absence of boric acid (Table 1, entry 8). And the yield of 3aa increased to 66% when employing MeCN as the solvent (Table 1, entry 9). Then we studied the effect of ligands, but reduced yields were obtained with the tested ligands (Table 1, entries 10–13). Interestingly, the yield of 3aa was almost not changed when we decreased the pressure of CO to 5 bar (Table 1, entry 14). To our satisfaction, 3aa was obtained in 81% yield when CsF was used as an additive (Table 1, entry 15).

**Table 1:** Optimization of reaction conditions^a^

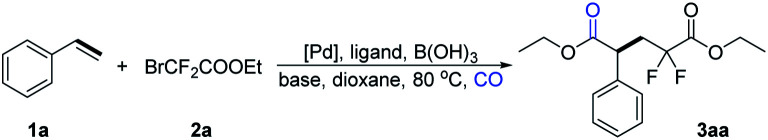					
Entry	[Pd]	Ligand	Base	B(OH)_3_ (mmol)	Yield [%]^b^
1	PdCl_2_	Xantphos	DiPEA	0.2	57
2	PdCl_2_	Xantphos	Na_2_CO_3_	0.2	19
3	PdCl_2_	Xantphos	NaO^*t*^Bu	0.2	N.D.
4	Pd(OAc)_2_	Xantphos	DiPEA	0.2	50
5	Pd(PPh_3_)_4_	Xantphos	DiPEA	0.2	46
6	Pd(TFA)_2_	Xantphos	DiPEA	0.2	46
7	PdCl_2_	Xantphos	DiPEA	0.4	60
8	PdCl_2_	Xantphos	DiPEA	0	17
9^c^	PdCl_2_	Xantphos	DiPEA	0.4	66
10^c^	PdCl_2_	PPh_3_	DiPEA	0.4	50
11^c^	PdCl_2_	DPEphos	DiPEA	0.4	18
12^c^	PdCl_2_	Nixantphos	DiPEA	0.4	46
13^c^	PdCl_2_	DPPP	DiPEA	0.4	Trace
14^c^^,^^d^	PdCl_2_	Xantphos	DiPEA	0.4	67
15^c^^,^^d^^,^^e^	PdCl_2_	Xantphos	DiPEA	0.4	81 (78)^f^

a Reaction conditions: 1a (0.3 mmol), 2a (0.9 mmol), [Pd] (10 mol%), monodentate ligand (20 mol%) or bidentate ligand (10 mol%), B(OH)_3_, base (1.2 mmol) in dioxane (1.5 mL) at 80 °C for 18 h under CO (10 bar).

b Yields were determined by GC-FID analysis using *n*-hexadecane as an internal standard.

c MeCN as solvent.

d CO (5 bar).

e CsF (0.3 mmol) as an additive.

f Yield of the isolated product.

**Scheme 3:**
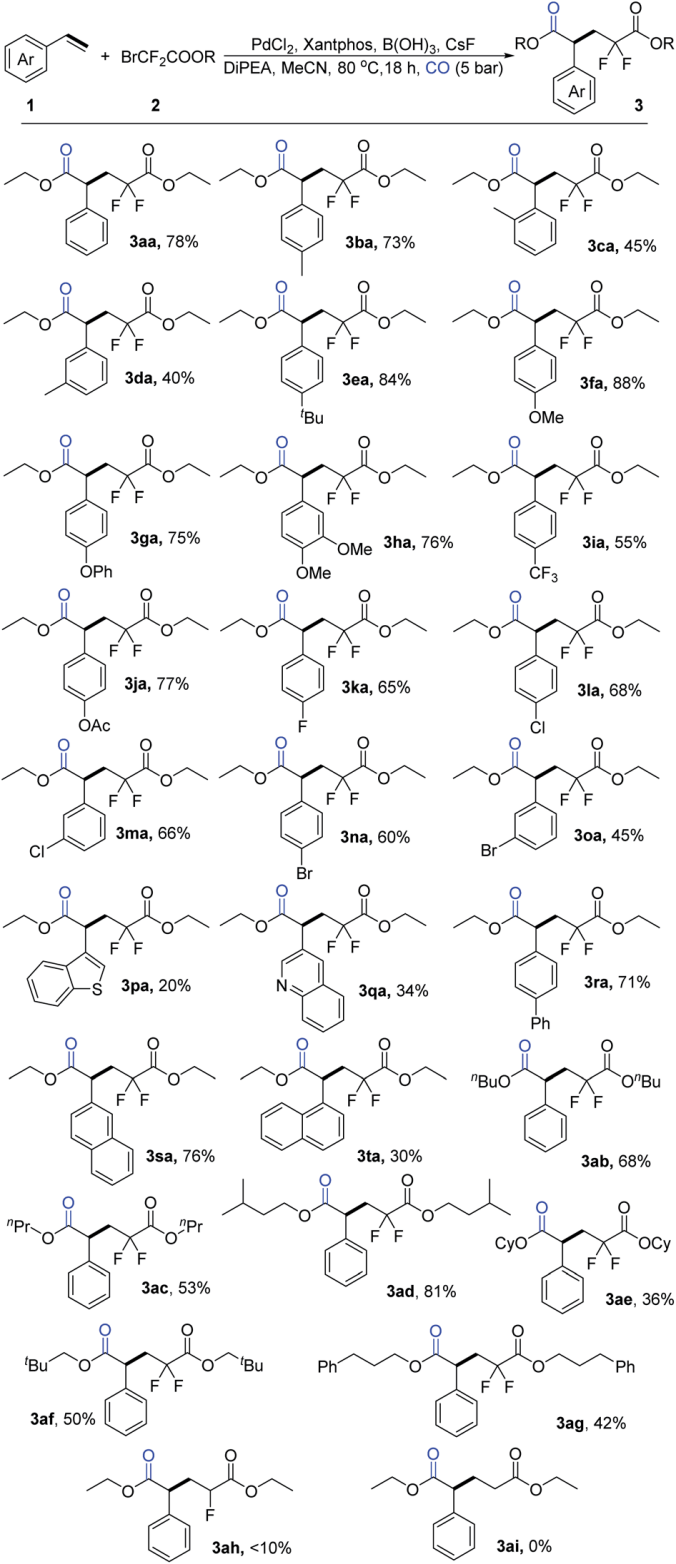
Scope of aryl olefins and bromodifluoroacetates. [a] Reaction conditions: 1 (0.3 mmol), 2a (0.9 mmol), PdCl_2_ (10 mol%), Xantphos (10 mol%), B(OH)_3_ (0.4 mmol), DiPEA (1.2 mmol), CsF (0.3 mmol), MeCN (1.5 mL), CO (5 bar), 80 °C, 18 h, isolated yields.

With the best reaction conditions in hand, we conducted our investigation into substrate scope, and a variety of aryl olefins were tested (Scheme 3). Aryl olefins with electron-donating groups, such as methyl, *tert*-butyl, methoxy, benzeneoxy, and 3, 4-dimethoxy groups were tolerated well to give the desired difluoropentanedioate products in moderate to high isolated yields (3ba-3ha), Notably, the yield can reach up to 88% when the olefin bears a methoxy group at the *para*-position. It should be mentioned that *ortho*-substituted styrene provided the corresponding product in lower yield compared with *para*-substituted styrene probably due to the steric hindrance (3ca*vs*3ba). Aryl olefins bearing electron-withdrawing groups, such as trifluoromethyl, acetate, can afford the target products in moderate to good yields as well (3ia-3ja). For those substrates with halogen groups, including fluoro, chloro, and bromo substituents, the desired products were isolated in good yields (3ka-3oa). To our delight, 3-vinylbenzo thiophene and 3-vinylquinoline were also tolerable under our standard conditions (3pa-3qa). Moreover, substrates with 1-biphenyl and 2-naphthalene moieties could also work well to give the corresponding products in good yields (3ra-3sa). 1-Vinylnaphthalene gave the corresponding product in lower yield compared with 2-vinylnaphthalene probably because of the steric hindrance (3ta*vs.*3sa). Moreover, six additional examples of bromodifluoroacetates were examined and the desired products 3ab-3ag were all isolated in moderate to good yields. However, when ethyl 2-bromo-2-fluoroacetate and ethyl 2-bromoacetate were tested, very low or no yield of the desired product could be detected (3ah, 3ai).

**Scheme 4:**
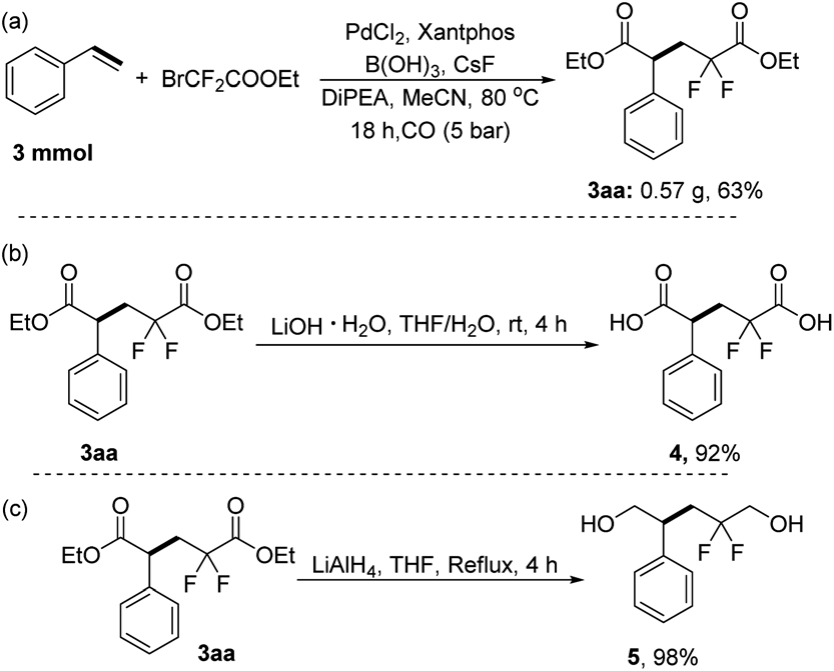
A scale-up reaction and transformations of the product 3aa.

To demonstrate the scalability and utility of this method, we conducted a scale-up reaction and further transformations of the obtained product 3aa. The desired diethyl 2, 2-difluoro-4-phenylpentanedioate 3aa can still be obtained in 63% yield when we expanded the reaction by 10 times (Scheme 4a). Then 2, 2-difluoro-4-phenylpentanedioic acid was obtained with 92% yield by alkaline hydrolysis of the product 3aa in THF/H_2_O with LiOH as the base (Scheme 4b). Subsequently, 98% yield of 2,2-difluoro-4-phenylpentane-1,5-diol was achieved from the product 3aa by using lithium aluminum hydride as the reductant (Scheme 4c).

**Scheme 5:**
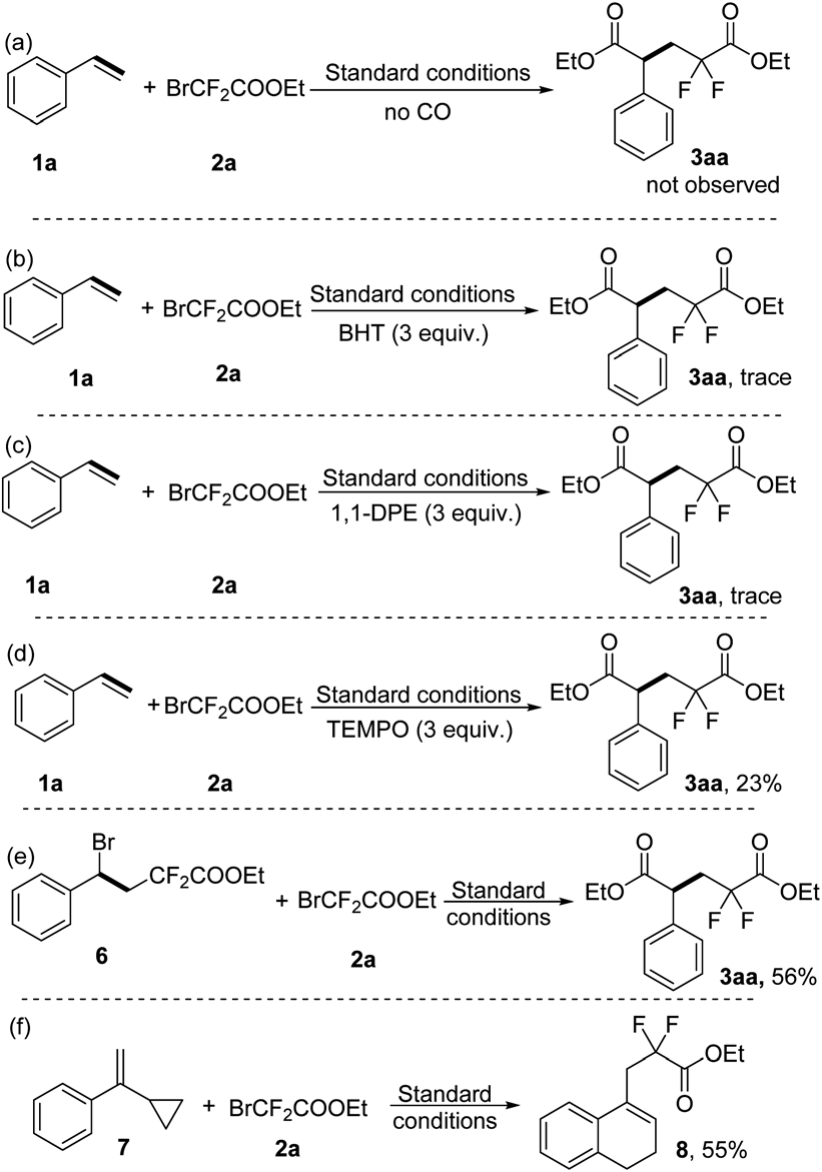
Control experiments.

In order to gain some insight into the reaction mechanism, several control experiments were performed. Firstly, the target product 3aa was not observed in the absence of carbon monoxide gas under the standard conditions (Scheme 5a). Secondly, only a trace amount of the target product 3aa was detected when the radical inhibitor BHT (2,4-di-*tert*-butyl-4-methylphenol, 3 equiv.) or 1, 1-DPE (1,1-diphenylethylene, 3 equiv.) was added to our model reaction under the standard conditions (Scheme 5b and 5c). Similarly, the yield of 3aa was decreased to 23% when the radical inhibitor TEMPO (3 equiv.) was added (Scheme 5d). Furthermore, radical inhibitors 1, 1-DPE and TEMPO both trapped the difluoroacetate radical and detected it in GC-MS (see the ESI†), which indicates that this reaction involves radical intermediates. Moreover, the possible intermediate 6 was prepared and then reacted with ethyl bromodifluoroacetate 2a under standard conditions, and the desired product 3aa was obtained in 56% yield (Scheme 5e). The radical nature of this reaction was also proven by the ring-opening radical clock reaction and 55% of the corresponding product 8 was obtained under our standard conditions (Scheme 5f).

**Scheme 6:**
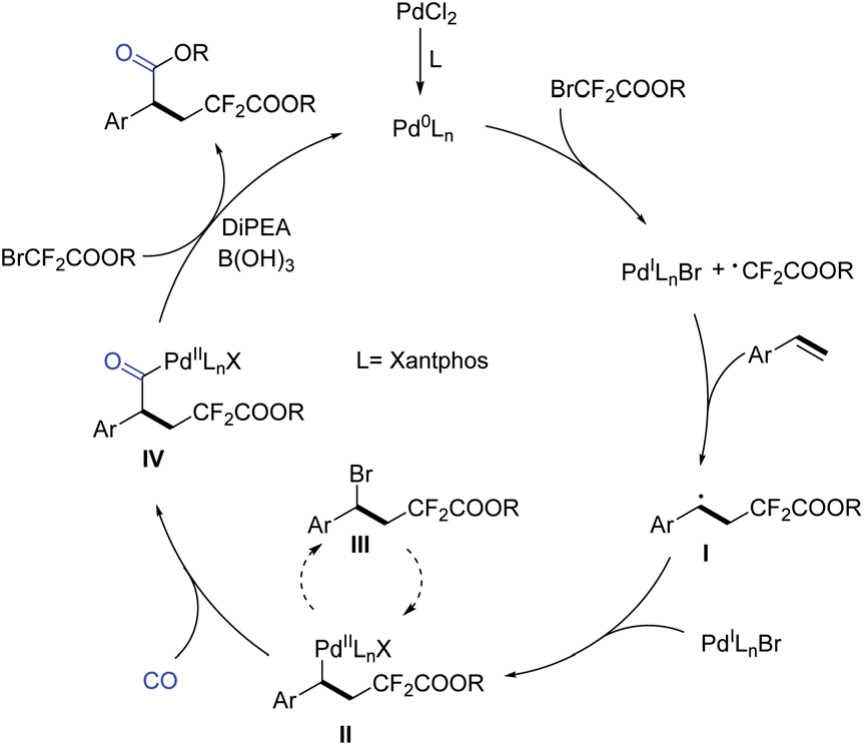
Proposed mechanism.

Based on the above results and literature studies,^13,16^ a plausible reaction mechanism is proposed (Scheme 6). The catalytic cycle starts from the active catalyst Pd^0^Ln species, which was generated from the PdCl_2_ pre-catalyst. Then, the Pd^0^Ln complex induced a SET (single-electron transfer) process of bromodifluoroacetate to give the corresponding difluoroacetate radical and a Pd^I^LnX species, followed by the addition of the difluoroacetate radical to aryl olefin to give a new secondary benzylic radical I. Subsequently, the Pd^I^LnX species was reincorporated with the carbon radical I to afford the key intermediate II. It is important to mention that the complex II can be converted into III through reductive elimination. However, the reaction is reversible and compound III can react with the reactive Pd^0^Ln species and be reconverted into II. After the insertion of carbon monoxide, complex II will be transformed into intermediate IV. Finally, intermediate IV reacts with another molecule of bromodifluoroacetate and gives the desired final product after the reductive elimination procedure. Meanwhile, in the presence of DiPEA, Pd^0^Ln will be regenerated for the next catalytic cycle. Although their roles are not very clear, we believe that B(OH)_3_ can promote the decomposition of bromodifluoroacetate for alcohol release and CsF is more like a buffer here.

## Conclusions

In summary, a novel palladium-catalyzed procedure for difluoroalkylative carbonylation of aryl olefins has been developed. We have overcome the previous inability to take advantage of aryl olefins in difluoroalkylative carbonylation. A variety of aryl olefins were transformed into the corresponding difluoropentanedioate compounds in good yields with broad functional group tolerance and excellent selectivity. Additionally, the scaled-up reaction to a 3 mmol scale can be performed smoothly with a similar yield. Furthermore, the produced difluoropentanedioate product can be efficiently converted to the corresponding diacid and diol in a facile manner.

## Author contributions

XFW surprised this project and revised the manuscirpt. ZPB and YZ performed all the experiments. ZPB prepared the first version of this manuscript.

## Conflicts of interest

There are no conflicts to declare.

## Supplementary Material

SC-013-D2SC02665A-s001

## References

[cit1] Zhou Y., Wang J., Gu Z., Wang S., Zhu W., Aceña J. L., Soloshonok V. A., Izawa K., Liu H. (2016). Chem. Rev..

[cit2] (a) Kirsch P. , Modern fluoroorganic chemistry: synthesis, reactivity, applications, John Wiley & Sons, 2013

[cit3] Carter N. J., Keating G. M. (2007). Drugs.

[cit4] Shweta P., Juhi V., Ravindra D. D., Rikeshwer P. D., Nagashekhara M., Ranjeet A. B., Pravat K. S., Prashant K. (2021). Int. J. Pharm..

[cit5] Deeks E. D. (2016). Drugs.

[cit6] Pantcheva M. B., Seibold L. K., Awadallah N. S., Kahook M. Y. (2011). Adv. Ther..

[cit7] Feng Z., Min Q.-Q., Zhao H.-Y., Gu J.-W., Zhang X. (2015). Angew. Chem., Int. Ed..

[cit8] Kong W., Yu C., An H., Song Q. (2018). Org. Lett..

[cit9] Zhang P., Li W., Qu W., Shu Z., Tao Y., Lin J., Gao X. (2021). Org. Lett..

[cit10] Li J.-L., Liu Y.-Q., Zou W.-L., Zeng R., Zhang X., Liu Y., Han B., He Y., Leng H.-J., Li Q.-Z. (2020). Angew. Chem., Int. Ed..

[cit11] (j) Carbon monoxide in organic synthesis-Carbonylation chemistry, ed., B. Gabriele, Wiley-VCH, 2021

[cit12] Qi X., Yu F., Chen P., Liu G. (2017). Angew. Chem., Int. Ed..

[cit13] Zhang Y., Geng H.-Q., Wu X.-F. (2021). Angew. Chem., Int. Ed..

[cit14] Wu F.-P., Yuan Y., Wu X.-F. (2021). Angew. Chem., Int. Ed..

[cit15] Ai H.-J., Cai C.-X., Qi X., Peng J.-B., Zheng F., Wu X.-F. (2017). Tetrahedron Lett..

[cit16] Yin H., Skrydstrup T. (2017). J. Org. Chem..

