# Supplementary material for: Palladium-catalyzed difluoroalkylative carbonylation of styrenes toward difluoropentanedioates
Source: Chem Sci. 2022 Aug 3;13(32):9387–91. doi: 10.1039/d2sc02665a (PMC9384137; doi:10.1039/d2sc02665a)

Electronic Supplementary Material (ESI) for Chemical Science.
This journal is © The Royal Society of Chemistry 2022

Supporting Information

Palladium-Catalyzed Difluoroalkylative Carbonylation of Styrenes Toward Difluoropentanedioates

Zhi-Peng Bao,[a,b] Youcan Zhang,[a] Xiao-Feng Wu*[a,b]

a. Dalian National Laboratory for Clean Energy, Dalian Institute of Chemical Physics, Chinese Academy of Sciences, 116023 Dalian, Liaoning, China, E-mail: xwu2020@dicp.ac.cn

b. Leibniz-Institut für Katalyse e.V., Albert-Einstein-Straße 29a, 18059 Rostock, Germany, E-mail: Xiao-Feng.Wu@catalysis.de

Table of Contents

1. General Information......1
2. Optimization of Reaction Conditions......1
3. Preparation of 1p, 1q, 6, 1b......5
   3.1 Preparation of 1p, 1q. 5
   3.2 Preparation of 1b......5
   3.3 Preparation of 6. 6
4. General Procedure......6
5. Scale-up reaction and transformation of the product 3aa. 7
6. Mechanistic Studies. 8
7. Characterization of substrates and Products......10
   7.1 substrates......10
   7.2 Products 10
8. References......22
9. Copy of ^{1}H and ^{13}C NMR Spectra of Substrates and Products......23
   9.1 Substrates......23
   9.2 Products. 26

1. General Information

Unless otherwise noted, all reactions were carried out under a carbon monoxide or nitrogen atmosphere. All reagents were from commercial sources, all solvents are extra dry solvents and used as received without further purification. Column chromatography was performed on silica gel (200-300 meshes) using petroleum ether (b.p. 60-90 °C) and ethyl acetate as the eluents. ^{1}H and ^{13}C NMR spectra were taken on Bruker AVANCE III 400 MHz or 700 MHz spectrometers and spectral data were reported in ppm relative to tetramethylsilane (TMS) as the internal standard and $CDCl_3$ or DMSO-D_6 as solvent. All coupling constants (J) are reported in Hz with the following abbreviations: s = singlet, d = doublet, dd = double doublet, t = triplet, dt = double triplet, q = quatriplet, m = multiplet, br = broad. Gas chromatography (GC) analyses were performed on an Agilent HP-7890A instrument with a FID detector and HP-5 capillary column (polydimethylsiloxane with 5% phenyl groups, 30 m, 0.32 mm i.d. 0.25 μm film thickness) using argon as carrier gas. Gas chromatography mass spectrometer (GC-MS) analyses were performed on a Shimadzu QP2020 NX instrument. High resolution mass spectra (HRMS) were recorded on Agilent 8890-7250 and Agilent Q-TOF 6540. Because of the high toxicity of carbon monoxide, all of the reactions should be performed in an autoclave. The laboratory should well-equipped with a CO detector and alarm system.

2. Optimization of Reaction Conditions

Table S1. Optimization type of base.

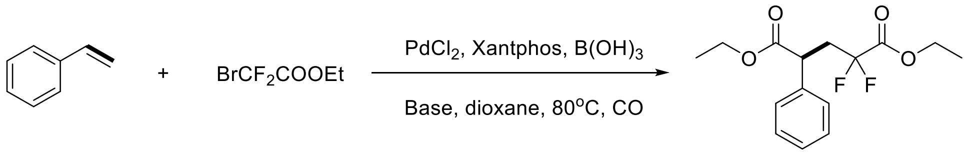

1a **2a** **3aa**

Entry	Base	**3aa** (%)^{b}
1	Na_3PO_4	7
2	Na_2CO_3	19
3	NaO^{t}Bu	N.D.
4	DiPEA	57

Reaction conditions: **1a** (0.3 mmol), **2a** (0.9 mmol), $PdCl_2$ (10 mol%), Xantphos (10 mol%), $B(OH)_3$ (0.2 mmol), Base (1.2 mmol), dioxane (1.5 mL), CO (10 bar), 80 °C, 18 h, ^{b}GC yield.

Table S2. Optimization amount of base.

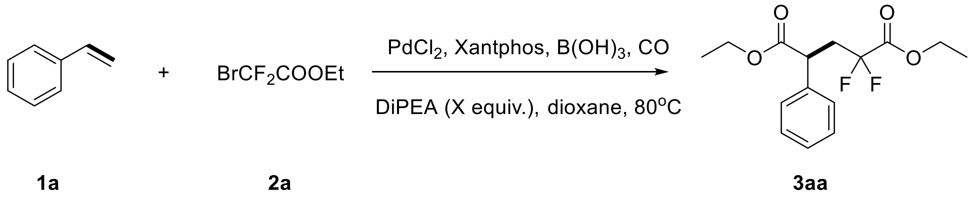

Entry	DiPEA (X mmol)	**3aa** (%)^b
1	0.9 mmol	39
2	1.2 mmol	57
3	1.5 mmol	50

Reaction conditions: **1a** (0.3 mmol), **2a** (0.9 mmol), $PdCl_2$ (10 mol%), Xantphos (10 mol%), $B(OH)_3$ (0.2 mmol), DiPEA (X mmol), dioxane (1.5 mL), CO (10 bar), 80 °C, 18 h, ^{b}GC yield.

Table S3. Optimization of pallidum source.

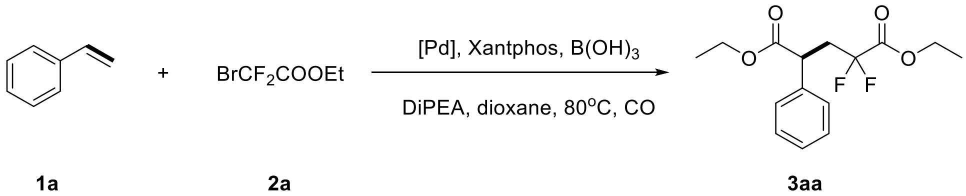

Entry	[Pd]	**3aa** (%)^b
1	$Pd(OAc)_2$	50
2	$Pd(PPh_3)_4$	46
3	$Pd(PPh_3)_2Cl_2$	47
4	$Pd(TFA)_2$	46
5	$[PdCl(allyl)]_2$	32
6	$Pd_2(dba)_3$	44
7	$PdCl_2$	57

Reaction conditions: **1a** (0.3 mmol), **2a** (0.9 mmol), [Pd] (10 mol%), Xantphos (10 mol%), $B(OH)_3$ (0.2 mmol), DiPEA (1.2 mmol), dioxane (1.5 mL), CO (10 bar), 80 °C, 18 h, ^{b}GC yield.

Table S4. Optimization amount of $B(OH)_3$.

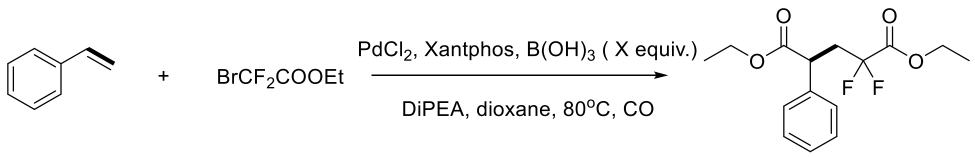

1a **2a** **3aa**

Entry	$B(OH)_3$	**3aa** (%)[b]
1	0	17
2	0.1 mmol	22
3	0.3 mmol	57
4	0.4 mmol	60
5	0.5 mmol	53

Reaction conditions: **1a** (0.3 mmol), **2a** (0.9 mmol), $PdCl_2$ (10 mol%), Xantphos (10 mol%), $B(OH)_3$ (X mmol), DiPEA (1.2 mmol), dioxane (1.5 mL), CO (10 bar), 80 °C, 18 h, [b]GC yield.

Table S5. Optimization type of solvent.

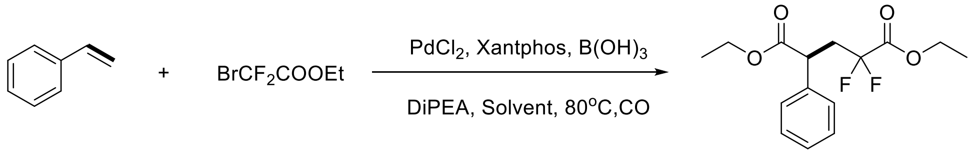

1a **2a** **3aa**

Entry	Solvent	**3aa** (%)[b]
1	dioxane	60
2	DCE	45
3	DMF	47
4	DMSO	49
5	MeCN	66
6	Toluene	46
7	THF	42

Reaction conditions: **1a** (0.3 mmol), **2a** (0.9 mmol), $PdCl_2$ (10 mol%), Xantphos (10 mol%), $B(OH)_3$ (0.4 mmol), DiPEA (1.2 mmol), Solvent (1.5 mL), CO (10 bar), 80 °C, 18 h, [b]GC yield.

Table S5. Optimization type of Ligand.

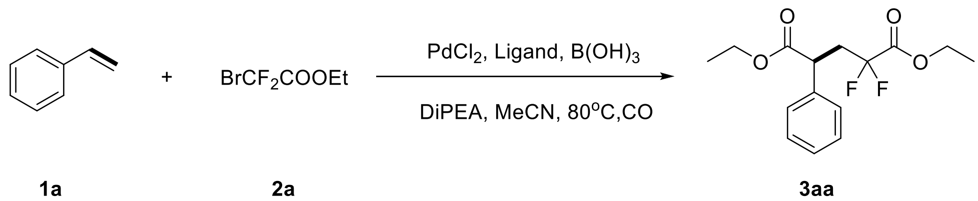

Entry	Ligand	**3aa** (%)^b
1	PPh_3	50
2	DPEphos	18
3	Nixantphos	46
4	DPPP	trace
5	DPPF	trace
6	Xantphos	66

Reaction conditions: **1a** (0.3 mmol), **2a** (0.9 mmol), $PdCl_2$ (10 mol%), Monodentate ligand (20 mol%) or Bidentate ligand (10 mol%), $B(OH)_3$ (1.3 mmol), DiPEA (1.2 mmol), dioxane (1.5 mL), CO (10 bar), 80 °C, 18 h, ^{b}GC yield.

Table S6. Optimization of pressure of CO.

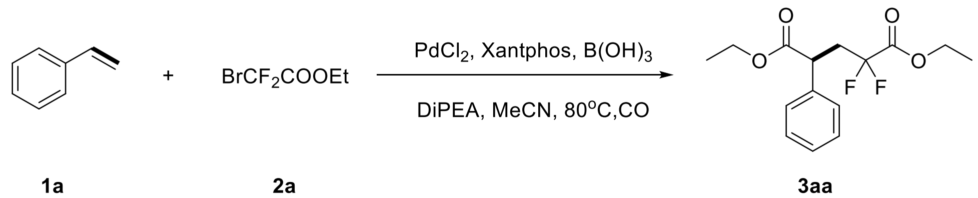

Entry	CO (X bar)	**3aa** (%)^b
1	1	26
2	5	67
3	10	66

Reaction conditions: **1a** (0.3 mmol), **2a** (0.9 mmol), $PdCl_2$ (10 mol%), Xantphos (10 mol%), $B(OH)_3$ (0.4 mmol), DiPEA (1.2 mmol), MeCN (1.5 mL), CO (X bar), 80 °C, 18 h, ^{b}GC yield.

Table S7. Optimization of additive.

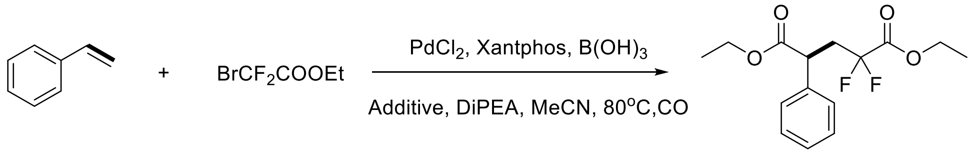

1a **2a** **3aa**

Entry	Additive	**3aa** (%)^b
1	NaI	67
2	KI	60
3	NaF	67
4	CsF	81 (78%)

Reaction conditions: **1a** (0.3 mmol), **2a** (0.9 mmol), $PdCl_2$ (10 mol%), Xantphos (10 mol%), $B(OH)_3$ (0.4 mmol), DiPEA (1.2 mmol), Additive (1 equiv.), MeCN (1.5 mL), CO (5 bar), 80 °C, 18 h, ^{b}GC yield.

3. Preparation of 1p, 1q, 6, 1b.

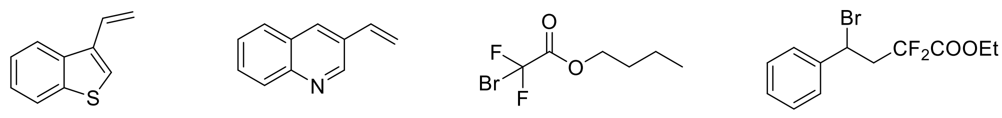

1p **1q** **2b** **6**

Note: Alkenes **1a-o**, **1r-1t** are commercially available.

3.1 Preparation of 1p, 1q.[1]

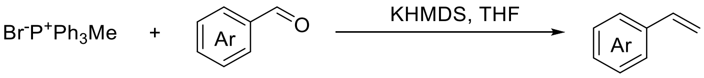

To a suspension of methyltriphenylphosphonium bromide (5.5 mmol) in THF was slowly added potassium bis(trimethylsilyl)amide while maintaining the internal temperature at <5 °C. Aryl aldehyde (5 mmol) was then added in portions. The progress was monitored by TLC. When the reaction was complete, it was quenched with saturated NH_4Cl (10 mL) and water (10 mL). The organic layer was washed with saturated NaCl (10 mL) and concentrated. The desired products **1p** and **1q** were isolated by silica gel chromatography as colorless oils.

3.2 Preparation of 2b.[2]

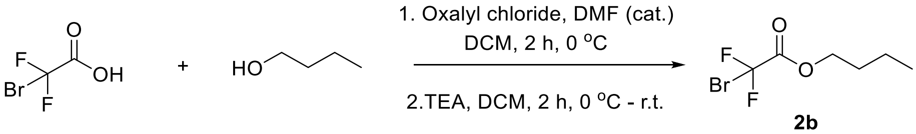

2b

α-Bromo-*α, α*-difluoroacetic acid (1 equiv.) was dissolved in dry DCM in a dry round bottom flask under an atmosphere of nitrogen. Oxalyl chloride (1.1 equiv) was then added followed by two drops of DMF. The solution was then stirred at room temperature for 2 hours or until no more gas evolved. The

n-butanol (2 equiv.) and triethylamine (1.1 equiv.) were then added dropwise as a solution in DCM at 0 °C over 20 min. The cooling was removed and stirring was continued for 2 hours. The crude reaction was then diluted with water and extracted three times with DCM. The combined organic phases were washed with saturated bicarbonate and dried over $MgSO_4$, filtered and concentrated in vacuo. The resulting residue was then filtered through a short plug of silica (eluent: pentane) and the fractions containing product were concentrated to afford **2b** as a colorless oil.

3.3 Preparation of 6.[3]

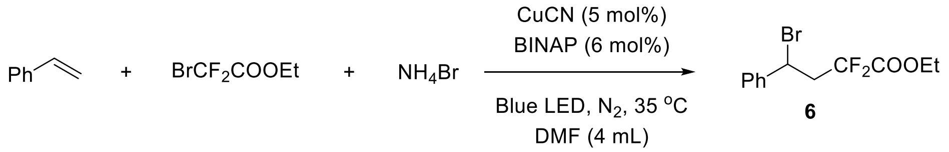

Into a 15 mL Schlenk tube were added CuCN (1.8 mg, 5 mol %), BINAP (14.9 mg, 6 mol %), NH_4Br (78.3 mg, 0.8 mmol,) and DMF (4 mL) under a N_2 atmosphere. Then the alkene Styrene (41.6 mg, 0.4 mmol) and $BrCF_2COOEt$ (203.0 mg, 1.0 mmol) were added. The resulting mixture was stirred at 35 °C and irradiated with blue LEDs for 12 h under a N_2 atmosphere. When the reaction was completed, the crude reaction mixture was diluted with EA (20 mL). The solution was washed with water (3×20 mL) and brine (20 mL), and then dried over anhydrous Na_2SO_4 and evaporated under reduced pressure. The residue was subjected to flash column chromatography (1%→2% ethyl acetate/petroleum ether) to give the final product **6** as a colorless oil.

4. General Procedure

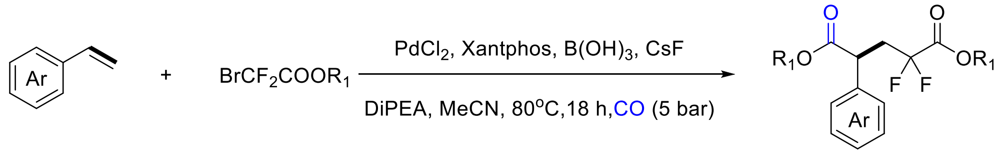

A 4 mL screw-cap vial was charged with $PdCl_2$ (10 mol%, 5.3 mg), Xantphos (10 mol%, 17.4 mg), $B(OH)_3$ (0.4 mmol, 24.7 mg), CsF (0.3 mmol, 45.6 mg) and an oven-dried stirring bar. The vial was closed with a Teflon septum and cap and connected to the atmosphere via a needle. Then aryl olefins (0.3 mmol), bromodifluoroacetate (0.9 mmol), DiPEA (1.2 mmol, 155.1 mg), MeCN (1.5 mL) was added with a syringe under N_2 atmosphere, the vial was moved to an alloy plate and put into a Parr 4560 series autoclave (300 mL) under N_2 atmosphere. At room temperature, the autoclave was flushed with CO three times and charged with 5 bar CO. The autoclave was placed on a heating plate equipped with a magnetic stirrer. The reaction mixture was heated to 80 °C for 18 h. After the reaction was completed, the crude mixture was filtered and concentrated under vacuum. The crude product was purified by column chromatography (PE/EA=50/1 to 20/1) on silica gel to afford the products.

5. Scale-up reaction and transformation of the product 3aa.

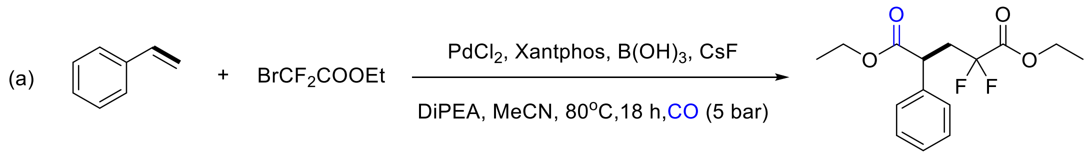

3 mmol **3aa:** 0.57 g, 63%

(a) A 30 mL screw-cap vial was charged with $PdCl_2$ (10 mol%, 53 mg), Xantphos (10 mol%, 174 mg), $B(OH)_3$ (4 mmol, 247 mg), CsF (3 mmol, 456 mg) and an oven-dried stirring bar. The vial was closed with a Teflon septum and cap and connected to the atmosphere via a needle. Then aryl olefins (3 mmol), bromodifluoroacetate (9 mmol), DiPEA (12 mmol, 1551 mg), MeCN (15 mL) was added with a syringe under N_2 atmosphere, the vial was moved to an alloy plate and put into a Parr 4560 series autoclave (300 mL) under N_2 atmosphere. At room temperature, the autoclave was flushed with CO three times and charged with 5 bar CO. The autoclave was placed on a heating plate equipped with a magnetic stirrer. The reaction mixture was heated to 80 °C for 18 h. After the reaction was completed, the crude mixture was filtered and concentrated under vacuum. The crude product was purified by column chromatography (PE/EA=50/1 to 20/1) on silica gel to afford the product **3aa** (570 mg, 63%).

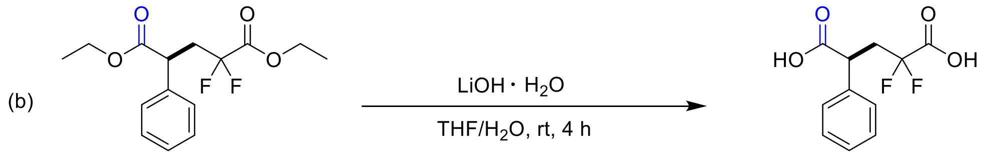

3aa **4,** 92%

(b) 3aa (0.3 mmol) and LiOH-H_2O (5.1 mmol) were stirred in a THF/H_2O (2.5:1) mixture at room temperature for 4 h. The mixture was then acidified to pH 1 with a 35% HCl solution and extracted with EtOAc (3× 10 mL), and the combined organic layer was dried over Na_2SO_4, and purified by column chromatography (PE/EA= 1/1) on silica gel to afford the product **4** (67.3 mg, 92%).

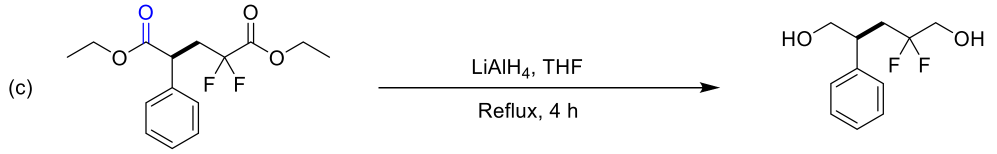

3aa **5,** 98%

(c) To a stirred cold suspension (ice bath) of **3aa** (0.6 mmol) in dry THF (1 mL) was added $LiAlH_4$ (1.2 mL, 1.0 mol/L in THF) slowly under N_2. The mixture was slowly warmed to the refluxing temperature for 4 h. The mixture was then cooled down to room temperature and quenched by addition of MeOH (1 mL) followed by addition of aq. solution of NaOH (10%, 1.6 mL). After the mixture was filtered through a pad of celite, THF was evaporated from the filtrate in vacuum. The resultant mixture was extracted with CH_2Cl_2 (3 × 5 mL). The combined organic layers were washed with brine (2 × 5 mL) and dried over $MgSO_4$. The solvent was removed in vacuum and the residue was purified by flash chromatography (PE/EA=2:1) to afford the desired product **5** (127.0 mg, 98%).

6. Mechanistic Studies.

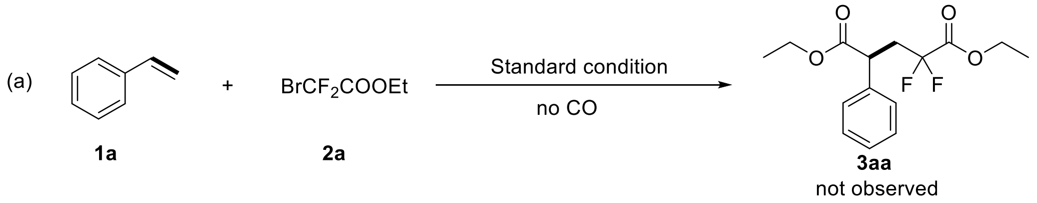

A 4 mL screw-cap vial was charged with $PdCl_2$ (10 mol%, 5.3 mg), Xantphos (10 mol%, 17.4 mg), $B(OH)_3$ (0.4 mmol, 24.7 mg), CsF (0.3 mmol, 45.6 mg) and an oven-dried stirring bar. The vial was closed with a Teflon septum and cap and connected to the atmosphere via a needle. Then aryl olefins (0.3 mmol), bromodifluoroacetate (0.9 mmol), DiPEA (1.2 mmol, 155.1 mg), MeCN (1.5 mL) was added with a syringe under N_2 atmosphere, the vial was moved to an alloy plate and put into a Parr 4560 series autoclave (300 mL) under N_2 atmosphere. At room temperature, the autoclave was flushed with N_2 three times. The autoclave was placed on a heating plate equipped with a magnetic stirrer. The reaction mixture was heated to 80 °C for 18 h. And **3aa** was not observed.

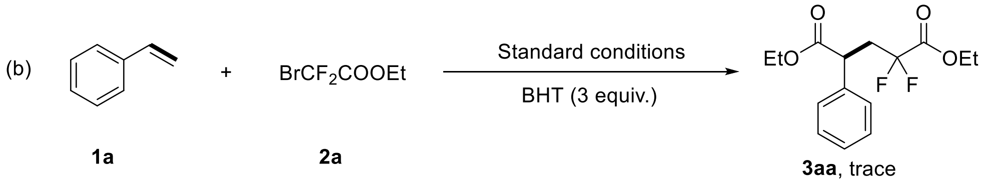

(b) A 4 mL screw-cap vial was charged with BHT (0.9 mmol, 198.2 mg), $PdCl_2$ (10 mol%, 5.3 mg), Xantphos (10 mol%, 17.4 mg), $B(OH)_3$ (0.4 mmol, 24.7 mg), CsF (0.3 mmol, 45.6 mg) and an oven-dried stirring bar. The vial was closed with a Teflon septum and cap and connected to the atmosphere via a needle. Then aryl olefins (0.3 mmol), bromodifluoroacetate (0.9 mmol), DiPEA (1.2 mmol, 155.1 mg), MeCN (1.5 mL) was added with a syringe under N_2 atmosphere, the vial was moved to an alloy plate and put into a Parr 4560 series autoclave (300 mL) under N_2 atmosphere. At room temperature, the autoclave was flushed with CO three times and charged with 5 bar CO. The autoclave was placed on a heating plate equipped with a magnetic stirrer. The reaction mixture was heated to 80 °C for 18 h. And **3aa** was detected trace (GC yield).

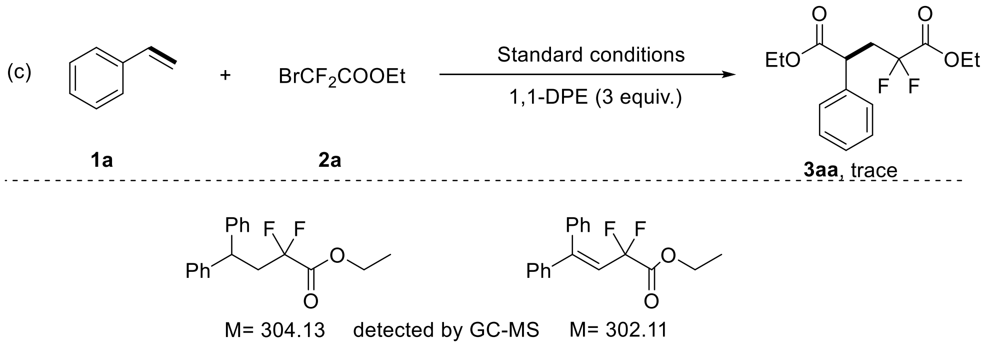

(c) A 4 mL screw-cap vial was charged with TEMPO (0.9 mmol, 140.6 mg), $PdCl_2$ (10 mol%, 5.3 mg), Xantphos (10 mol%, 17.4 mg), $B(OH)_3$ (0.4 mmol, 24.7 mg), CsF (0.3 mmol, 45.6 mg) and an oven-dried stirring bar. The vial was closed with a Teflon septum and cap and connected to the atmosphere via a needle. Then aryl olefins (0.3 mmol), bromodifluoroacetate (0.9 mmol), DiPEA (1.2 mmol, 155.1 mg), MeCN (1.5 mL) was added with a syringe under N_2 atmosphere, the vial was moved to an alloy plate and put into a Parr 4560 series autoclave (300 mL) under N_2 atmosphere. At room

temperature, the autoclave was flushed with CO three times and charged with 5 bar CO. The autoclave was placed on a heating plate equipped with a magnetic stirrer. The reaction mixture was heated to 80 °C for 18 h. And **3aa** was detected 23% yield (GC yield).

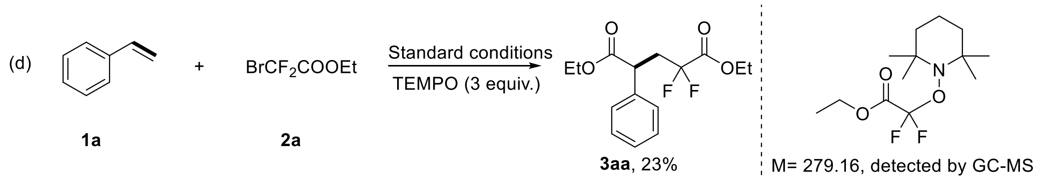

(d) A 4 mL screw-cap vial was charged with 1, 1-DPE (0.9 mol, 162.1 mg), $PdCl_2$ (10 mol%, 5.3 mg), Xantphos (10 mol%, 17.4 mg), $B(OH)_3$ (0.4 mmol, 24.7 mg), CsF (0.3 mmol, 45.6 mg) and an oven-dried stirring bar. The vial was closed with a Teflon septum and cap and connected to the atmosphere via a needle. Then aryl olefins (0.3 mmol), bromodifluoroacetate (0.9 mmol), DiPEA (1.2 mmol, 155.1 mg), MeCN (1.5 mL) was added with a syringe under N_2 atmosphere, the vial was moved to an alloy plate and put into a Parr 4560 series autoclave (300 mL) under N_2 atmosphere. At room temperature, the autoclave was flushed with CO three times and charged with 5 bar CO. The autoclave was placed on a heating plate equipped with a magnetic stirrer. The reaction mixture was heated to 80 °C for 18 h. And **3aa** was detected trace (GC yield).

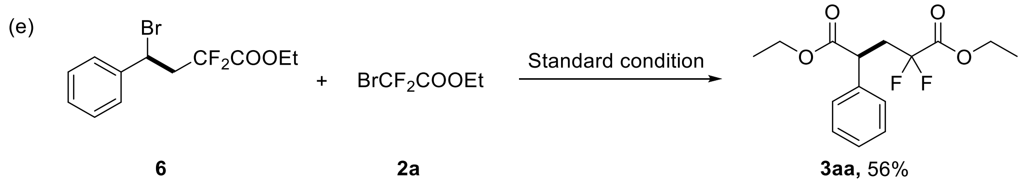

(e) A 4 mL screw-cap vial was charged with $PdCl_2$ (10 mol%, 5.3 mg), Xantphos (10 mol%, 17.4 mg), $B(OH)_3$ (0.4 mmol, 24.7 mg), CsF (0.3 mmol, 45.6 mg) and an oven-dried stirring bar. The vial was closed with a Teflon septum and cap and connected to the atmosphere via a needle. Then **6** (0.3 mmol), **2a** (0.9 mmol), DiPEA (1.2 mmol, 155.1 mg), MeCN (1.5 mL) was added with a syringe under N_2 atmosphere, the vial was moved to an alloy plate and put into a Parr 4560 series autoclave (300 mL) under N_2 atmosphere. At room temperature, the autoclave was flushed with CO three times and charged with 5 bar CO. The autoclave was placed on a heating plate equipped with a magnetic stirrer. The reaction mixture was heated to 80 °C for 18 h. And **3aa** was detected 56% (GC yield).

7. Characterization of Substrates and Products

7.1 Substrates

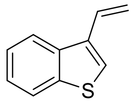

3-vinylbenzo[b]thiophene (**1p**)[4]

^{1}H NMR (400 MHz, $CDCl_3$) δ 7.99 (d, J = 7.7 Hz, 1H), 7.92 (d, J = 7.6 Hz, 1H), 7.51 (s, 1H), 7.50 – 7.39 (m, 2H), 7.04 (dd, J = 17.6, 11.1 Hz, 1H), 5.87 (dd, J = 17.6, 1.1 Hz, 1H), 5.45 (dd, *J* = 11.1, 1.1 Hz, 1H).

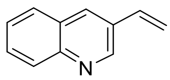

3-vinylquinoline (**1q**)[1]

^{1}H NMR (400 MHz, $CDCl_3$) δ 8.95 (d, J = 2.0 Hz, 1H), 8.02 (d, J = 8.4 Hz, 1H), 7.90 (d, J = 1.6 Hz, 1H), 7.65 (d, J = 8.2 Hz, 1H), 7.61 – 7.53 (m, 1H), 7.41 (t, J = 7.5 Hz, 1H), 6.74 (dd, J = 17.7, 11.0 Hz, 1H), 5.87 (d, J = 17.7 Hz, 1H), 5.35 (d, J = 11.0 Hz, 1H).

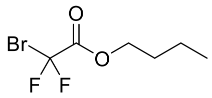

Butyl 2-bromo-2,2-difluoroacetate (**2b**)[2]

^{1}H NMR (400 MHz, $CDCl_3$) δ 4.35 (t, J = 6.6 Hz, 2H), 1.81 – 1.65 (m, 2H), 1.43 (dd, J = 15.0, 7.5 Hz, 2H), 0.95 (t, J = 7.4 Hz, 3H).

^{13}C NMR (101 MHz, $CDCl_3$) δ 159.7 (t, *J* = 31.2 Hz), 108.8 (t, *J* = 314.3 Hz), 68.2, 30.1, 18.8, 13.5.

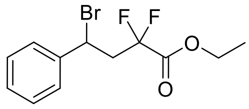

Ethyl 4-bromo-2,2-difluoro-4-phenylbutanoate (**6**)[3]

^{1}H NMR (400 MHz, $CDCl_3$) δ 7.41 (d, *J* = 7.4 Hz, 2H), 7.35 (t, *J* = 7.5 Hz, 2H), 7.31 (t, *J* = 7.3 Hz, 1H), 5.17 (t, *J* = 7.3 Hz, 1H), 4.16 – 4.06 (m, 2H), 3.24 – 3.06 (m, 2H), 1.28 (t, *J* = 7.2 Hz, 3H).

7.2 Products

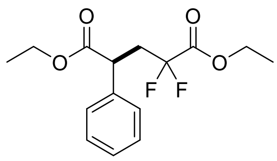

Diethyl 2,2-difluoro-4-phenylpentanedioate (**3aa**)

The title compound was prepared from styrene (31.2 mg, 0.3 mmol) and ethyl bromodifluoroacetate (181.7 mg, 0.9 mmol) according to general procedure. The crude residue was purified by flash chromatography (PE/EA = 50:1 to 20:1) to give the product as a colorless oil (70.2 mg, 78%).

^{1}H NMR (400 MHz, $CDCl_3$) δ 7.35 – 7.24 (m, 5H), 4.23 – 4.02 (m, 4H), 3.91 (dd, *J* = 8.6, 5.2 Hz, 1H), 3.14 – 2.97 (m, 1H), 2.57 – 2.41 (m, 1H), 1.28 (t, *J* = 7.2 Hz, 3H), 1.18 (t, *J* = 7.1 Hz, 3H).

^{13}**C NMR (101 MHz, CDCl$_3$)** δ 172.3, 163.5 (t, J = 32.4 Hz), 137.6, 128.8, 127.7, 114.9 (t, J = 251.0 Hz), 62.9, 61.3, 44.8 (t, J = 4.2 Hz), 37.9 (t, J = 23.4 Hz), 13.8, 13.7.

^{19}**F NMR (56 MHz, CDCl$_3$)** δ -109.9 – -110.2 (m, 2F).

HRMS (EI): Calcd. for $C_{15}H_{18}F_2O_4$ [M+H]$^+$ 300.1246, found: 300.1249.

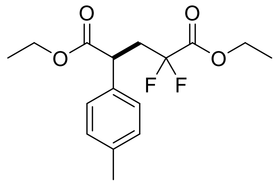

Diethyl 2,2-difluoro-4-(p-tolyl)pentanedioate (**3ba**)

The title compound was prepared from 1-methyl-4-vinylbenzene (35.4 mg, 0.3 mmol) and ethyl bromodifluoroacetate (181.7 mg, 0.9 mmol) according to general procedure. The crude residue was purified by flash chromatography (PE/EA = 50:1 to 20:1) to give the product as a colorless oil (68.8 mg, 73%).

^{1}**H NMR (400 MHz, CDCl$_3$)** δ 7.18 (d, J = 8.1 Hz, 2H), 7.13 (d, J = 8.1 Hz, 2H), 4.24 – 4.01 (m, 4H), 3.87 (dd, J = 8.6, 5.1 Hz, 1H), 3.11 – 2.95 (m, 1H), 2.55 – 2.39 (m, 1H), 2.32 (s, 3H), 1.30 (t, J = 7.2 Hz, 3H), 1.19 (t, J = 7.1 Hz, 3H).

^{13}**C NMR (101 MHz, CDCl$_3$)** δ 172.5, 163.6 (t, J = 32.4 Hz), 137.5, 134.6, 129.5, 127.6, 115.0 (t, J = 250.9 Hz), 62.9, 61.3, 44.5 (t, J = 4.2 Hz), 38.0 (t, J = 23.3 Hz), 21.0, 13.9, 13.8.

^{19}**F NMR (56 MHz, CDCl$_3$)** δ -110.0 – -110.3 (m, 2F).

HRMS (EI): Calcd. for $C_{16}H_{20}F_2O_4$ [M+H]$^+$ 315.1402, found: 315.1405.

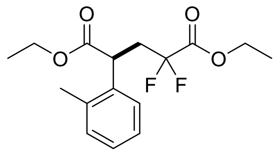

Diethyl 2,2-difluoro-4-(*o*-tolyl)pentanedioate (**3ca**)

The title compound was prepared from 1-methyl-2-vinylbenzene (35.4 mg, 0.3 mmol) and ethyl bromodifluoroacetate (181.7 mg, 0.9 mmol) according to general procedure. The crude residue was purified by flash chromatography (PE/EA = 50:1 to 20:1) to give the product as a colorless oil (42.4 mg, 45%).

^{1}**H NMR (400 MHz, CDCl$_3$)** δ 7.25 – 7.13 (m, 4H), 4.22 – 4.02 (m, 5H), 3.15 – 2.98 (m, 1H), 2.51 – 2.35 (m, 4H), 1.29 (t, J = 7.1 Hz, 1H), 1.18 (t, J = 7.1 Hz, 1H).

^{13}**C NMR (101 MHz, CDCl$_3$)** δ 172.7, 163.7 (t, J = 32.5 Hz), 136.2, 136.2, 130.8, 127.6, 126.7, 126.5, 115.1 (t, J = 250.8 Hz), 63.0, 61.3, 40.3 (t, J = 4.2 Hz), 37.6 (t, J = 23.6 Hz), 19.6, 14.0, 13.8.

^{19}**F NMR (56 MHz, CDCl3)** δ -109.2 – -109.4 (m, 2F).

HRMS (EI): Calcd. for $C_{16}H_{20}F_2O_4$ [M+H]$^+$ 315.1402, found: 315.1393.

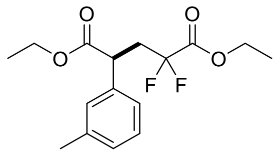

Diethyl 2,2-difluoro-4-(*m*-tolyl)pentanedioate (**3da**)

The title compound was prepared from 1-methyl-3-vinylbenzene (35.4 mg, 0.3 mmol) and ethyl bromodifluoroacetate (181.7 mg, 0.9 mmol) according to general procedure. The crude residue was purified by flash chromatography (PE/EA = 50:1 to 20:1) to give the product as a colorless oil (37.7 mg, 40%).

^{1}H NMR (400 MHz, CDCl$_3$) δ 7.21 (t, J = 7.4 Hz, 1H), 7.09 (d, J = 8.0 Hz, 3H), 4.27 – 4.02 (m, 4H), 3.86 (dd, J = 8.7, 4.9 Hz, 1H), 3.15 – 2.96 (m, 1H), 2.55 – 2.39 (m, 1H), 2.34 (s, 3H), 1.30 (t, J = 7.1 Hz, 3H), 1.20 (t, J = 7.1 Hz, 3H).

^{13}C NMR (101 MHz, CDCl$_3$) δ 172.5, 163.7 (t, J = 32.5 Hz), 138.6, 137.6, 128.7, 128.5, 128.4, 124.8, 115.0 (t, J = 251.0 Hz), 63.0, 61.3, 44.8 (t, J = 4.2 Hz), 38.0 (t, J = 23.4 Hz), 21.4, 14.0, 13.8.

^{19}F NMR (56 MHz, CDCl$_3$) δ -109.7 – -110.0 (m, 2F).

HRMS (EI): Calcd. for $C_{16}H_{20}F_2O_4$ $[M+H]^+$ 315.1402, found: 315.1392.

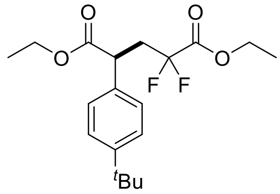

Diethyl 4-(4-(*tert*-butyl)phenyl)-2,2-difluoropentanedioate (**3ea**)

The title compound was prepared from 1-(*tert*-butyl)-4-vinylbenzene (48.0 mg, 0.3 mmol) and ethyl bromodifluoroacetate (181.7 mg, 0.9 mmol) according to general procedure. The crude residue was purified by flash chromatography (PE/EA = 50:1 to 20:1) to give the product as a colorless oil (90 mg, 84%).

^{1}H NMR (400 MHz, CDCl$_3$) δ 7.34 (d, J = 8.3 Hz, 2H), 7.22 (d, J = 8.3 Hz, 2H), 4.22 – 4.02 (m, 4H), 3.88 (dd, J = 8.7, 5.1 Hz, 1H), 3.12 – 2.96 (m, 1H), 2.57 – 2.40 (m, 1H), 1.31 – 1.26 (m, 12H), 1.21 (t, J = 7.1 Hz, 3H).

^{13}C NMR (101 MHz, CDCl$_3$) δ 172.5, 163.7 (t, J = 32.5 Hz), 150.7, 134.5, 127.4, 125.8, 115.1 (t, J = 250.9 Hz), 62.9, 61.3, 44.4 (t, J = 4.2 Hz), 38.1 (t, J = 23.5 Hz), 31.4, 31.3, 14.0, 13.8.

^{19}F NMR (56 MHz, CDCl$_3$) δ -109.8 – -110.2 (m, 2F).

HRMS (EI): Calcd. for $C_{19}H_{26}F_2O_4$ $[M+NH_4]^+$ 374.2137, found: 374.2139.

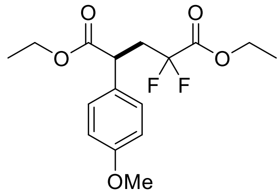

Diethyl 2,2-difluoro-4-(4-methoxyphenyl)pentanedioate (**3fa**)

The title compound was prepared from 1-methoxy-4-vinylbenzene (42.2 mg, 0.3 mmol) and ethyl bromodifluoroacetate (181.7 mg, 0.9 mmol) according to general procedure. The crude residue was purified by flash chromatography (PE/EA = 50:1 to 20:1) to give the product as a colorless oil (87.1 mg,

88%).

^{1}H NMR (400 MHz, $CDCl_3$) δ 7.21 (d, *J* = 8.6 Hz, 2H), 6.84 (d, *J* = 8.7 Hz, 2H), 4.25 – 4.01 (m, 4H), 3.84 (dd, *J* = 8.4, 5.4 Hz, 1H), 3.78 (s, 3H), 3.10 – 2.93 (m, 1H), 2.56 – 2.39 (m, 1H), 1.29 (t, *J* = 7.2 Hz, 3H), 1.19 (t, *J* = 7.1 Hz, 3H).

^{13}C NMR (101 MHz, $CDCl_3$) δ 172.6, 163.6 (t, *J* = 32.4 Hz), 159.1, 129.5, 128.8, 115.0 (t, *J* = 251.0 Hz), 114.1, 62.9, 61.2, 55.2, 44.0 (t, *J* = 4.2 Hz), 38.0 (t, *J* = 23.4 Hz) 13.9, 13.7.

^{19}F NMR (56 MHz, $CDCl_3$) δ -109.8 – -110.6 (m, 2F).

HRMS (EI): Calcd. for $C_{16}H_{20}F_2O_5$ $[M+H]^+$ 331.1352, found: 331.1348.

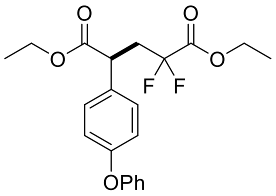

Diethyl 2, 2-difluoro-4-(4-phenoxyphenyl)pentanedioate (**3ga**)

The title compound was prepared from 1-phenoxy-4-vinylbenzene (58.8 mg, 0.3 mmol) and ethyl bromodifluoroacetate (181.7 mg, 0.9 mmol) according to general procedure. The crude residue was purified by flash chromatography (PE/EA = 50:1 to 20:1) to give the product as a colorless oil (88.2 mg, 75%).

^{1}H NMR (400 MHz, $CDCl_3$) δ 7.33 (t, *J* = 7.9 Hz, 2H), 7.25 (d, *J* = 8.6 Hz, 2H), 7.11 (t, *J* = 7.4 Hz, 1H), 7.00 (d, *J* = 7.8 Hz, 2H), 6.95 (d, *J* = 8.6 Hz, 2H), 4.27 – 4.04 (m, 4H), 3.89 (dd, *J* = 8.6, 5.2 Hz, 1H), 3.13 – 2.95 (m, 1H), 2.57 – 2.34 (m, 1H), 1.31 (t, *J* = 7.1 Hz, 3H), 1.21 (t, *J* = 7.1 Hz, 3H).

^{13}C NMR (101 MHz, $CDCl_3$) δ 172.4, 163.6 (t, *J* = 32.4 Hz), 157.0, 156.7, 132.1, 129.7, 129.1, 123.5, 119.1, 118.8, 114.9 (t, *J* = 251.1 Hz), 62.9, 61.3, 44.2 (t, *J* = 4.1 Hz), 37.9 (t, *J* = 23.4 Hz), 13.9, 13.8.

^{19}F NMR (56 MHz, $CDCl_3$) δ -109.9– -110.2 (m, 2F).

HRMS (EI): Calcd. for $C_{21}H_{22}F_2O_5$ $[M+K]^+$ 431.1067, found: 431.1070.

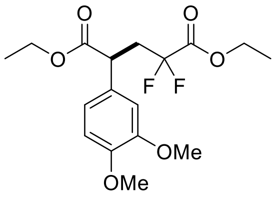

Diethyl 4-(3, 4-dimethoxyphenyl)-2, 2-difluoropentanedioate (**3ha**)

The title compound was prepared from 1, 2-dimethoxy-4-vinylbenzene (49.2 mg, 0.3 mmol) and ethyl bromodifluoroacetate (181.7 mg, 0.9 mmol) according to general procedure. The crude residue was purified by flash chromatography (PE/EA = 50:1 to 20:1) to give the product as a colorless oil (82.1 mg, 76%).

^{1}H NMR (400 MHz, $CDCl_3$) δ 6.85 – 6.75 (m, 3H), 4.23 – 4.01 (m, 4H), 3.85 (s, 3H), 3.84 – 3.78 (m, 4H), 3.09 – 2.90 (m, 1H), 2.55 – 2.36 (m, 1H), 1.27 (t, *J* = 7.1 Hz, 3H), 1.18 (t, *J* = 7.1 Hz, 3H).

^{13}C NMR (101 MHz, $CDCl_3$) δ 172.4, 163.6 (t, *J* = 32.4 Hz), 149.0, 148.5, 129.9, 119.9, 114.9 (t, *J* = 251.1 Hz), 111.2, 110.7, 62.9, 61.2, 55.8, 55.8, 44.4 (t, *J* = 4.2 Hz), 38.0 (t, *J* = 23.4 Hz), 13.9, 13.7.

^{19}F NMR (56 MHz, $CDCl_3$) δ -109.9 – -110.2 (m, 2F).

HRMS (EI): Calcd. for $C_{17}H_{22}F_2O_6$ $[M+NH_4]^+$ 378.1723 found: 378.1728

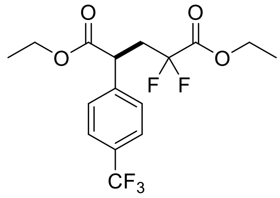

Diethyl 2,2-difluoro-4-(4-(trifluoromethyl)phenyl)pentanedioate (**3ia**)

The title compound was prepared from 1-(trifluoromethyl)-4-vinylbenzene (51.6 mg, 0.3 mmol) and ethyl bromodifluoroacetate (181.7 mg, 0.9 mmol) according to general procedure. The crude residue was purified by flash chromatography (PE/EA = 50:1 to 20:1) to give the product as a colorless oil (60.7 mg, 55%).

^{1}H NMR (400 MHz, CDCl$_3$) δ 7.59 (d, *J* = 8.2 Hz, 2H), 7.43 (d, *J* = 8.1 Hz, 2H), 4.26 – 4.04 (m, 4H), 3.98 (dd, *J* = 8.2, 5.5 Hz, 1H), 3.14 – 2.98 (m, 1H), 2.57 – 2.37 (m, 1H), 1.30 (t, *J* = 7.1 Hz, 3H), 1.20 (t, *J* = 7.1 Hz, 3H).

^{13}C NMR (101 MHz, CDCl$_3$) δ 171.7, 163.5 (t, *J* = 32.4 Hz), 141.6, 130.1 (q, *J* = 32.7 Hz), 128.3, 125.8 (q, *J* = 3.7 Hz), 123.9 (q, *J* = 272.3 Hz), 114.8 (t, *J* = 251.4 Hz), 63.1, 61.7, 44.8 (t, *J* = 4.0 Hz), 37.7 (t, *J* = 23.5 Hz), 13.9, 13.8.

^{19}F NMR (56 MHz, CDCl$_3$) δ -67.9 (s, 3F), -110.2 (t, *J* = 16.5 Hz, 2F).

HRMS (EI): Calcd. for $C_{16}H_{17}F_5O_4$ $[M+Na]^+$ 391.0939, found: 391.0933.

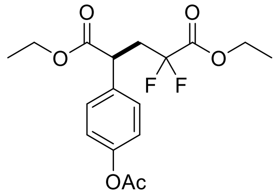

Diethyl 4-(4-acetoxyphenyl)-2,2-difluoropentanedioate (**3ja**)

The title compound was prepared from 4-vinylphenyl acetate (48.6 mg, 0.3 mmol) and ethyl bromodifluoroacetate (181.7 mg, 0.9 mmol) according to general procedure. The crude residue was purified by flash chromatography (PE/EA = 50:1 to 20:1) to give the product as a colorless oil (82.7 mg, 77%).

^{1}H NMR (400 MHz, CDCl$_3$) δ 7.30 (d, *J* = 8.5 Hz, 2H), 7.04 (d, *J* = 8.5 Hz, 2H), 4.23 – 4.01 (m, 4H), 3.89 (dd, *J* = 8.5, 5.2 Hz, 1H), 3.10 – 2.94 (m, 1H), 2.56 – 2.38 (m, 1H), 2.27 (s, 3H), 1.28 (t, *J* = 7.1 Hz, 3H), 1.19 (t, *J* = 7.1 Hz, 3H).

^{13}C NMR (101 MHz, CDCl$_3$) δ 172.1, 169.3, 163.5 (t, *J* = 32.4 Hz), 150.2, 134.9, 128.8, 121.9, 114.8 (t, *J* = 251.1 Hz), 63.0, 61.4, 44.3 (t, *J* = 42.0 Hz), 37.9 (t, *J* = 23.4 Hz), 21.0, 13.9, 13.7.

^{19}F NMR (56 MHz, CDCl$_3$) δ -109.6 – -110.6 (m, 2F).

HRMS (EI): Calcd. for $C_{17}H_{20}F_2O_6$ $[M+NH_4]^+$ 376.1566, found: 376.1562.

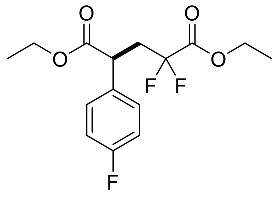

Diethyl 2,2-difluoro-4-(4-fluorophenyl)pentanedioate (**3ka**)

The title compound was prepared from 1-fluoro-4-vinylbenzene (36.6 mg, 0.3 mmol) and ethyl

bromodifluoroacetate (181.7 mg, 0.9 mmol) according to general procedure. The crude residue was purified by flash chromatography (PE/EA = 50:1 to 20:1) to give the product as a colorless oil (62.0 mg, 65%).

^{1}H NMR (400 MHz, $CDCl_3$) δ 7.30 – 7.25 (m, 2H), 7.05 – 6.99 (m, 2H), 4.26 – 4.04 (m, 4H), 3.90 (dd, *J* = 8.4, 5.5 Hz, 1H), 3.11 – 2.94 (m, 1H), 2.55 – 2.39 (m, 1H), 1.31 (t, *J* = 7.2 Hz, 3H), 1.20 (t, *J* = 7.1 Hz, 3H).

^{13}C NMR (101 MHz, $CDCl_3$) δ 172.2, 163.5 (t, *J* = 32.4 Hz), 162.3 (d, *J* = 246.7 Hz), 133.3 (d, *J* = 3.3 Hz), 129.4 (d, *J* = 8.1 Hz), 115.7 (d, *J* = 21.6 Hz), 114.9 (t, *J* = 251.3 Hz), 63.0, 61.4, 44.1 (t, *J* = 4.2 Hz), 37.9 (t, *J* = 23.5 Hz), 13.9, 13.8.

^{19}F NMR (56 MHz, $CDCl_3$) δ -110.1 (dd, *J* = 16.4, 5.5 Hz, 1F), -110.4 (dd, *J* = 16.2, 5.8 Hz, 1F), -119.2 – -120.0 (m, 1F).

HRMS (EI): Calcd. for $C_{15}H_{17}F_3O_4$ $[M+H]^+$ 341.0971, found: 341.0965.

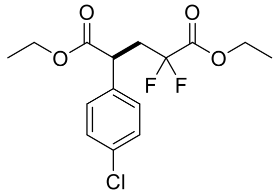

Diethyl 4-(4-chlorophenyl)-2,2-difluoropentanedioate (**3la**)

The title compound was prepared from 1-chloro-4-vinylbenzene (41.4 mg, 0.3 mmol) and ethyl bromodifluoroacetate (181.7 mg, 0.9 mmol) according to general procedure. The crude residue was purified by flash chromatography (PE/EA = 50:1 to 20:1) to give the product as a colorless oil (68.1 mg, 68%).

^{1}H NMR (400 MHz, $CDCl_3$) δ 7.30 (d, *J* = 8.5 Hz, 2H), 7.23 (d, *J* = 8.5 Hz, 2H), 4.26 – 4.03 (m, 4H), 3.88 (dd, *J* = 8.3, 5.5 Hz, 1H), 3.11 – 2.93 (m, 1H), 2.54 – 2.37 (m, 1H), 1.30 (t, *J* = 7.2 Hz, 3H), 1.19 (t, *J* = 7.1 Hz, 3H).

^{13}C NMR (101 MHz, $CDCl_3$) δ 172.0, 163.5 (t, *J* = 32.3 Hz), 136.1, 133.7, 129.1, 129.0, 114.8 (t, *J* = 251.3 Hz), 63.0, 61.5, 44.3 (t, *J* = 4.1 Hz), 37.7 (t, *J* = 23.5 Hz), 13.9, 13.8.

^{19}F NMR (56 MHz, $CDCl_3$) δ -109.9 – -110.2 (m, 2F).

HRMS (EI): Calcd. for $C_{15}H_{17}ClF_2O_4$ $[M+Na]^+$ 357.0676, found: 357.0664.

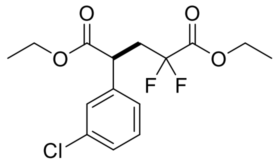

Diethyl 4-(3-chlorophenyl)-2,2-difluoropentanedioate (**3ma**)

The title compound was prepared from 1-chloro-3-vinylbenzene (41.4 mg, 0.3 mmol) and ethyl bromodifluoroacetate (181.7 mg, 0.9 mmol) according to general procedure. The crude residue was purified by flash chromatography (PE/EA = 50:1 to 20:1) to give the product as a colorless oil (66.2 mg, 66%).

^{1}H NMR (400 MHz, $CDCl_3$) δ 7.35 – 7.26 (m, 3H), 7.24 – 7.19 (m, 1H), 4.29 – 4.07 (m, 4H), 3.90 (dd, *J* = 7.9, 5.7 Hz, 1H), 3.15 – 2.92 (m, 1H), 2.57 – 2.40 (m, 1H), 1.33 (t, *J* = 7.1 Hz, 3H), 1.23 (t, *J* = 7.1 Hz, 3H).

^{13}**C NMR (101 MHz, CDCl$_3$)** δ 171.8, 163.5 (t, *J* = 32.3 Hz), 139.5, 134.7, 130.1, 128.1, 128.0, 126.1, 114.8 (t, *J* = 251.3 Hz), 63.1, 61.6, 44.6 (t, *J* = 4.1 Hz), 37.8 (t, *J* = 23.5 Hz) 13.9, 13.8.

^{19}**F NMR (56 MHz, CDCl$_3$)** δ -109.9– -110.0 (m, 2F).

HRMS (EI): Calcd. for $C_{15}H_{17}ClF_2O_4$ $[M+NH_4]^+$ 352.1122, found: 352.1109.

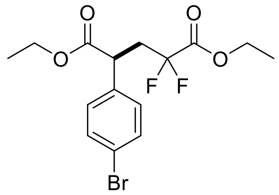

Diethyl 4-(4-bromophenyl)-2,2-difluoropentanedioate (**3na**)

The title compound was prepared from 1-bromo-4-vinylbenzene (54.6 mg, 0.3 mmol) and ethyl bromodifluoroacetate (181.7 mg, 0.9 mmol) according to general procedure. The crude residue was purified by flash chromatography (PE/EA = 50:1 to 20:1) to give the product as a colorless oil (68.0 mg, 60%).

^{1}**H NMR (400 MHz, CDCl$_3$)** δ 7.45 (d, *J* = 8.4 Hz, 2H), 7.17 (d, *J* = 8.4 Hz, 2H), 4.25 – 4.03 (m, 4H), 3.87 (dd, *J* = 8.3, 5.5 Hz, 1H), 3.10 – 2.93 (m, 1H), 2.53 – 2.38 (m, 1H), 1.30 (t, *J* = 7.2 Hz, 3H), 1.19 (t, *J* = 7.1 Hz, 3H).

^{13}**C NMR (101 MHz, CDCl$_3$)** δ 171.9, 163.5 (t, *J* = 32.3 Hz), 136.6, 131.9, 129.5, 121.8, 114.8 (t, *J* = 251.4 Hz), 63.0, 61.5, 44.4 (t, *J* = 4.1 Hz), 37.7 (t, *J* = 23.5 Hz), 13.9, 13.8.

^{19}**F NMR (56 MHz, CDCl$_3$)** δ -109.7 – -110.0 (m, 2F).

HRMS (EI): Calcd. for $C_{15}H_{17}BrF_2O_4$ $[M+NH_4]^+$ 396.0617, found: 396.0611.

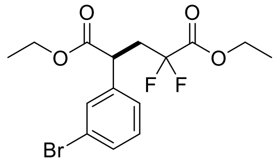

Diethyl 4-(3-bromophenyl)-2,2-difluoropentanedioate (**3oa**)

The title compound was prepared from 1-bromo-3-vinylbenzene (54.6 mg, 0.3 mmol) and ethyl bromodifluoroacetate (181.7 mg, 0.9 mmol) according to general procedure. The crude residue was purified by flash chromatography (181.8 mg, 0.9 mmol) to give the product as a colorless oil (51 mg, 45%).

^{1}**H NMR (400 MHz, CDCl$_3$)** δ 7.45 (s, 1H), 7.42 (d, *J* = 7.5 Hz, 1H), 7.25 – 7.17 (m, 2H), 4.27 – 4.04 (m, 4H), 3.87 (dd, *J* = 8.1, 5.5 Hz, 1H), 3.11 – 2.94 (m, 1H), 2.54 – 2.39 (m, 1H), 1.31 (t, *J* = 7.1 Hz, 3H), 1.20 (t, *J* = 7.1 Hz, 3H).

^{13}**C NMR (101 MHz, CDCl$_3$)** δ 171.8, 163.5 (t, *J* = 32.3 Hz), 139.8, 131.0, 131.0, 130.4, 126.6, 122.8, 114.8 (t, *J* = 251.4 Hz), 63.1, 61.6, 44.6 (t, *J* = 4.0 Hz), 37.8 (t, *J* = 23.6 Hz), 14.0, 13.9.

^{19}**F NMR (56 MHz, CDCl$_3$)** δ -109.9– -110.2 (m, 2F).

HRMS (EI): Calcd. for $C_{15}H_{17}BrF_2O_4$ $[M+NH_4]^+$ 396.0617, found: 396.0606.

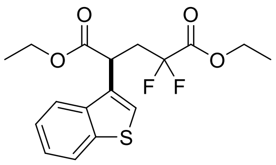

Diethyl 4-(benzo[b]thiophen-3-yl)-2,2-difluoropentanedioate (**3pa**)

The title compound was prepared from 3-vinylbenzo[b]thiophene (48 mg, 0.3 mmol) and ethyl bromodifluoroacetate (181.7 mg, 0.9 mmol) according to general procedure. The crude residue was purified by flash chromatography (PE/EA = 50:1 to 20:1) to give the product as a colorless oil (21.4 mg, 20%).

^{1}H NMR (400 MHz, CDCl$_3$) δ 7.86 (d, J = 7.8 Hz, 2H), 7.43 (t, J = 7.4 Hz, 1H), 7.38 (t, J = 7.5 Hz, 1H), 7.34 (s, 1H), 4.38 (dd, J = 8.8, 4.6 Hz, 1H), 4.26 – 4.07 (m, 4H), 3.24 – 3.04 (m, 1H), 2.70 – 2.54 (m, 1H), 1.27 – 1.17 (m, 6H).

^{13}C NMR (101 MHz, CDCl$_3$) δ 171.8, 163.5 (t, J = 32.4 Hz), 140.4, 137.3, 131.6, 124.7, 124.4, 124.0, 123.0, 121.7, 115.0 (t, J = 251.3 Hz), 63.1, 61.6, 38.8 (t, J = 4.5 Hz), 37.1 (t, J = 23.5 Hz), 14.0, 13.8.

^{19}F NMR (56 MHz, CDCl$_3$) δ -108.9 – -109.4 (m, 2F).

HRMS (EI): Calcd. for $C_{17}H_{18}F_2O_4S$ [M+ NH$_4$]$^+$ 374.1232, found: 374.1221.

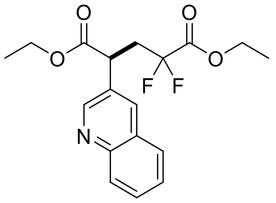

Diethyl 2,2-difluoro-4-(quinolin-3-yl)pentanedioate (**3qa**)

The title compound was prepared from 3-vinylquinoline (46.5 mg, 0.3 mmol) and ethyl bromodifluoroacetate (181.7 mg, 0.9 mmol) according to general procedure. The crude residue was purified by flash chromatography (PE/EA = 50:1 to 20:1) to give the product as a colorless oil (35.8 mg, 34%).

^{1}H NMR (400 MHz, CDCl$_3$) δ 8.87 (s, 1H), 8.10 (d, J = 8.7 Hz, 2H), 7.81 (d, J = 8.1 Hz, 1H), 7.72 (t, J = 7.6 Hz, 1H), 7.56 (t, J = 7.5 Hz, 1H), 4.25 – 4.05 (m, 5H), 2.71 – 3.08 (m, 1H), 3.28 – 2.54 (m, 1H), 1.27 (t, J = 7.1 Hz, 3H), 1.20 (t, J = 7.1 Hz, 3H).

^{13}C NMR (101 MHz, CDCl$_3$) δ 171.7, 163.5 (t, J = 32.4 Hz), 150.4, 147.6, 134.5, 130.5, 129.8, 129.3, 127.8, 127.7, 127.2, 114.8 (t, J = 251.7 Hz), 63.2, 61.9, 42.7 (t, J = 4.0 Hz), 37.7 (t, J = 23.5 Hz), 14.0, 13.8.

^{19}F NMR (56 MHz, CDCl$_3$) δ -108.7 – -109.0 (m, 2F).

HRMS (EI): Calcd. for $C_{18}H_{19}F_2NO_4$ [M+NH$_4$]$^+$ 369.1620, found: 369.1623.

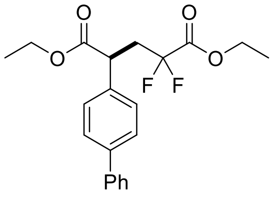

Diethyl 4-([1,1'-biphenyl]-4-yl)-2,2-difluoropentanedioate (**3ra**)

The title compound was prepared from 4-vinyl-1, 1'-biphenyl (54.0 mg, 0.3 mmol) and ethyl bromodifluoroacetate (181.7 mg, 0.9 mmol) according to general procedure. The crude residue was

purified by flash chromatography (PE/EA = 50:1 to 20:1) to give the product as a colorless oil (80.1 mg, 71%).

^{1}H NMR (400 MHz, $CDCl_3$) δ 7.61 – 7.55 (m, 4H), 7.45 (t, *J* = 7.5 Hz, 2H), 7.41 – 7.33 (m, 3H), 4.26 – 4.07 (m, 4H), 3.98 (dd, *J* = 8.5, 5.2 Hz, 1H), 3.20 – 3.03 (m, 1H), 2.64 – 2.48 (m, 1H), 1.31 (t, *J* = 7.2 Hz, 3H), 1.23 (t, *J* = 7.1 Hz, 3H).

^{13}C NMR (101 MHz, $CDCl_3$) δ 172.3, 163.6 (t, *J* = 32.4 Hz), 140.7, 140.4, 136.5, 128.8, 128.2, 127.5, 127.4, 127.0, 115.0 (t, *J* = 251.1 Hz), 62.9, 61.4, 44.5 (t, *J* = 4.2 Hz), 37.9 (t, *J* = 23.5 Hz), 13.9, 13.8.

^{19}F NMR (56 MHz, $CDCl_3$) δ -109.8– -110.1 (m, 2F).

HRMS (EI): Calcd. for $C_{21}H_{22}F_2O_4$ $[M+H]^+$ 377.1559, found: 377.1553.

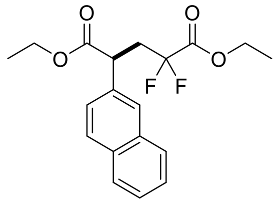

Diethyl 2,2-difluoro-4-(naphthalen-2-yl)pentanedioate (**3sa**)

The title compound was prepared from 2-vinylnaphthalene (46.2 mg, 0.3 mmol) and ethyl bromodifluoroacetate (181.7 mg, 0.9 mmol) according to general procedure. The crude residue was purified by flash chromatography (PE/EA = 50:1 to 20:1) to give the product as a colorless oil (79.8 mg, 76%).

^{1}H NMR (400 MHz, $CDCl_3$) δ 7.85 – 7.80 (m, 3H), 7.77 (s, 1H), 7.52 – 7.47 (m, 2H), 7.46 – 7.41 (m, 1H), 4.25 – 4.17 (m, 1H), 4.16 – 4.04 (m, 4H), 3.26 – 3.09 (m, 1H), 2.71 – 2.54 (m, 1H), 1.25 (t, *J* = 7.2 Hz, 3H), 1.20 (t, *J* = 7.1 Hz, 3H).

^{13}C NMR (101 MHz, $CDCl_3$) δ 172.3, 163.6 (t, *J* = 32.4 Hz), 134.9, 133.3, 132.7, 128.6, 127.8, 127.6, 126.8, 126.4, 126.2, 125.4, 115.0 (t, *J* = 251.1 Hz), 62.9, 61.4, 45.0 (t, *J* = 4.2 Hz), 37.9 (t, *J* = 23.5 Hz), 13.9, 13.7.

^{19}F NMR (56 MHz, $CDCl_3$) δ -109.7– -110.0 (m, 2F).

HRMS (EI): Calcd. for $C_{19}H_{20}F_2O_4$ $[M+Na]^+$ 373.1222, found: 373.1215.

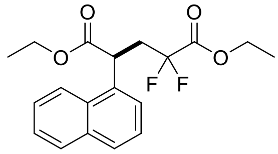

Diethyl 2,2-difluoro-4-(naphthalen-1-yl) pentanedioate (**3ta**)

The title compound was prepared from 1-vinylnaphthalene (46.2 mg, 0.3 mmol) and ethyl bromodifluoroacetate (181.7 mg, 0.9 mmol) according to general procedure. The crude residue was purified by flash chromatography (PE/EA = 50:1 to 20:1) to give the product as a colorless oil (31.5 mg, 30%).

^{1}H NMR (400 MHz, $CDCl_3$) δ 8.13 (d, *J* = 8.5 Hz, 1H), 7.88 (d, *J* = 8.0 Hz, 1H), 7.80 (dd, *J* = 6.2, 3.0 Hz, 1H), 7.59 (t, *J* = 7.1 Hz, 1H), 7.52 (t, *J* = 7.5 Hz, 1H), 7.47 – 7.40 (m, 2H), 4.74 (dd, *J* = 8.8, 4.1 Hz, 1H), 4.25 – 4.04 (m, 4H), 3.26 (ddd, *J* = 31.9, 16.2, 9.0 Hz, 1H), 2.57 (ddd, *J* = 32.0, 16.8, 4.2 Hz, 1H), 1.24 (t, *J* = 7.2 Hz, 3H), 1.16 (t, *J* = 7.1 Hz, 3H).

^{13}C NMR (101 MHz, $CDCl_3$) δ 172.7, 163.6 (t, *J* = 32.5 Hz), 134.1, 133.9, 130.8, 129.0, 128.4, 126.7, 125.9, 125.4, 125.2, 122.9, 115.1 (t, *J* = 251.1 Hz), 63.0, 61.5, 40.5, 37.8 (t, *J* = 23.3 Hz), 13.9, 13.7.

^{19}F NMR (56 MHz, CDCl3) δ -110.0 – -110.3 (m, 2F).
HRMS (EI): Calcd. for $C_{19}H_{20}F_2O_4$ $[M+H]^+$ 373.1222, found: 373.1214.

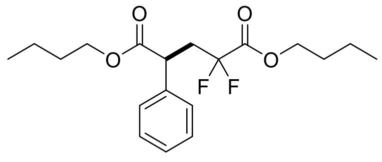

Dibutyl 2, 2-difluoro-4-phenylpentanedioate (**3ab**)
The title compound was prepared from styrene (31.2 mg, 0.3 mmol) and butyl 2-bromo-2, 2-difluoroacetate (207.0 mg, 0.9 mmol) according to general procedure. The crude residue was purified by flash chromatography (PE/EA = 50:1 to 20:1) to give the product as a colorless oil (72.7 mg, 68%).
^{1}H NMR (400 MHz, CDCl$_3$) δ 7.35 – 7.26 (m, 5H), 4.18 – 3.99 (m, 4H), 3.90 (dd, J = 8.3, 5.3 Hz, 1H), 3.20 – 2.97 (m, 1H), 2.59 – 2.41 (m, 1H), 1.68 – 1.60 (m, 2H), 1.59 – 1.49 (m, 2H), 1.43 – 1.33 (m, 2H), 1.30 – 1.20 (m, 2H), 0.94 (t, J = 7.4 Hz, 3H), 0.85 (t, J = 7.4 Hz, 3H).
^{13}C NMR (101 MHz, CDCl$_3$) δ 172.4, 163.7 (t, J = 32.4 Hz), 137.7, 128.8, 127.8, 115.1 (t, J = 251.1 Hz), 66.7, 65.2, 44.9 (t, J = 3.7 Hz), 37.8 (t, J = 23.4 Hz), 30.4, 30.2, 18.9, 13.6, 13.6.
^{19}F NMR (56 MHz, CDCl$_3$) δ -108.7 – -109.0 (m, 2F).
HRMS (EI): Calcd. for $C_{19}H_{26}F_2O_4$ $[M+NH_4]^+$ 374.2137, found: 374.2142.

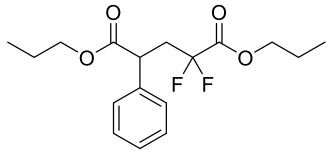

Dipropyl 2,2-difluoro-4-phenylpentanedioate (**3ac**)
The title compound was prepared from styrene (31.2 mg, 0.3 mmol) and propyl 2-bromo-2,2-difluoroacetate (194.4 mg, 0.9 mmol) according to general procedure. The crude residue was purified by flash chromatography (PE/EA = 50:1 to 20:1) to give the product as a colorless oil 52.2 mg, 53%).
^{1}H NMR (700 MHz, CDCl$_3$) δ 7.33 – 7.25 (m, 5H), 4.11 – 4.07 (m, 1H), 4.07 – 4.02 (m, 2H), 4.02 – 3.92 (m, 1H), 3.94 – 3.90 (m, 1H), 3.12 – 3.02 (m, 1H), 2.56 – 2.45 (m, 1H), 1.73 – 1.65 (m, 2H), 1.62 – 1.55 (m, 2H), 0.95 (t, J = 7.5 Hz, 3H), 0.83 (t, J = 7.5 Hz, 3H).
^{13}C NMR (176 MHz, CDCl$_3$) δ 172.4, 163.7 (t, J = 32.4 Hz), 137.6, 128.8, 127.7, 127.7, 115.0 (t, J = 251.1 Hz), 68.3, 66.9, 44.9 (t, J = 4.0 Hz), 37.8 (t, J = 23.4 Hz), 21.7, 21.6, 10.1, 10.1.
^{19}F NMR (56 MHz, CDCl$_3$) δ -104.3 – -105.0 (m, 2F).
HRMS (ESI-TOF) m/z: $[M+H]^+$ Calcd. for 329.1559, found: 329.1561.

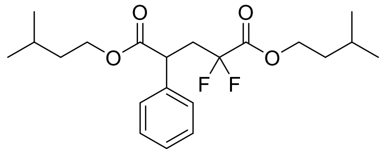

Diisopentyl 2,2-difluoro-4-phenylpentanedioate (**3ad**)
The title compound was prepared from styrene (31.2 mg, 0.3 mmol) and isopentyl 2-bromo-2,2-difluoroacetate (219.6 mg, 0.9 mmol) according to general procedure. The crude residue was purified by flash chromatography (PE/EA = 50:1 to 20:1) to give the product as a colorless oil

(93.4 mg, 81%).

^{1}H NMR (700 MHz, $CDCl_3$) δ 7.28 – 7.23 (m, 2H), 7.23 – 7.18 (m, 3H), 4.12 – 3.95 (m, 4H), 3.82 (dd, J = 8.0, 5.1 Hz, 1H), 3.04 – 2.92 (m, 1H), 2.47 – 2.42 (m, 1H), 1.64 – 1.54 (m, 1H), 1.53 –1.34 (m, 3H), 1.40 –1.34 (m, 2H), 0.85 (d, J = 6.6 Hz, 6H), 0.79 –0.73 (m, 6H).

^{13}C NMR (176 MHz, $CDCl_3$) δ 172.4, 163.7 (t, J = 32.3 Hz), 137.6, 128.8, 127.8, 115.0 (t, J = 251.2 Hz), 65.5 64.0, 44.9 (t, J = 3.9 Hz), 37.8 (t, J = 23.4 Hz), 37.0, 36.8, 24.9, 24.9, 22.3, 22.3, 22.3, 22.3.

^{19}F NMR (56 MHz, $CDCl_3$) δ -104.3 – -105.0 (m, 2F).

HRMS (ESI-TOF) m/z: $[M+H]^+$ Calcd. for 385.2185, found: 385.2181.

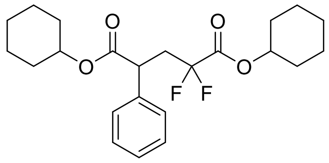

Dicyclohexyl 2,2-difluoro-4-phenylpentanedioate (with dicyclohexyl oxalate from substrate) (**3ae**)

Dicyclohexyl 2,2-difluoro-4-phenylpentanedioate: dicyclohexyl oxalate = 5:2

The title compound was prepared from styrene (31.2 mg, 0.3 mmol) and cyclohexyl 2-bromo-2,2-difluoroacetate (230.4 mg, 0.9 mmol) according to general procedure. The crude residue was purified by flash chromatography (PE/EA = 50:1 to 20:1) to give the product as a colorless oil (a mixture of dicyclohexyl 2,2-difluoro-4-phenylpentanedioate: dicyclohexyl oxalate = 5:2, 55.1 mg, the calculated yield is approximately 36%).

^{1}H NMR (700 MHz, $CDCl_3$) δ 7.27 – 7.22 (m, 4H), 7.21 – 7.18 (m, 1H), 4.87 – 4.82 (m, 1H), 4.75 – 4.70 (m, 1H), 4.70 – 4.65 (m, 1H), 3.82 (dd, J = 8.8, 4.8 Hz, 1H), 3.04 – 2.94 (m, 1H), 2.43 – 2.34 (m, 1H), 1.87 – 1.84 (m, 1H), 1.81 – 1.77 (m, 1H), 1.77 –1.72 (m, 2H), 1.72 – 1.68 (m, 2H), 1.68 –1.63 (m, 2H), 1.62 – 1.56 (m, 2H), 1.52 – 1.44 (m, 5H), 1.43 – 1.36 (m, 3H), 1.35 – 1.25 (m, 5H), 1.25 – 1.15 (m, 5H).

^{13}C NMR (176 MHz, $CDCl_3$) δ 171.8, 163.1 (t, J = 32.2 Hz), 157.8, 138.0, 128.8, 127.7, 127.6, 115.1 (t, J = 251.3 Hz), 75.9, 75.9, 73.4, 45.1 (t, J =3.6 Hz), 37.8 (t, J =23.2 Hz), 31.2, 31.2, 31.0, 31.0, 25.3, 25.1, 25.1, 23.6, 23.4, 23.4, 23.3.

^{19}F NMR (56 MHz, $CDCl_3$) δ -104.5 – -105.3 (m, 2F).

HRMS (ESI-TOF) m/z: $[M+H]^+$ Calcd. for 409.2185, found: 409.2185.

Dicyclohexyl oxalate: **HRMS (ESI-TOF) m/z:** $[M+NH_4]^+$ Calcd. for 272.1856, found: 272.1855.

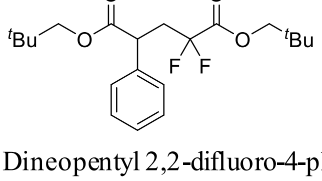

Dineopentyl 2,2-difluoro-4-phenylpentanedioate (**3af**)

The title compound was prepared from styrene (31.2 mg, 0.3 mmol) and 3-phenylpropyl 2-bromo-2,2-difluoroacetate (219.6 mg, 0.9 mmol) according to general procedure. The crude residue was purified by flash chromatography (PE/EA = 50:1 to 20:1) to give the product as a colorless oil (57.6 mg, 50%).

^{1}H NMR (700 MHz, $CDCl_3$) δ 7.35 – 7.28 (m, 4H), 7.28 – 7.24 (m, 1H), 3.98 – 3.94 (m, 1H), 3.85 (d, J = 10.4 Hz, 1H), 3.79 (t, J = 10.6 Hz, 2H), 3.72 (d, J = 10.5 Hz, 1H), 3.16 – 3.05 (m, 1H), 2.57 – 2.49 (m, 1H), 0.95 (s, 9H), 0.82 (s, 9H).

^{13}C NMR (176 MHz, CDCl$_3$) δ 172.3, 163.7 (t, J = 32.4 Hz), 137.7, 128.8, 127.8, 127.7, 115.1 (t, J = 251.2 Hz), 75.7, 74.4, 45.0 (d, J = 3.9 Hz), 37.6 (t, J = 23.3 Hz), 31.4, 31.4, 26.2, 26.2.
^{19}F NMR (56 MHz, CDCl$_3$) δ -104.1 – -104.9 (m, 2F).
HRMS (ESI-TOF) m/z: $[M+H]^+$ Calcd. for 385.2185, found: 385.2185.

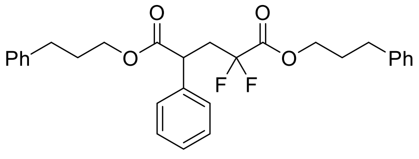

Bis(3-phenylpropyl) 2,2-difluoro-4-phenylpentanedioate (**3ag**)
The title compound was prepared from styrene (31.2 mg, 0.3 mmol) and 3-phenylpropyl 2-bromo-2,2-difluoroacetate (262.8 mg, 0.9 mmol) according to general procedure. The crude residue was purified by flash chromatography (PE/EA = 50:1 to 20:1) to give the product as a colorless oil (60.5 mg, 42%).
^{1}H NMR (700 MHz, CDCl$_3$) δ 7.34 – 7.29 (m, 4H), 7.27 (t, J = 7.2 Hz, 3H), 7.23 – 7.18 (m, 3H), 7.14 (t, J = 7.2 Hz, 3H), 7.01 (d, J = 7.5 Hz, 2H), 4.15 – 4.10 (m, 1H), 4.09 – 4.01 (m, 3H), 3.96 – 3.91 (m, 1H), 3.14 – 3.03 (m, 1H), 2.66 (t, J = 7.6 Hz, 2H), 2.56 – 2.45 (m, 3H), 2.00 – 1.94 (m, 2H), 1.89 – 1.81 (m, 2H).
^{13}C NMR (176 MHz, CDCl$_3$) δ 172.3, 163.6 (t, J = 32.5 Hz), 140.9, 140.5, 137.5, 128.9, 128.5, 128.3, 128.3, 128.3, 127.8, 127.8, 126.1, 125.9, 115.0 (t, J = 251.2 Hz), 66.0, 64.4, 44.9 (t, J = 3.9 Hz), 37.7 (t, J = 23.4 Hz), 31.7, 31.7, 30.0, 29.6.
^{19}F NMR (56 MHz, CDCl$_3$) δ -104.1 – -104.8 (m, 2F).
HRMS (ESI-TOF) m/z: $[M+H]^+$ Calcd. for 481.2185, found:481.2180.

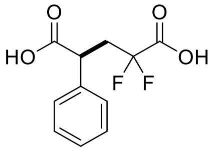

2, 2-Difluoro-4-phenylpentanedioic acid (**4**)
White solid (67.3 mg, 92%).
^{1}H NMR (400 MHz, DMSO) δ 12.62 (br, 2H), 7.47 – 7.20 (m, 5H), 3.78 (dd, J = 8.1, 4.8 Hz, 1H), 3.10 – 2.91 (m, 1H), 2.47 – 2.30 (m, 1H).
^{13}C NMR (101 MHz, DMSO) δ 173.4, 164.8 (t, J = 31.4 Hz), 138.6, 128.7, 127.7, 127.5, 115.6 (t, J = 249.5 Hz), 44.5, 37.0 (t, J = 23.0 Hz).
^{19}F NMR (56 MHz, DMSO) δ -109.7 – -110.6 (m, 2F).
HRMS (EI): Calcd. for $C_{11}H_{10}F_2O_4$ $[M+Na]^+$ 267.0439, found: 267.0429.

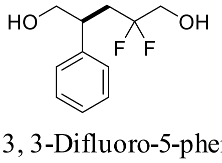

3, 3-Difluoro-5-phenyltetrahydro-2*H*-pyran (**5**)
Colorless oil (127.0 mg, 98%).

^{1}H NMR (700 MHz, CDCl$_3$) δ 7.30 (t, *J* = 7.5 Hz, 2H), 7.24 – 7.21 (m, 1H), 7.19 (d, *J* = 7.7 Hz, 2H), 3.67 (d, *J* = 3.4 Hz, 2H), 3.57 (dd, *J* = 12.6 Hz, 1H), 3.47 (dd, *J* = 25.3, 12.6 Hz, 1H), 3.11 – 3.05 (m, 1H), 2.44 – 2.35 (m, 1H) 2.27 – 2.17 (m, 1H).
^{13}C NMR (176 MHz, CDCl$_3$) δ 141.6, 128.7, 127.7, 127.0, 123.1 (t, *J* = 243.0 Hz), 66.9, 63.8 (t, *J* = 31.9 Hz), 42.0, 35.3 (t, *J* = 23.7 Hz).
^{19}F NMR (56 MHz, CDCl$_3$) δ -109.91 (t, *J* = 17.2 Hz, 2F).
HRMS (EI): Calcd. for $C_{11}H_{14}F_2O_2$ $[M+NH_4]^+$ 234.1300, found: 234.1303.

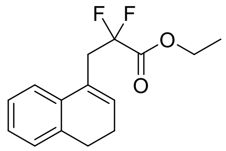

Ethyl 3-(3,4-dihydronaphthalen-1-yl)-2,2-difluoropropanoate (**8**)
The title compound was prepared from (1-cyclopropylvinyl)benzene (43.2 mg, 0.3 mmol) and ethyl bromodifluoroacetate (181.7 mg, 0.9 mmol) according to general procedure. The crude residue was purified by flash chromatography (PE/EA = 50:1 to 30:1) to give the product as a colorless oil (43.9 mg, 55%).
^{1}H NMR (700 MHz, CDCl$_3$) δ 7.16 (d, *J* = 5.4 Hz, 1H), 7.11 (t, *J* = 7.3 Hz, 1H), 7.08 – 7.03 (m, 2H), 6.00 (t, *J* = 4.4 Hz, 1H), 4.05 (q, *J* = 7.1 Hz, 2H), 3.16 (t, *J* = 15.9 Hz, 2H), 2.66 (t, *J* = 8.0 Hz, 2H), 2.21 – 2.17 (m, 2H), 1.15 – 1.10 (m, 3H).
^{13}C NMR (176 MHz, CDCl$_3$) δ 164.1 (t, *J* = 32.6 Hz), 136.3, 133.8, 131.6, 127.6, 127.3 (t, *J* = 4.3 Hz), 127.1, 126.3, 122.8, 115.5 (t, *J* = 252.3 Hz), 62.7, 37.3 (t, *J* = 24.2 Hz), 28.0, 23.2, 13.7.
^{19}F NMR (56 MHz, CDCl$_3$) δ -102.8 (t, *J* = 15.9 Hz).
HRMS (ESI-TOF) m/z: $[M+H]^+$ Calcd. for 267.1191, found: 267.1192.

8. References

1) M. C. Hsu, A. J. Junia, A. R. Haight, W. Zhang, *J. Org. Chem.* **2004**, *69*, 3907-3911.
2) T. L. Andersen, M. W. Frederiksen, K. Domino, T. Skrydstrup, *Angew. Chem. Int. Ed.* **2016**, *128*, 10396-10400.
3) M. Zhang, J.-H. Lin, C.-M. Jin, J.-C. Xiao, Chem. Commun. **2021**, 57, 2649-2652.
4) D. Kurandina, M. Parasram, V. Gevorgyan, *Angew. Chem. Int. Ed.* **2017**, *56*, 14212-14216.

9. Copy of ^{1}H and ^{13}C NMR Spectra of Substrates and Products

9.1 Substrates

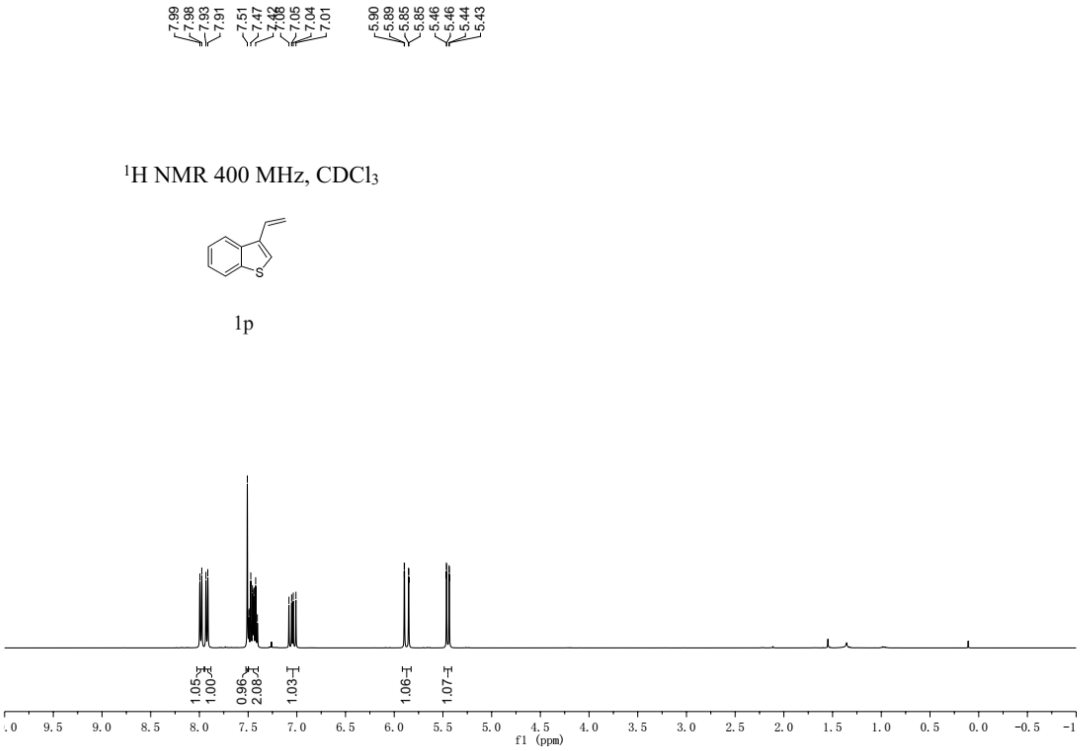

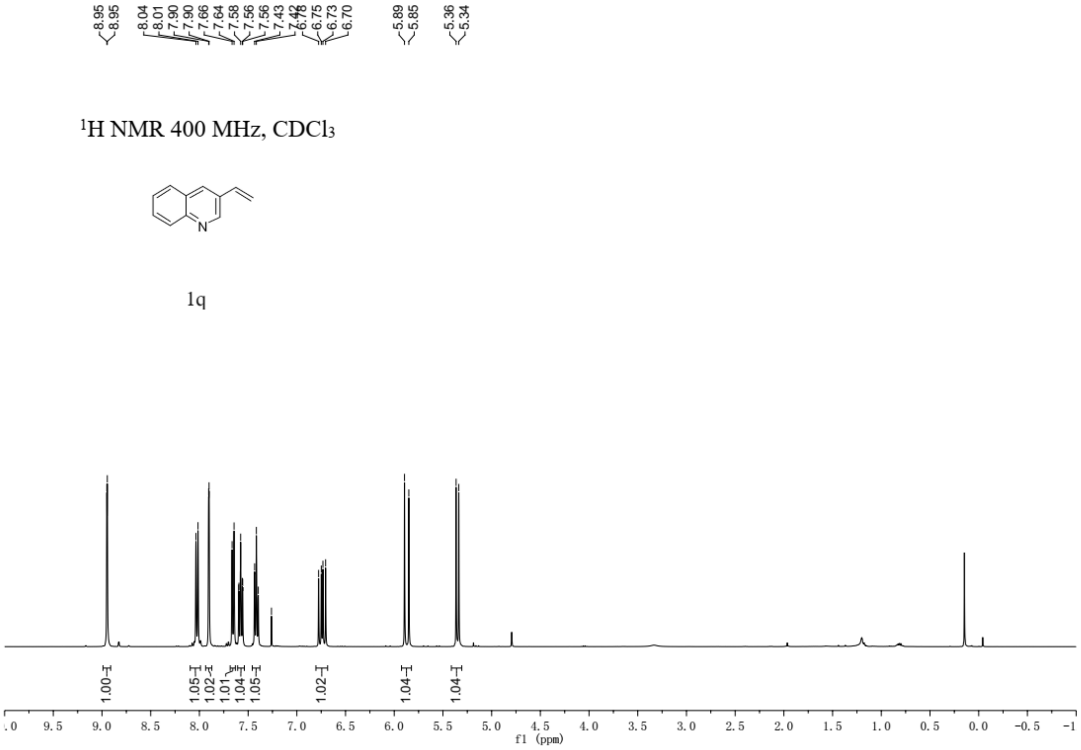

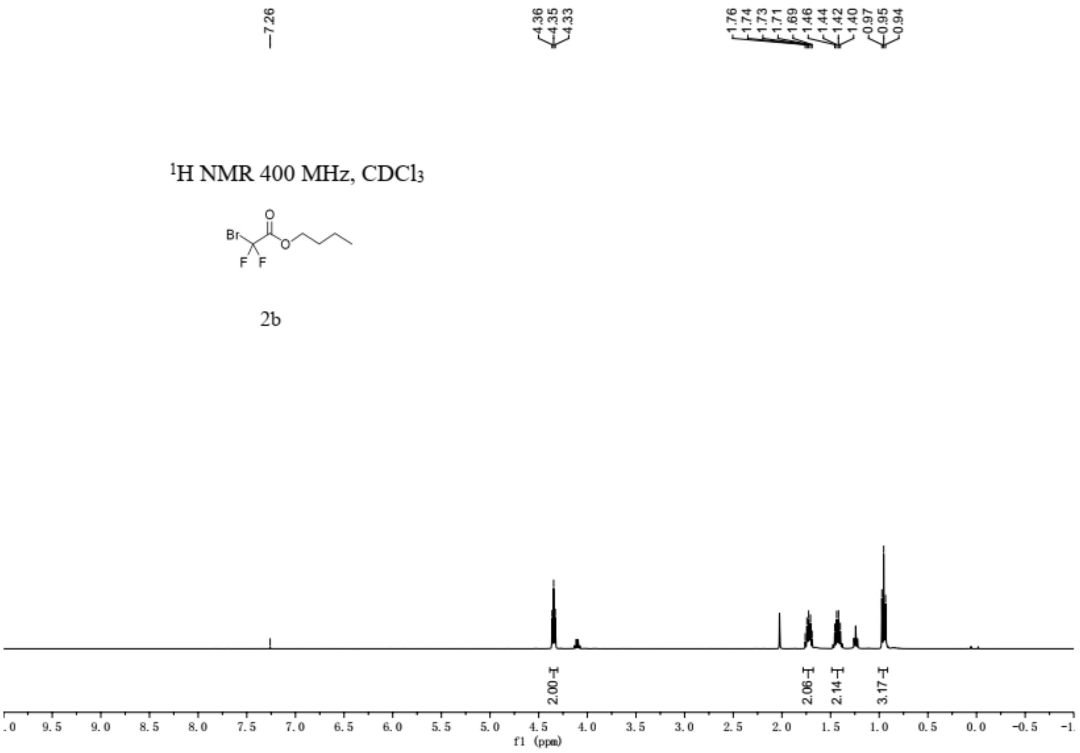

7.26
4.36
4.35
4.33
1.76
1.74
1.73
1.71
1.69
1.46
1.44
1.42
1.40
0.97
0.95
0.94
¹H NMR 400 MHz, CDCl₃
Br
O
F F
2b
2.00
2.06
2.14
3.17
f1 (ppm)

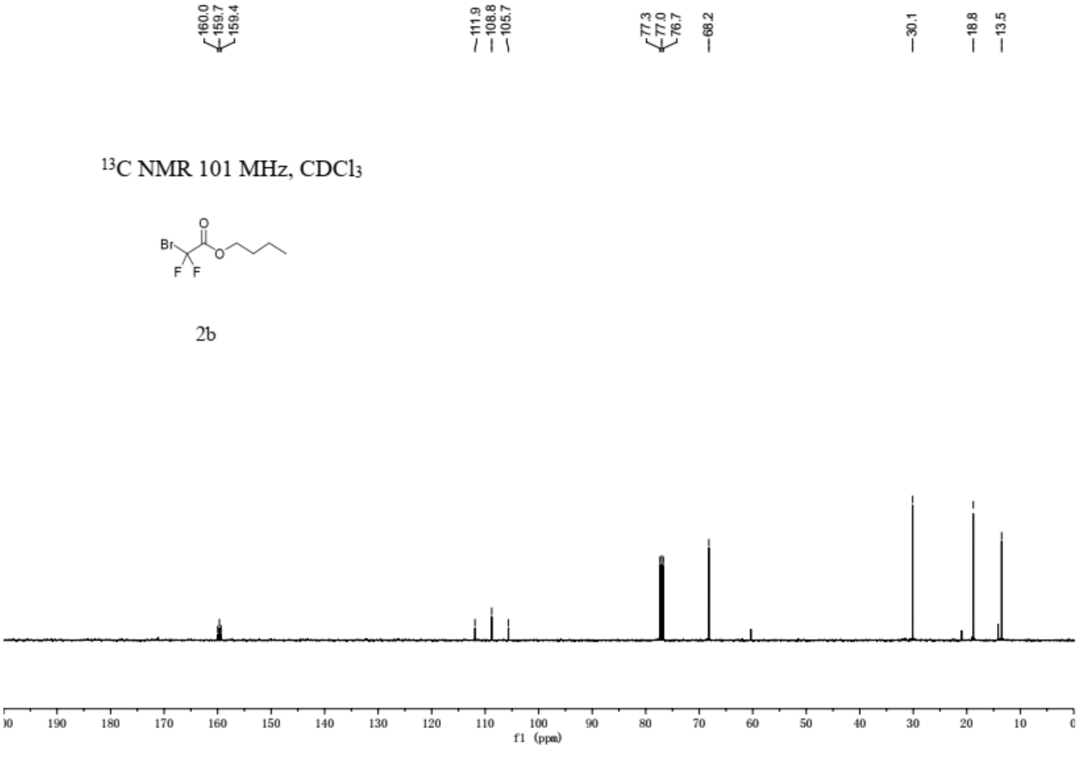

160.0
159.7
159.4
111.9
108.8
105.7
77.3
77.0
76.7
68.2
30.1
18.8
13.5
¹³C NMR 101 MHz, CDCl₃
Br
O
F F
2b
f1 (ppm)

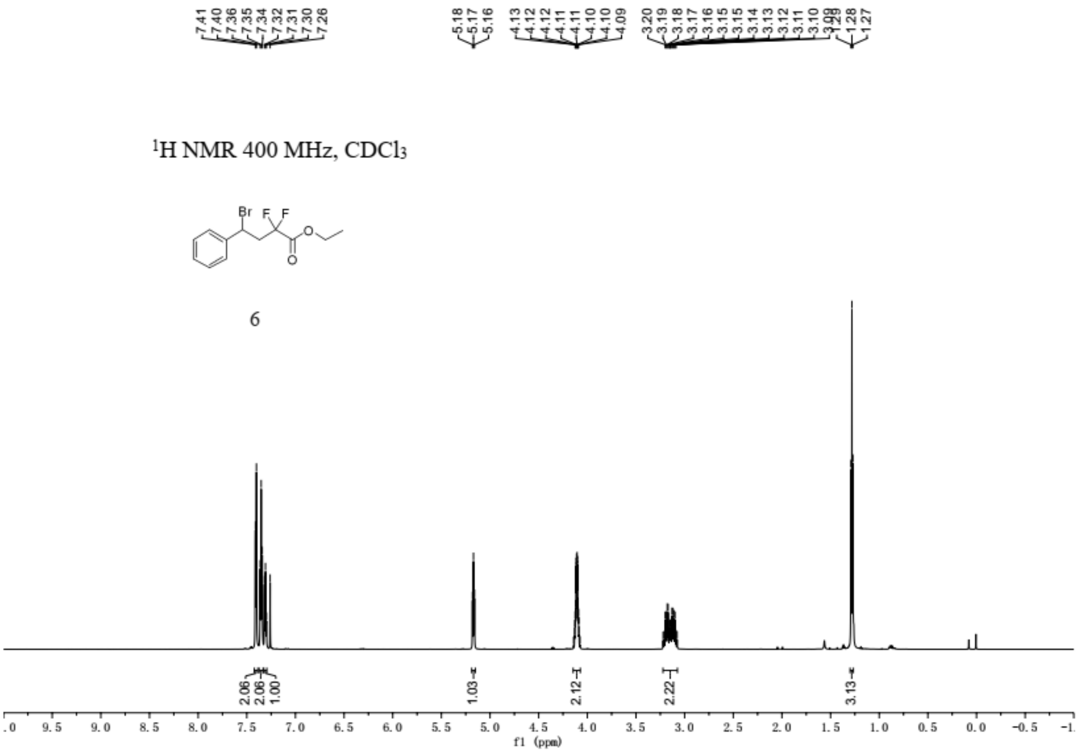
7.41
7.40
7.36
7.35
7.34
7.32
7.31
7.30
7.26
5.18
5.17
5.16
4.13
4.12
4.12
4.11
4.11
4.10
4.10
4.09
3.20
3.19
3.18
3.17
3.16
3.15
3.15
3.14
3.13
3.12
3.11
3.10
1.28
1.27
^{1}H NMR 400 MHz, $CDCl_3$
Br
F
F
O
O
6
2.06
2.08
1.00
1.03
2.12
2.22
3.13
f1 (ppm)

9.2 Products.

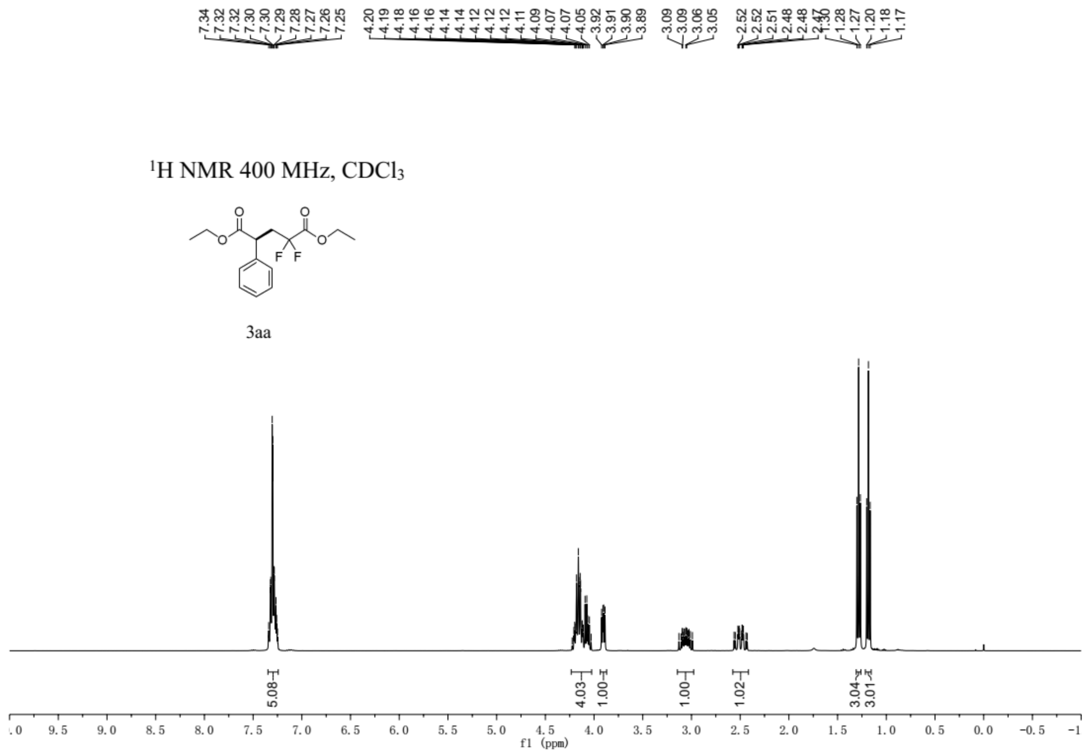

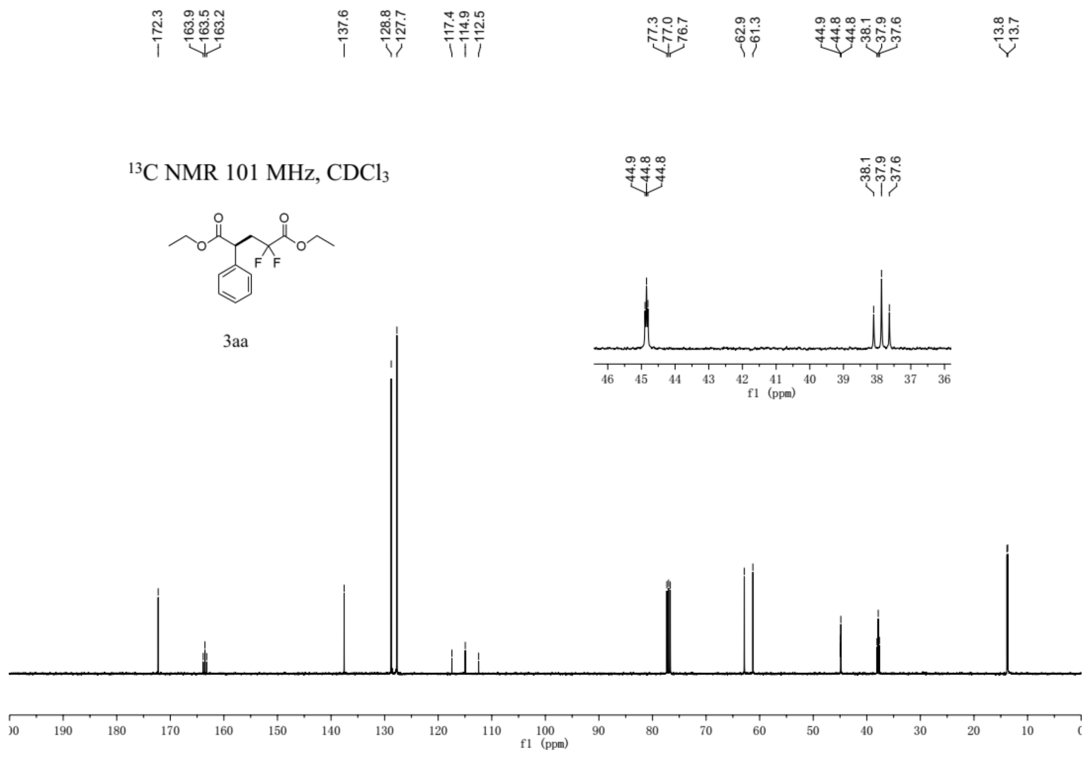

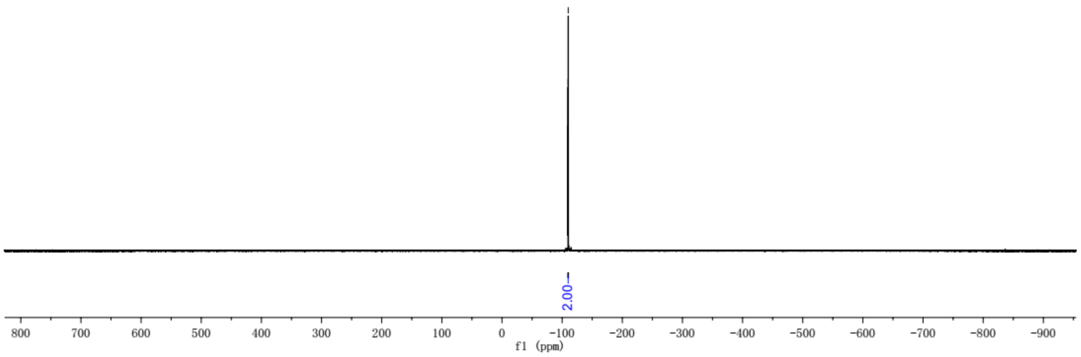

^{19}F NMR 56 MHz, $CDCl_3$

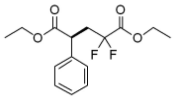

3aa

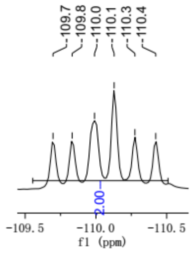

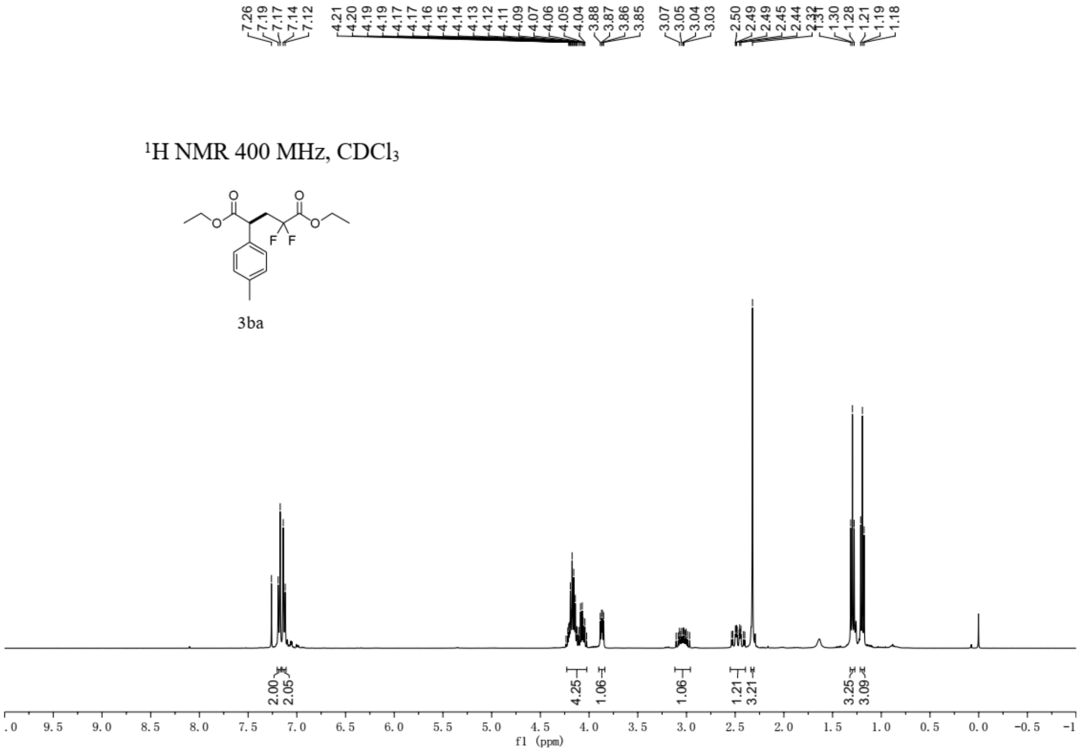
1H NMR 400 MHz, CDCl3
3ba
f1 (ppm)

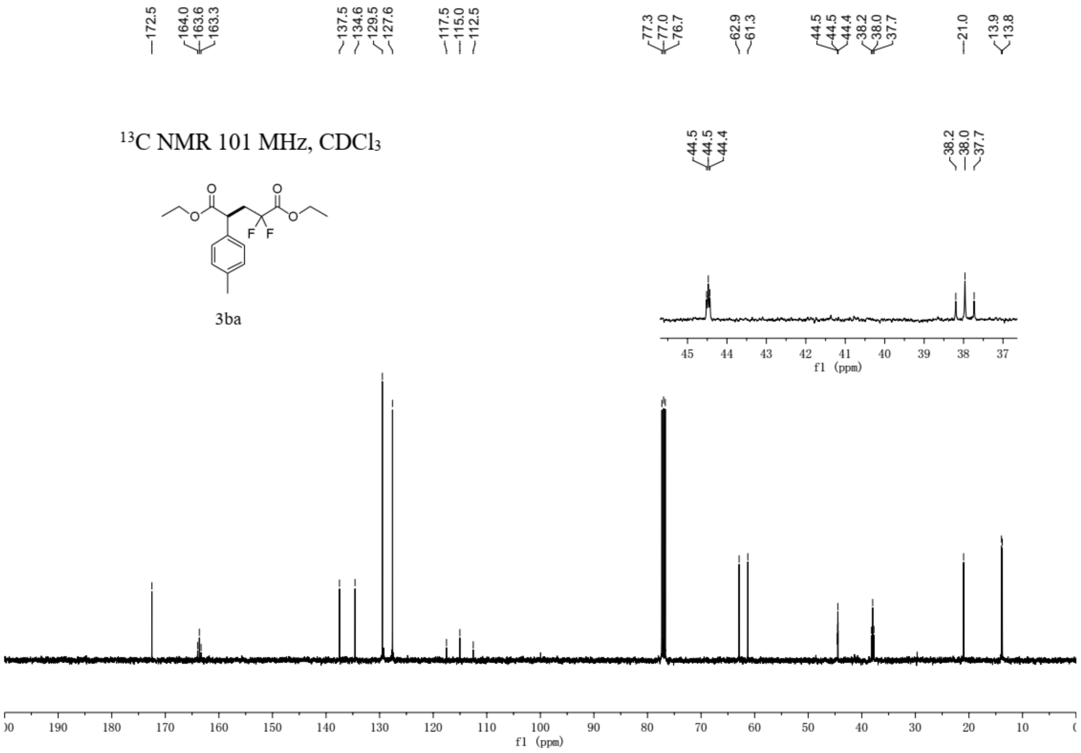
13C NMR 101 MHz, CDCl3
3ba
f1 (ppm)

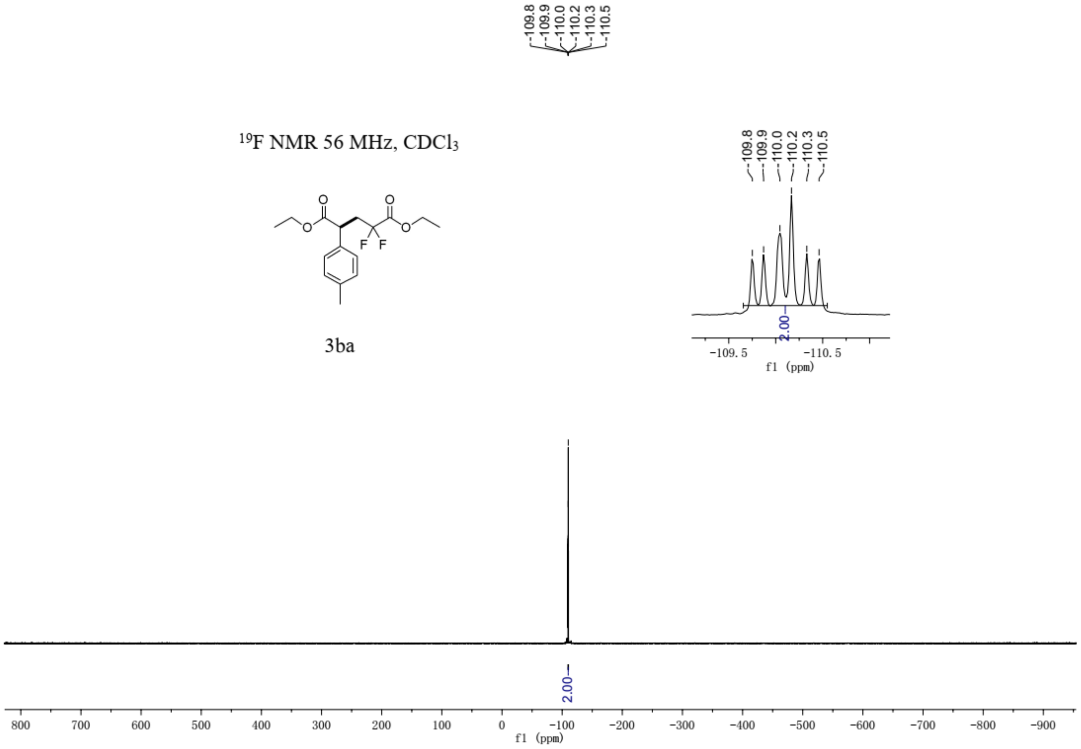
-109.8
-109.9
-110.0
-110.2
-110.3
-110.5
^{19}F NMR 56 MHz, CDCl$_3$
F F
3ba
2.00
-109.5
-110.5
f1 (ppm)
800 700 600 500 400 300 200 100 0 -100 -200 -300 -400 -500 -600 -700 -800 -900
f1 (ppm)

^{1}H NMR 400 MHz, $CDCl_3$

3ca

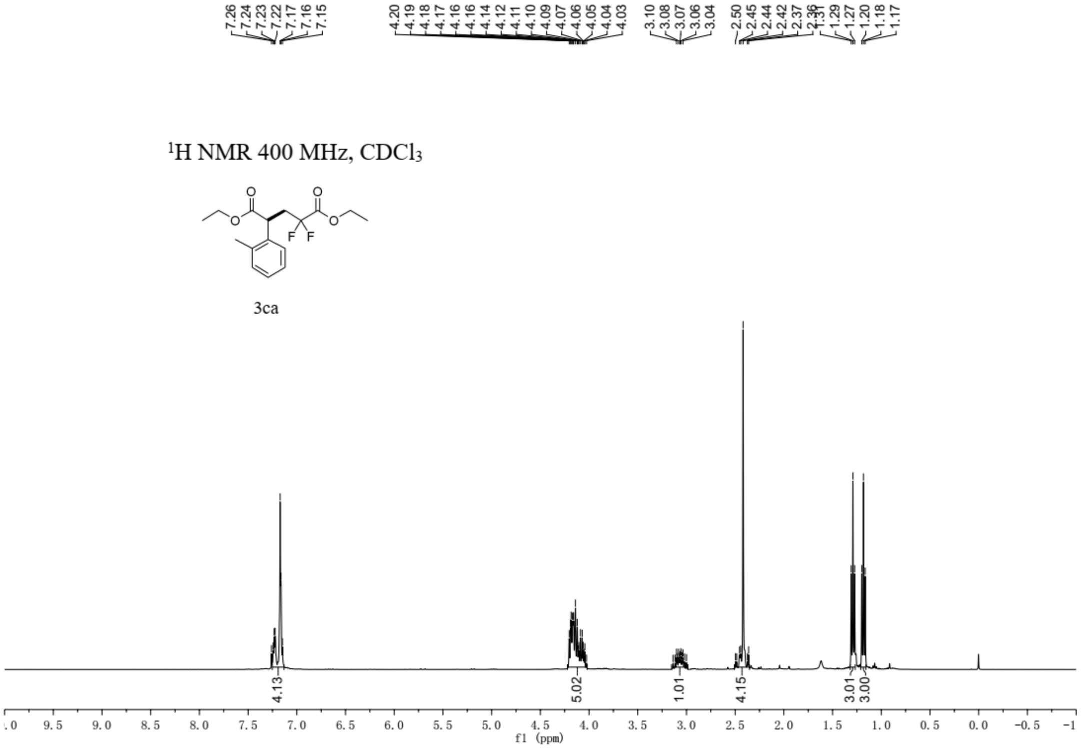

^{13}C NMR 101 MHz, $CDCl_3$

3ca

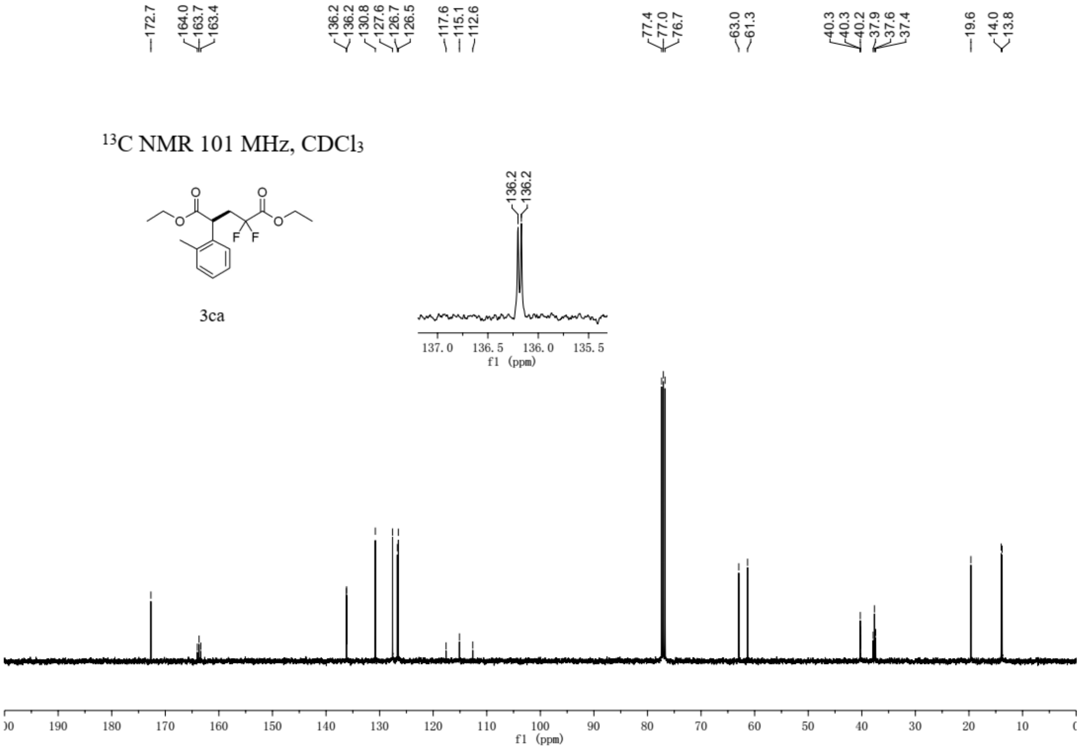

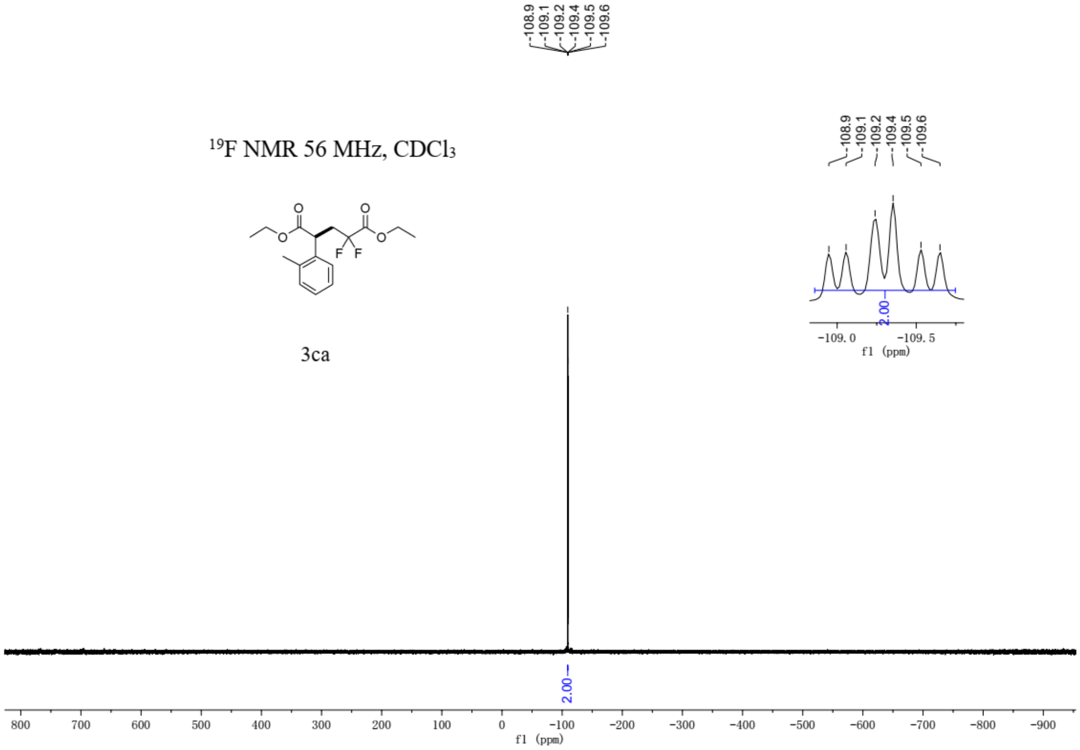
-108.9
-109.1
-109.2
-109.4
-109.5
-109.6
19F NMR 56 MHz, CDCl3
3ca
2.00
-109.0
-109.5
f1 (ppm)
800
700
600
500
400
300
200
100
0
-100
-200
-300
-400
-500
-600
-700
-800
-900

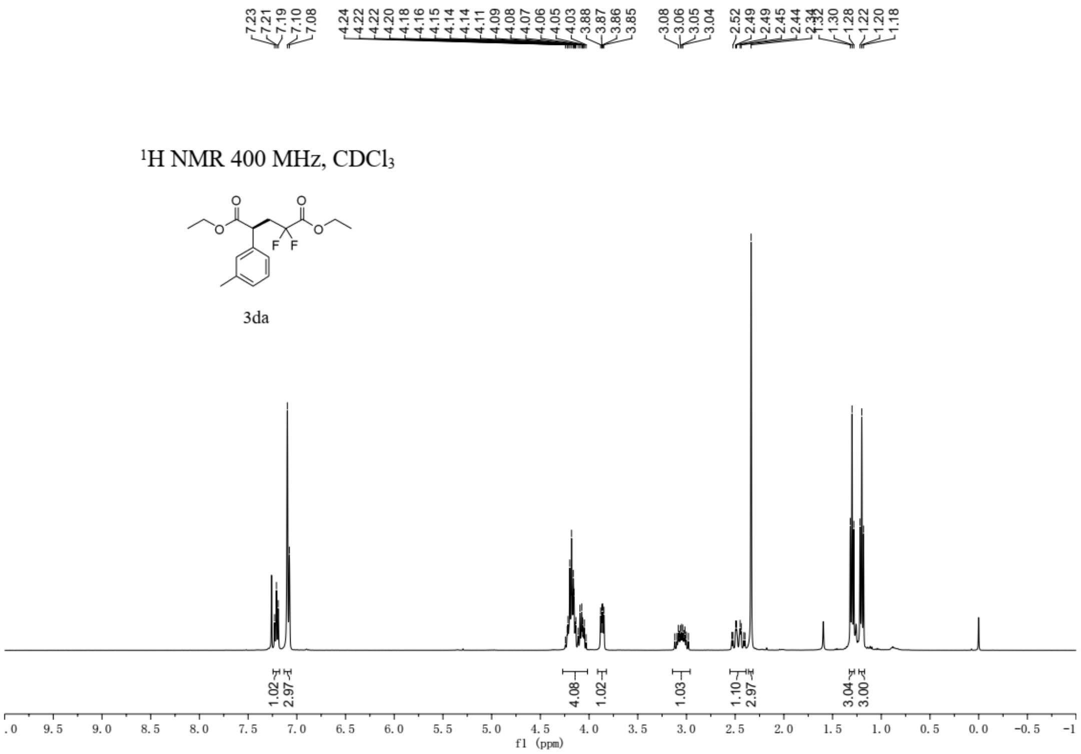
7.23 7.21 7.19 7.10 7.08 4.24 4.22 4.22 4.20 4.18 4.16 4.15 4.14 4.14 4.11 4.09 4.08 4.07 4.06 4.05 4.03 3.88 3.87 3.86 3.85 3.08 3.06 3.05 3.04 2.52 2.49 2.49 2.45 2.44 2.34 1.32 1.30 1.28 1.22 1.20 1.18
¹H NMR 400 MHz, CDCl₃
3da
1.02 2.97 4.08 1.02 1.03 1.10 2.97 3.04 3.00
f1 (ppm)

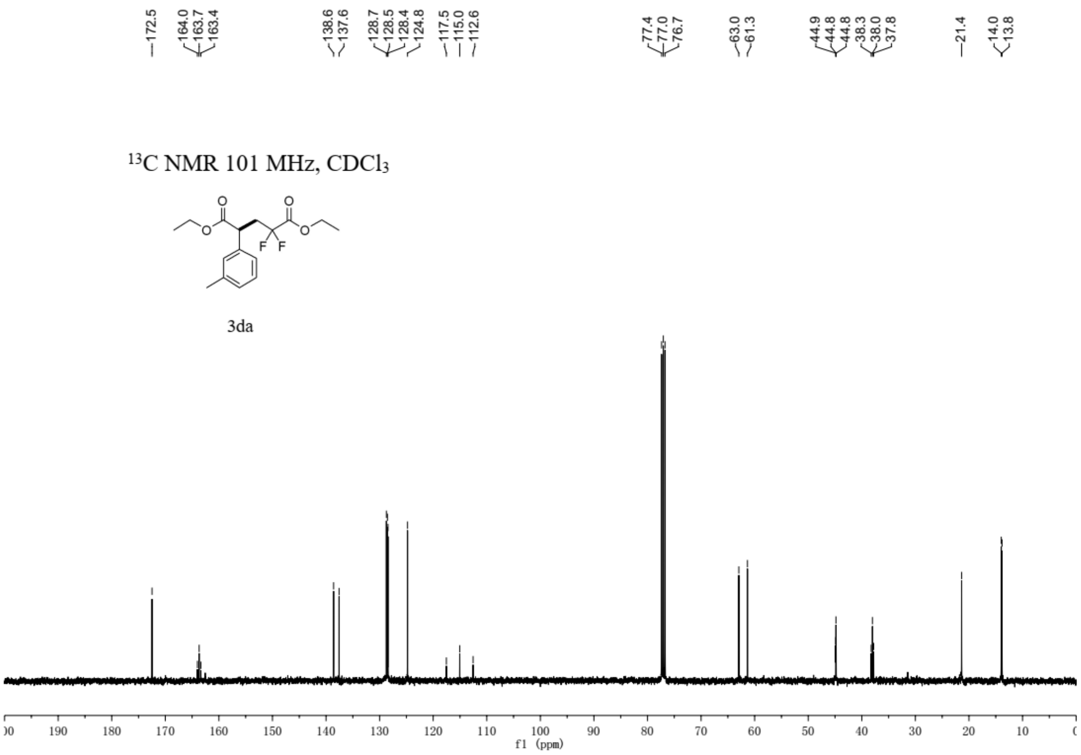
172.5 164.0 163.7 163.4 138.6 137.6 128.7 128.5 128.4 124.8 117.5 115.0 112.6 77.4 77.0 76.7 63.0 61.3 44.9 44.8 44.8 38.3 38.0 37.8 21.4 14.0 13.8
¹³C NMR 101 MHz, CDCl₃
3da
f1 (ppm)

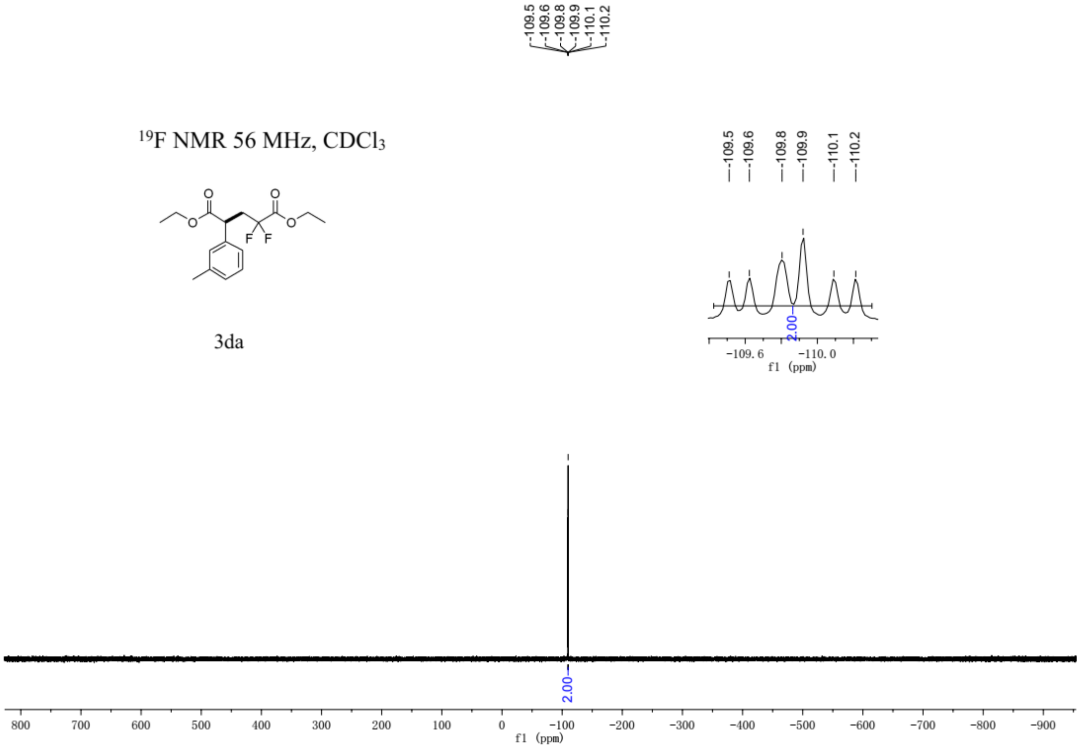
-109.5
-109.6
-109.8
-109.9
-110.1
-110.2
19F NMR 56 MHz, CDCl3
3da
2.00
-109.6
-110.0
f1 (ppm)
800
700
600
500
400
300
200
100
0
-100
-200
-300
-400
-500
-600
-700
-800
-900

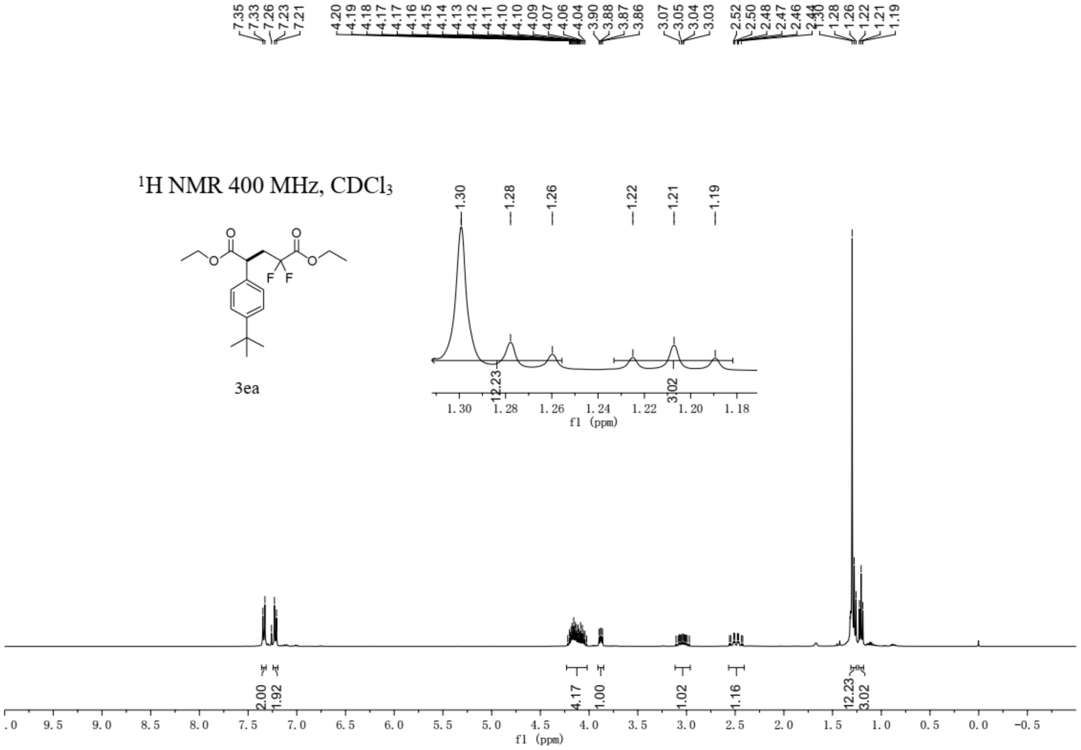
^{1}H NMR 400 MHz, CDCl$_3$
3ea
f1 (ppm)

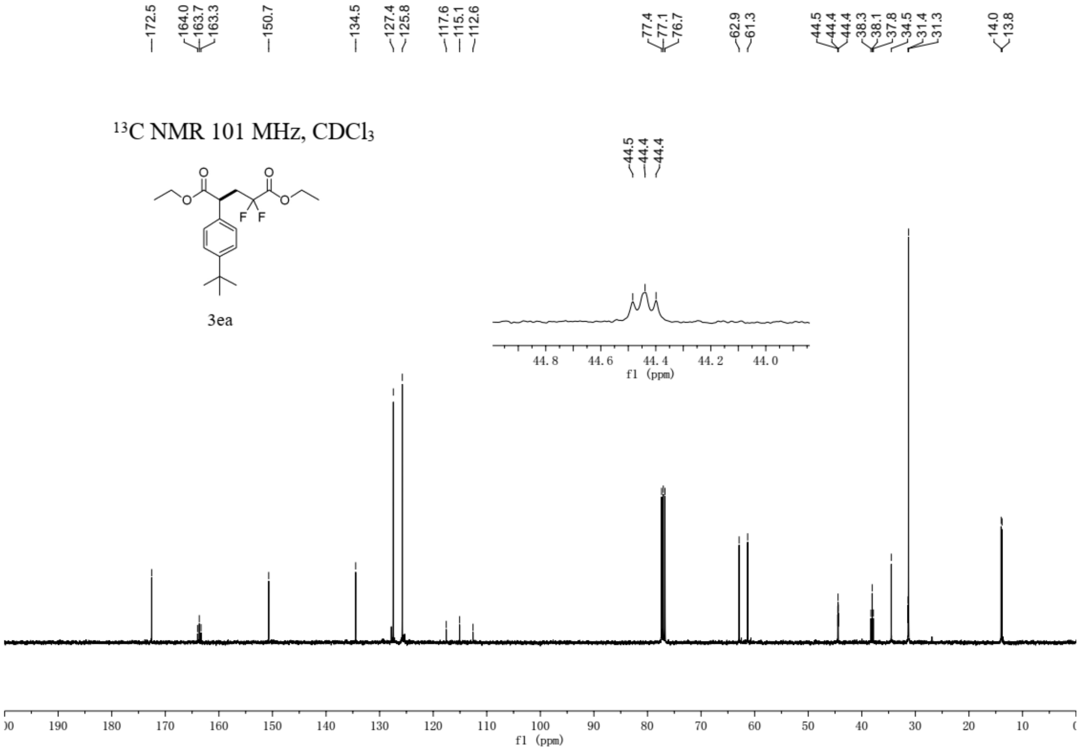
^{13}C NMR 101 MHz, CDCl$_3$
3ea
f1 (ppm)

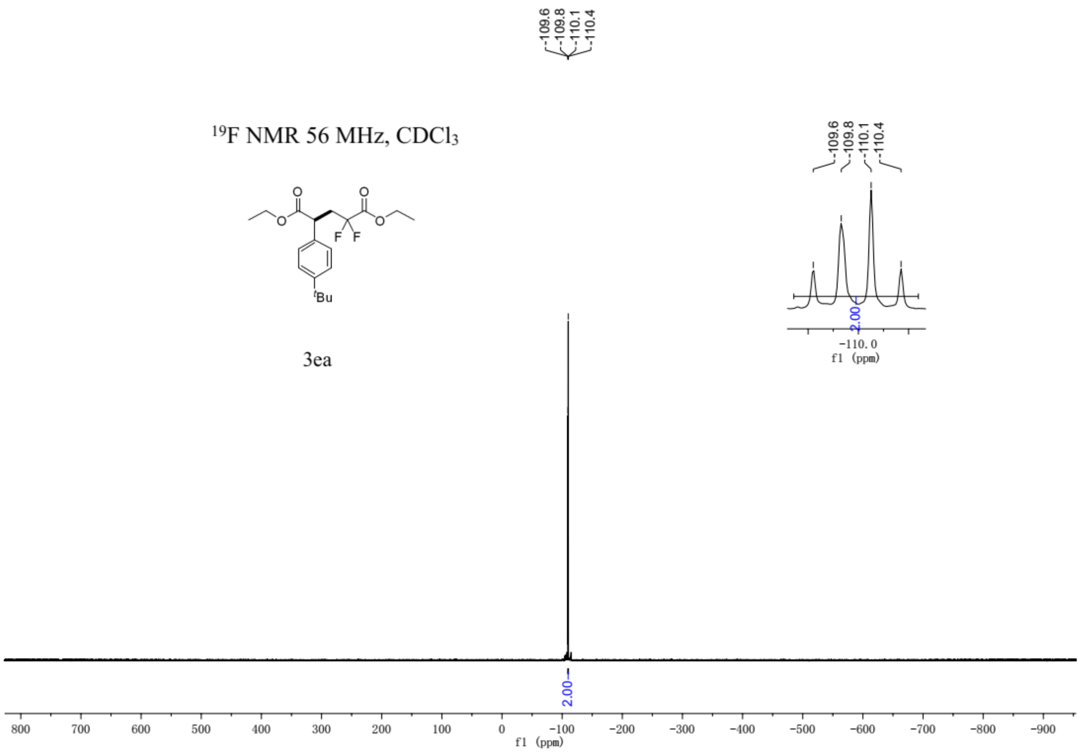
-109.6
-109.8
-110.1
-110.4
^{19}F NMR 56 MHz, $CDCl_3$
F F
^{t}Bu
3ea
2.00
-110.0
f1 (ppm)
800 700 600 500 400 300 200 100 0 -100 -200 -300 -400 -500 -600 -700 -800 -900
f1 (ppm)

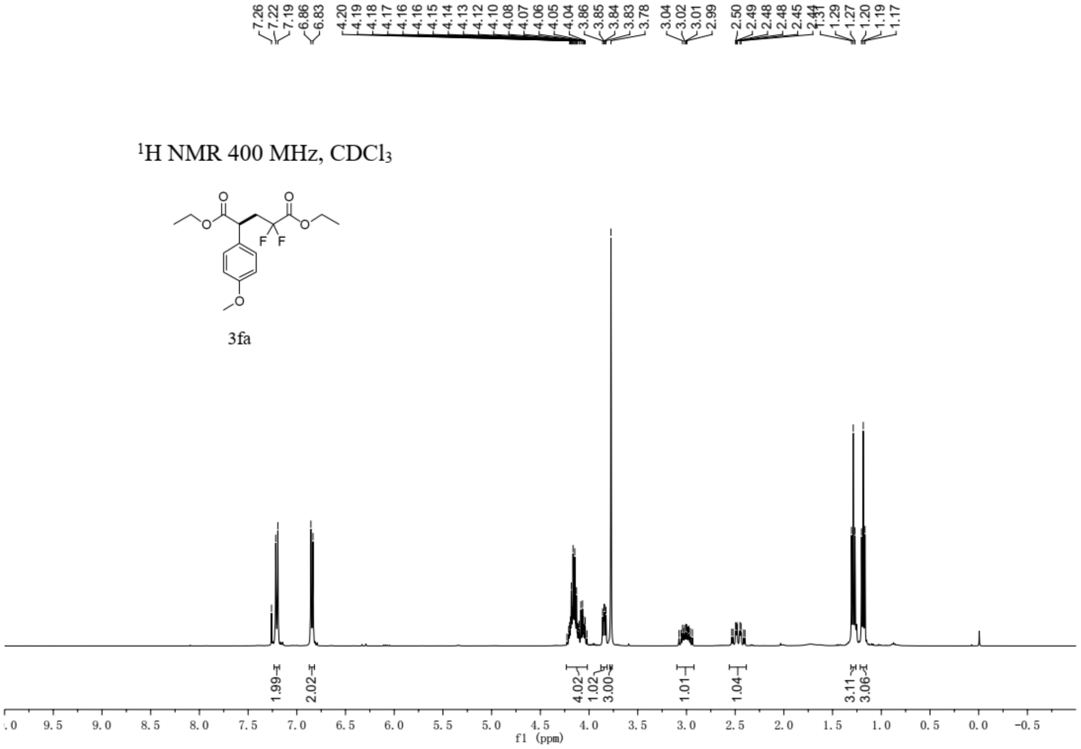

^{1}H NMR 400 MHz, $CDCl_3$
3fa
f1 (ppm)

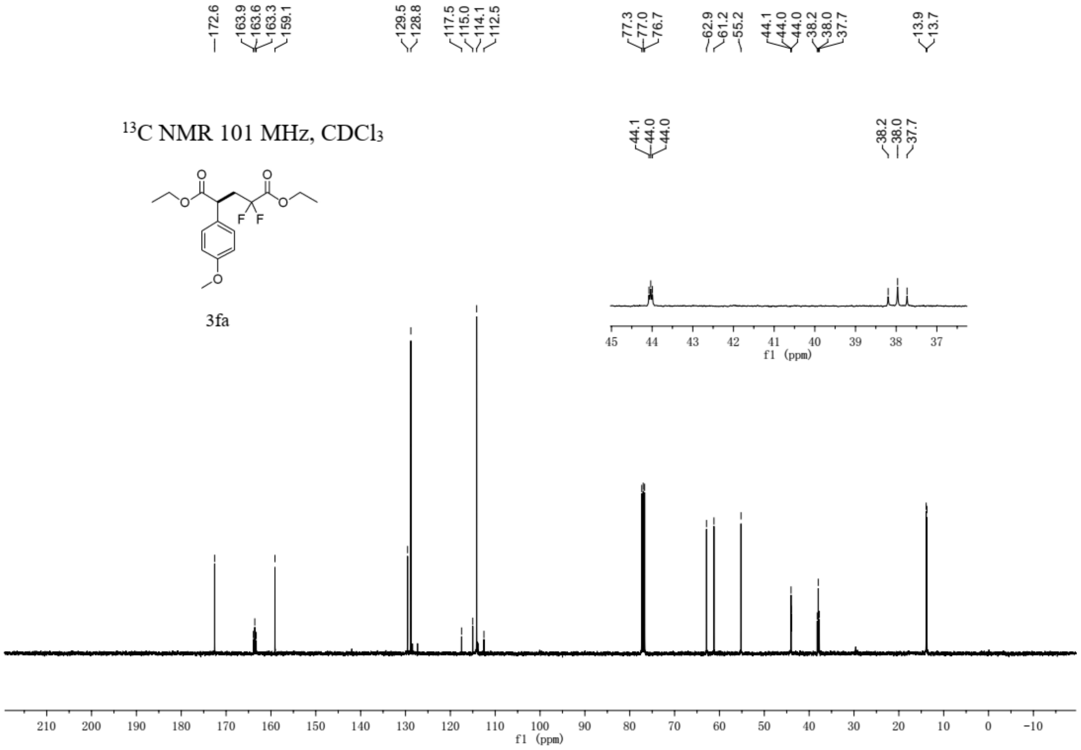

^{13}C NMR 101 MHz, $CDCl_3$
3fa
f1 (ppm)

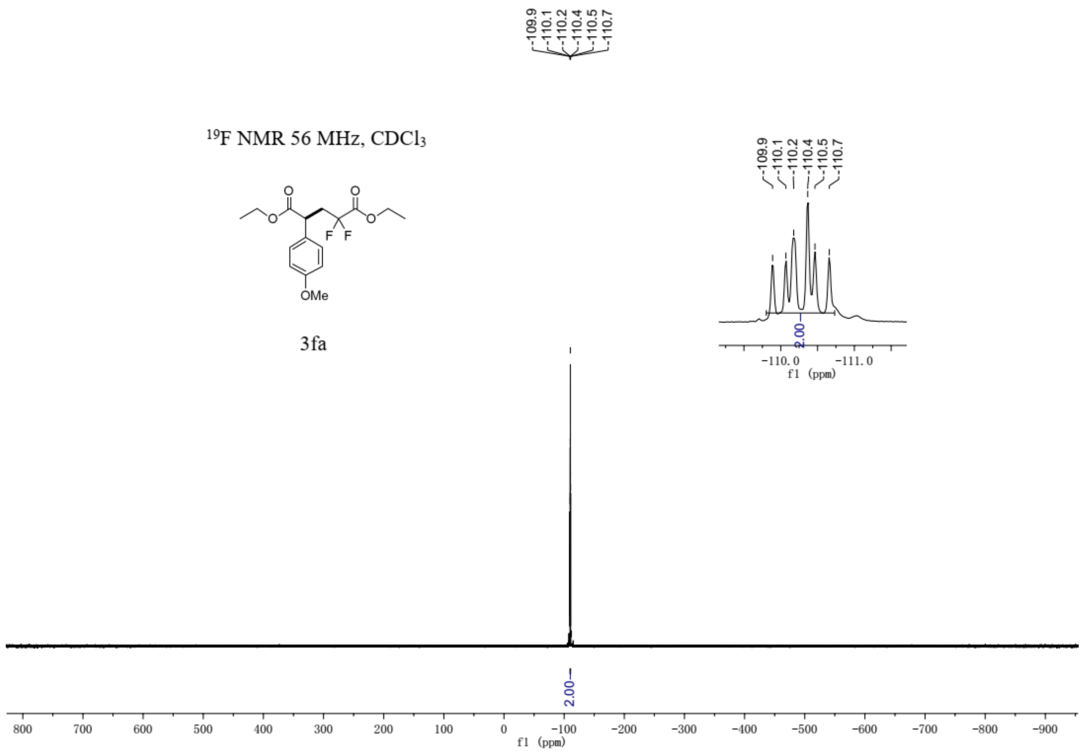

^{19}F NMR 56 MHz, CDCl$_3$
OMe
F F
3fa
-109.9
-110.1
-110.2
-110.4
-110.5
-110.7
2.00
-110.0
-111.0
f1 (ppm)
800 700 600 500 400 300 200 100 0 −100 −200 −300 −400 −500 −600 −700 −800 −900

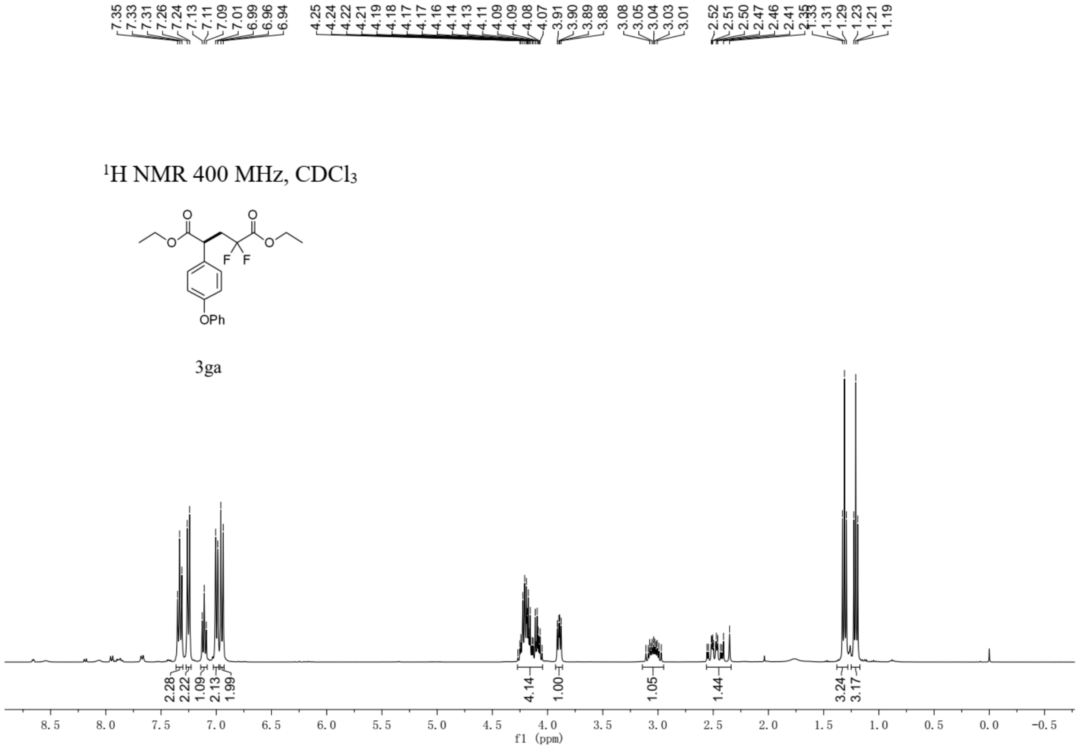

1H NMR 400 MHz, CDCl3
OPh
3ga
f1 (ppm)

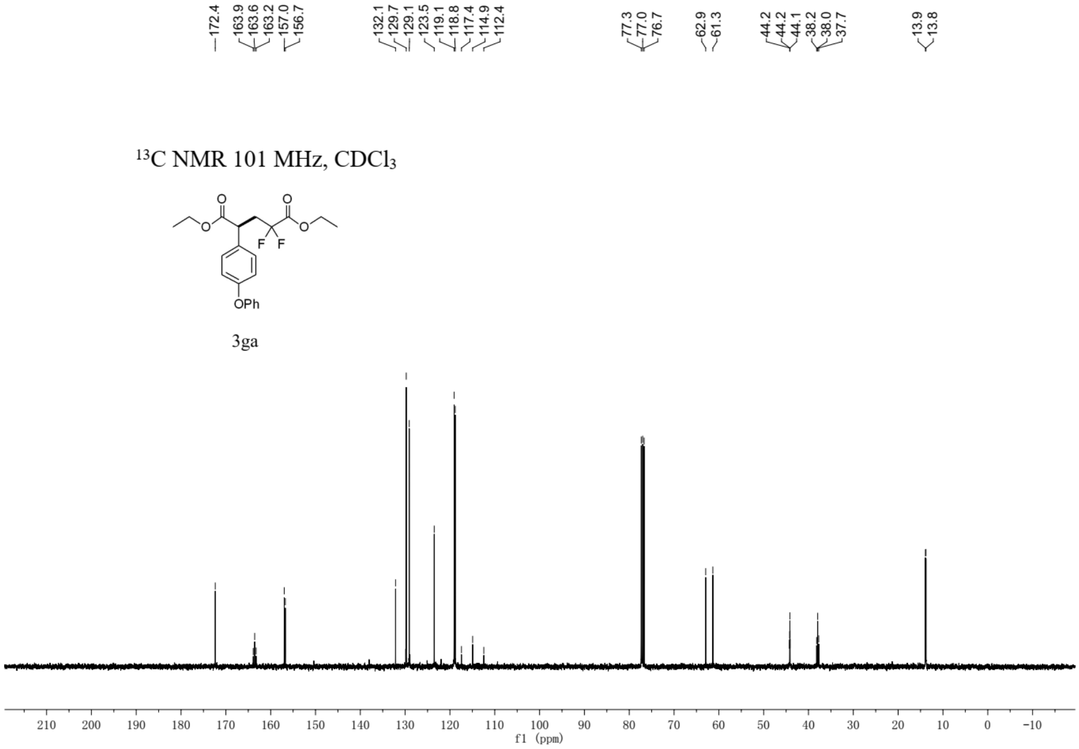

13C NMR 101 MHz, CDCl3
OPh
3ga
f1 (ppm)

^{19}F NMR 56 MHz, $CDCl_3$

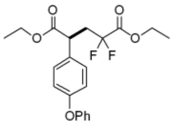

3ga

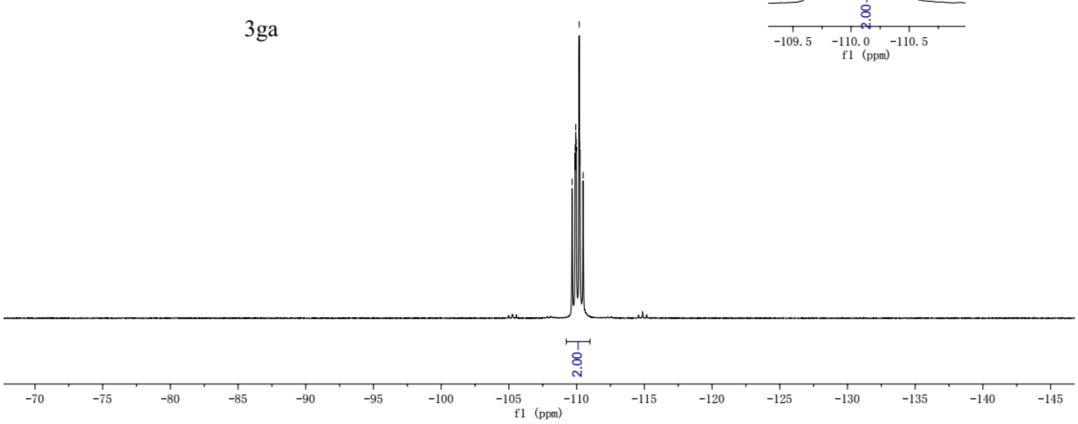

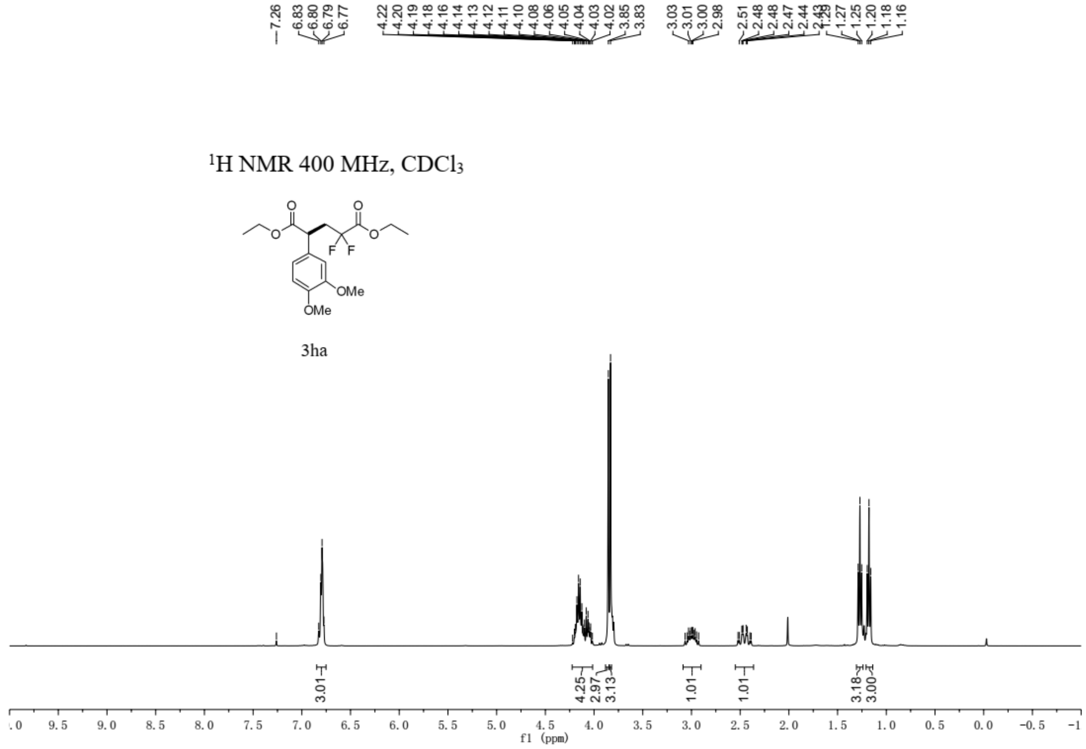

1H NMR 400 MHz, CDCl3
OMe
OMe
3ha
7.26
6.83
6.80
6.79
6.77
4.22
4.20
4.19
4.18
4.16
4.14
4.13
4.12
4.11
4.10
4.08
4.06
4.05
4.04
4.03
4.02
3.85
3.83
3.03
3.01
3.00
2.98
2.51
2.48
2.47
2.44
1.27
1.25
1.20
1.18
1.16
3.01
4.25
2.97
3.13
1.01
1.01
3.18
3.00
f1 (ppm)

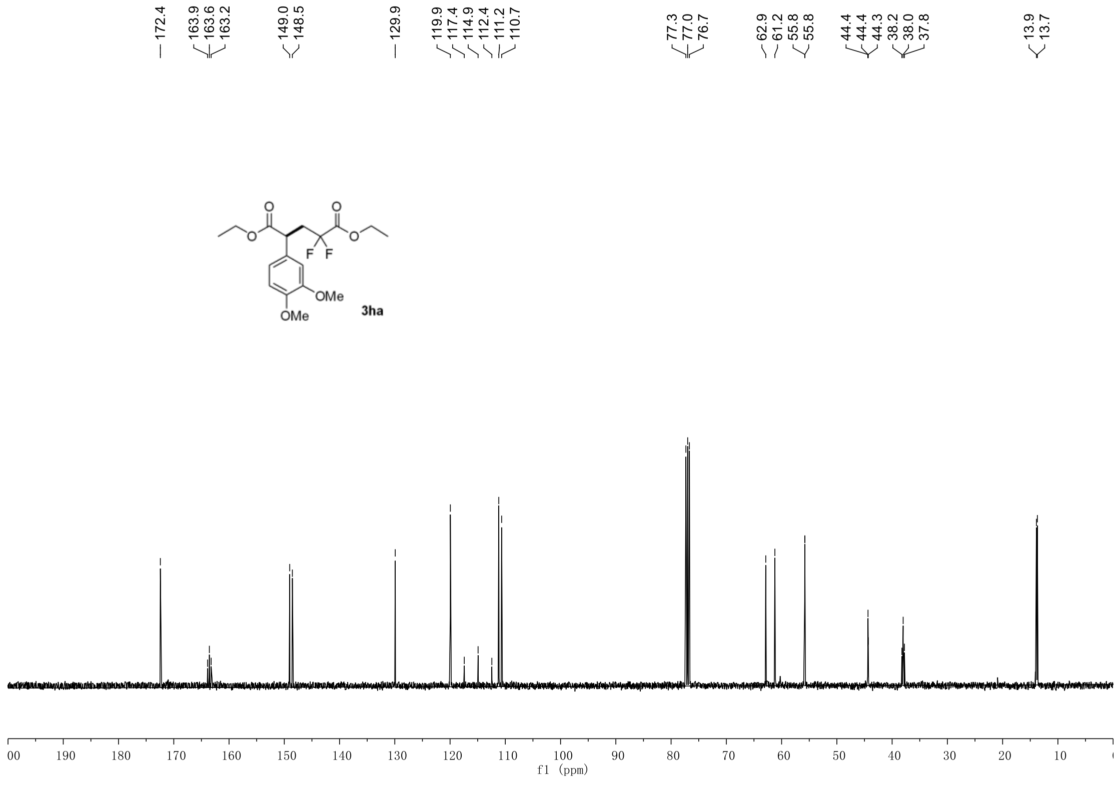

172.4
163.9
163.6
163.2
149.0
148.5
129.9
119.9
117.4
114.9
112.4
111.2
110.7
77.3
77.0
76.7
62.9
61.2
55.8
55.8
44.4
44.4
44.3
38.2
38.0
37.8
13.9
13.7
OMe
OMe
3ha
f1 (ppm)

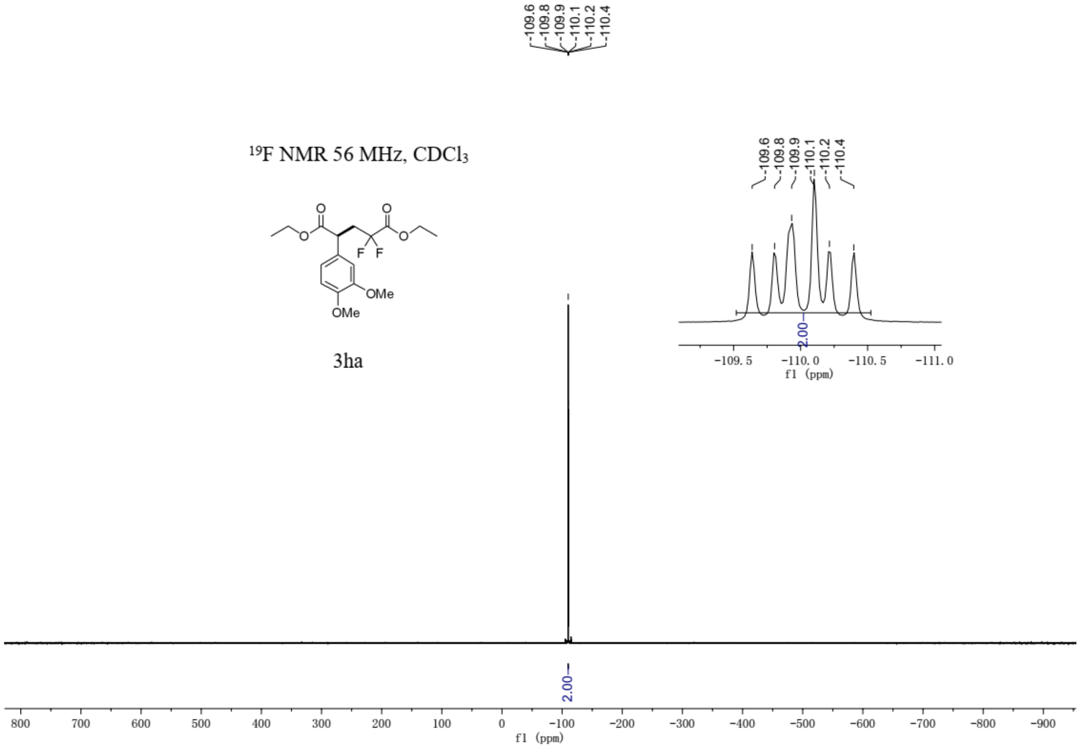
-109.6
-109.8
-109.9
-110.1
-110.2
-110.4
^{19}F NMR 56 MHz, CDCl$_3$
O
O
O
O
F
F
OMe
OMe
3ha
2.00
-109.5
-110.0
-110.5
-111.0
f1 (ppm)
800
700
600
500
400
300
200
100
0
-100
-200
-300
-400
-500
-600
-700
-800
-900

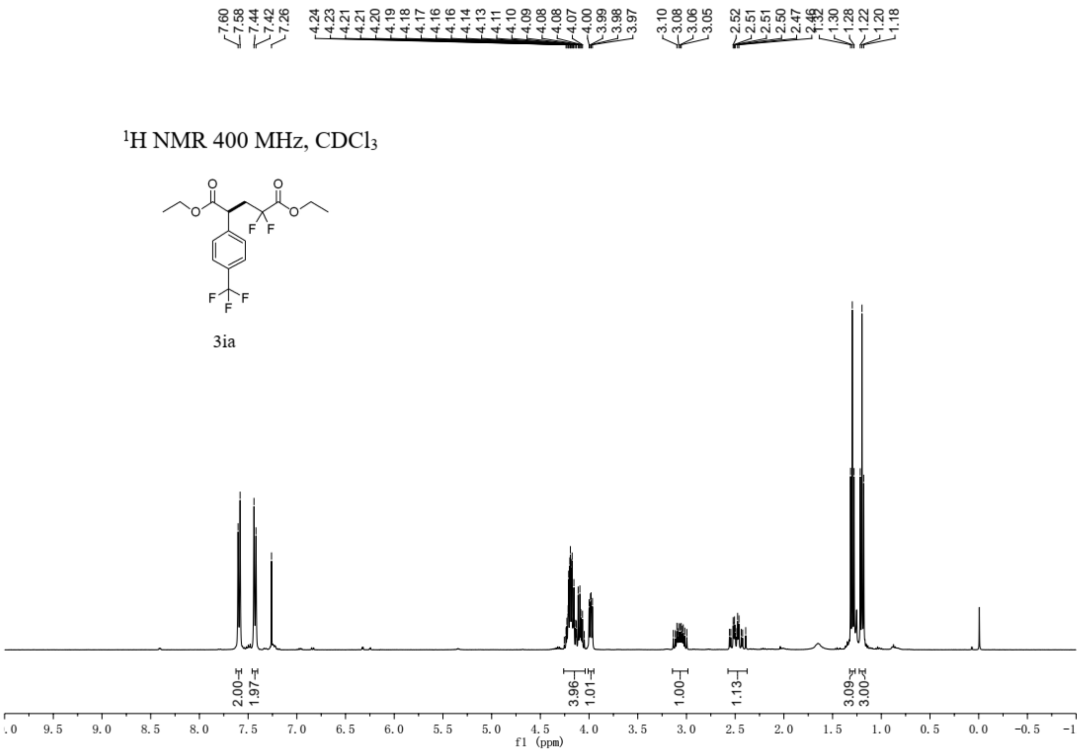
7.60
7.58
7.44
7.42
7.26
4.24
4.23
4.21
4.21
4.20
4.19
4.18
4.17
4.16
4.16
4.14
4.13
4.11
4.10
4.09
4.08
4.08
4.07
4.00
3.99
3.98
3.97
3.10
3.08
3.06
3.05
2.52
2.51
2.51
2.50
2.47
1.32
1.30
1.28
1.22
1.20
1.18
1H NMR 400 MHz, CDCl3
3ia
2.00
1.97
3.96
1.01
1.00
1.13
3.09
3.00
f1 (ppm)

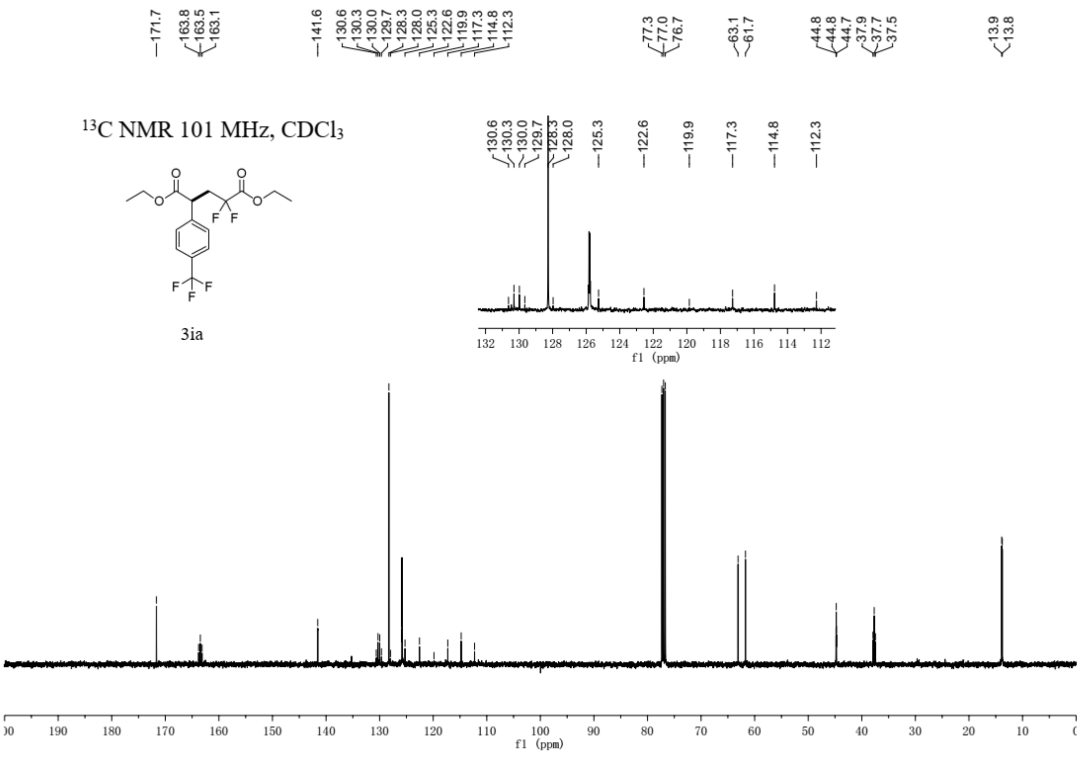
171.7
163.8
163.5
163.1
141.6
130.6
130.3
130.0
129.7
128.3
128.0
125.3
122.6
119.9
117.3
114.8
112.3
77.3
77.0
76.7
63.1
61.7
44.8
44.8
44.7
37.9
37.7
37.5
13.9
13.8
13C NMR 101 MHz, CDCl3
3ia
f1 (ppm)

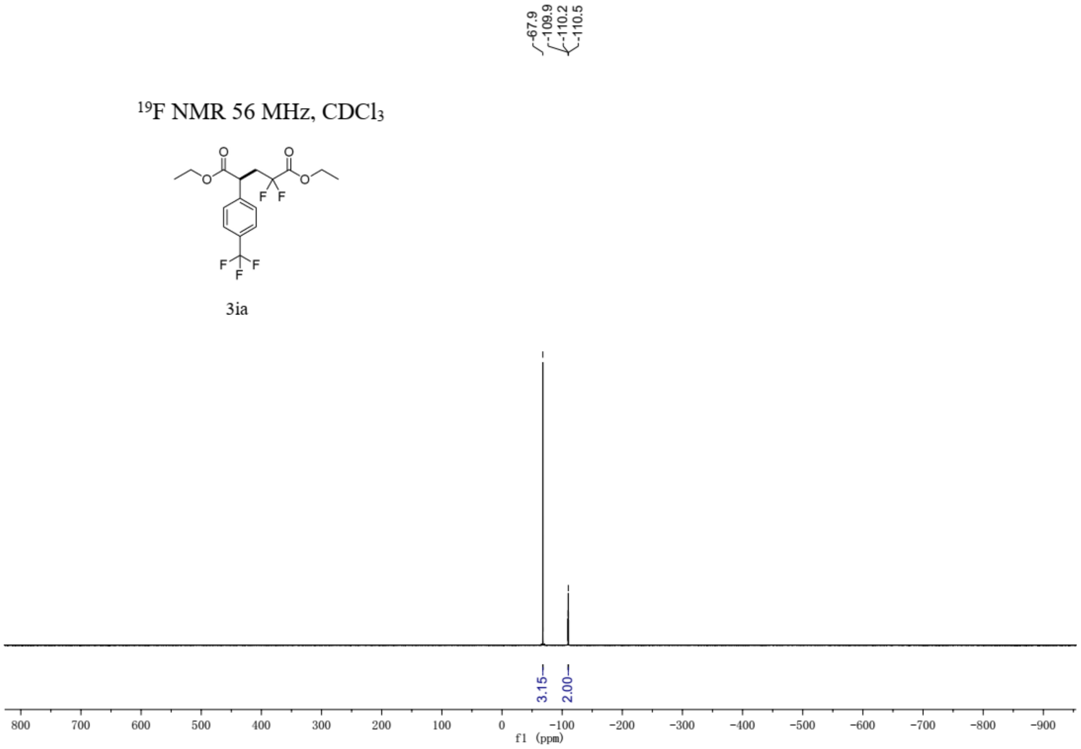
-67.9
-109.9
-110.2
-110.5
19F NMR 56 MHz, CDCl3
3ia
3.15
2.00
800 700 600 500 400 300 200 100 0 −100 −200 −300 −400 −500 −600 −700 −800 −900
f1 (ppm)

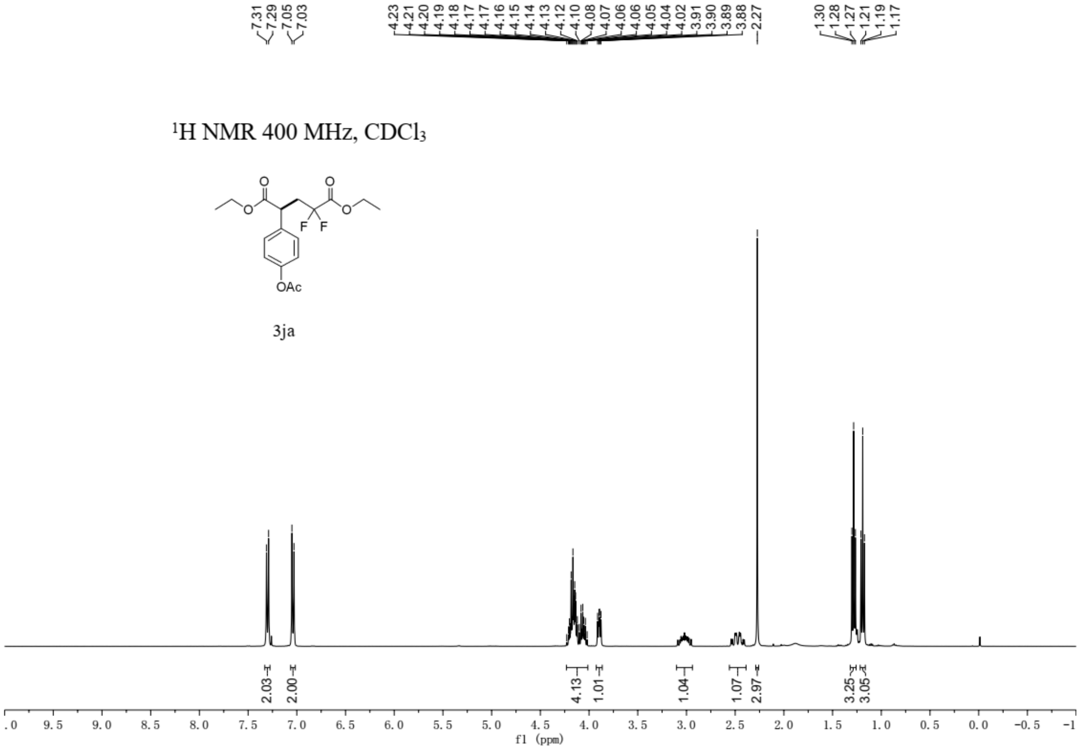
7.31
7.29
7.05
7.03
4.23 4.21 4.20 4.19 4.18 4.17 4.17 4.16 4.15 4.14 4.13 4.12 4.10 4.08 4.07 4.06 4.06 4.05 4.04 4.02 3.91 3.90 3.89 3.88
2.27
1.30 1.28 1.27 1.21 1.19 1.17
¹H NMR 400 MHz, CDCl₃
OAc
F F
3ja
2.03
2.00
4.13
1.01
1.04
1.07
2.97
3.25
3.05
f1 (ppm)

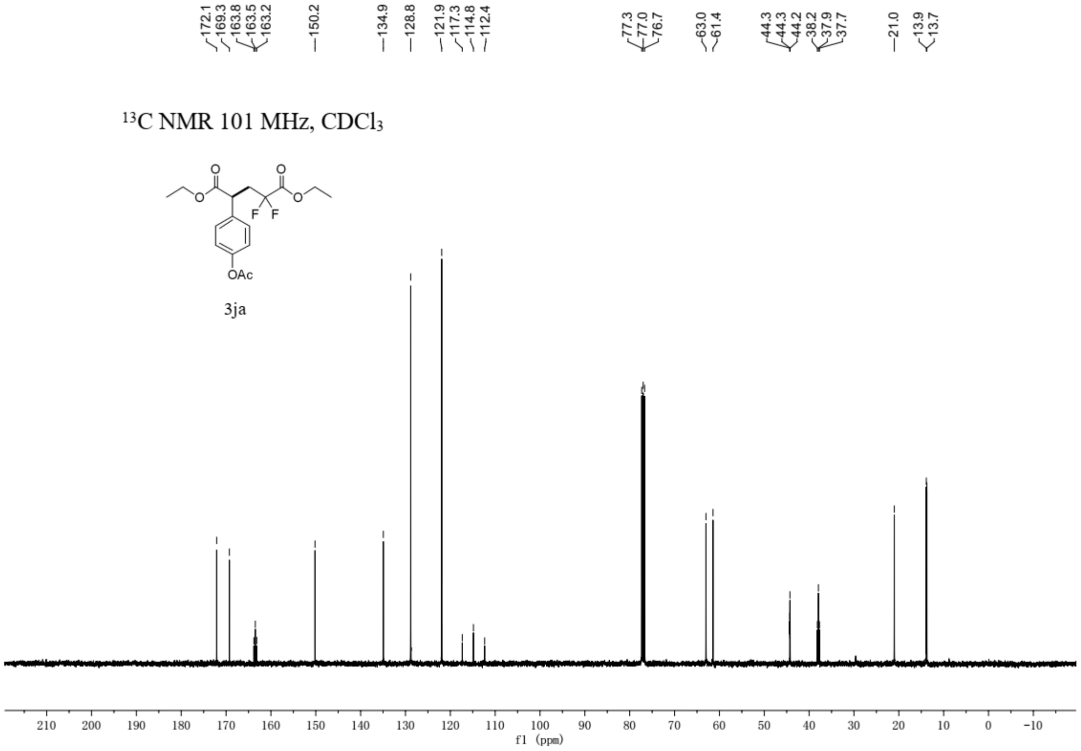
172.1 169.3 163.8 163.5 163.2
150.2
134.9
128.8
121.9 117.3 114.8 112.4
77.3 77.0 76.7
63.0 61.4
44.3 44.3 44.2 38.2 37.9 37.7
21.0
13.9 13.7
¹³C NMR 101 MHz, CDCl₃
OAc
F F
3ja
f1 (ppm)

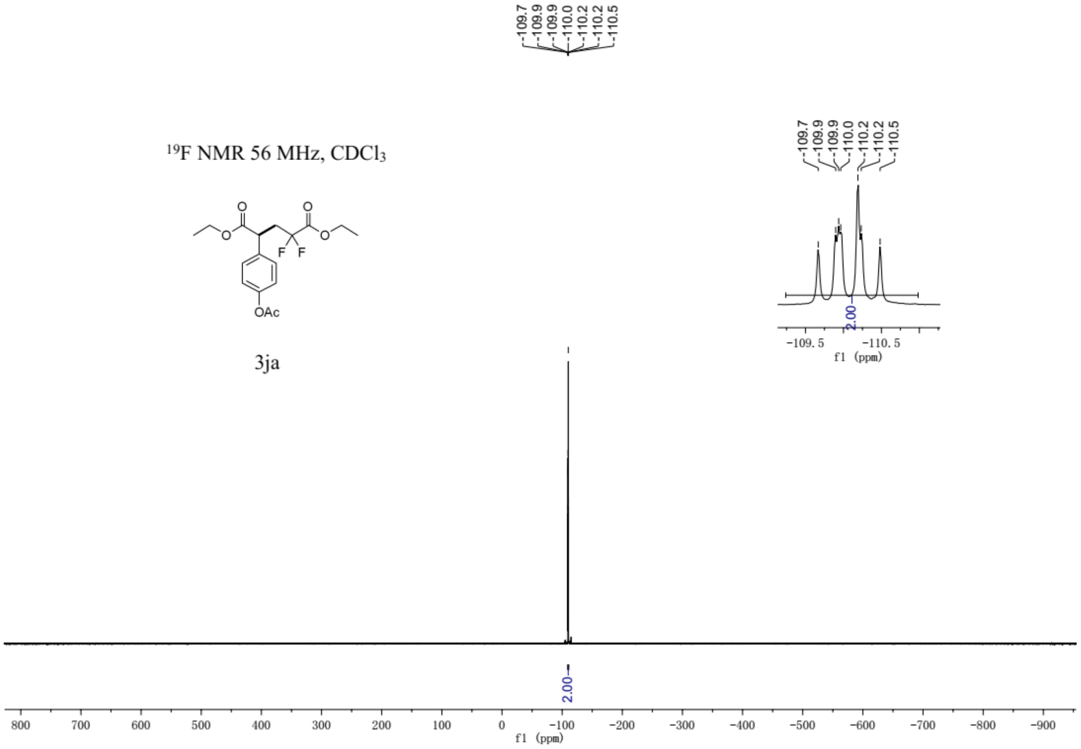

-109.7
-109.9
-109.9
-110.0
-110.2
-110.2
-110.5
19F NMR 56 MHz, CDCl3
OAc
F
F
3ja
2.00
−109.5
−110.5
f1 (ppm)
800 700 600 500 400 300 200 100 0 −100 −200 −300 −400 −500 −600 −700 −800 −900
f1 (ppm)

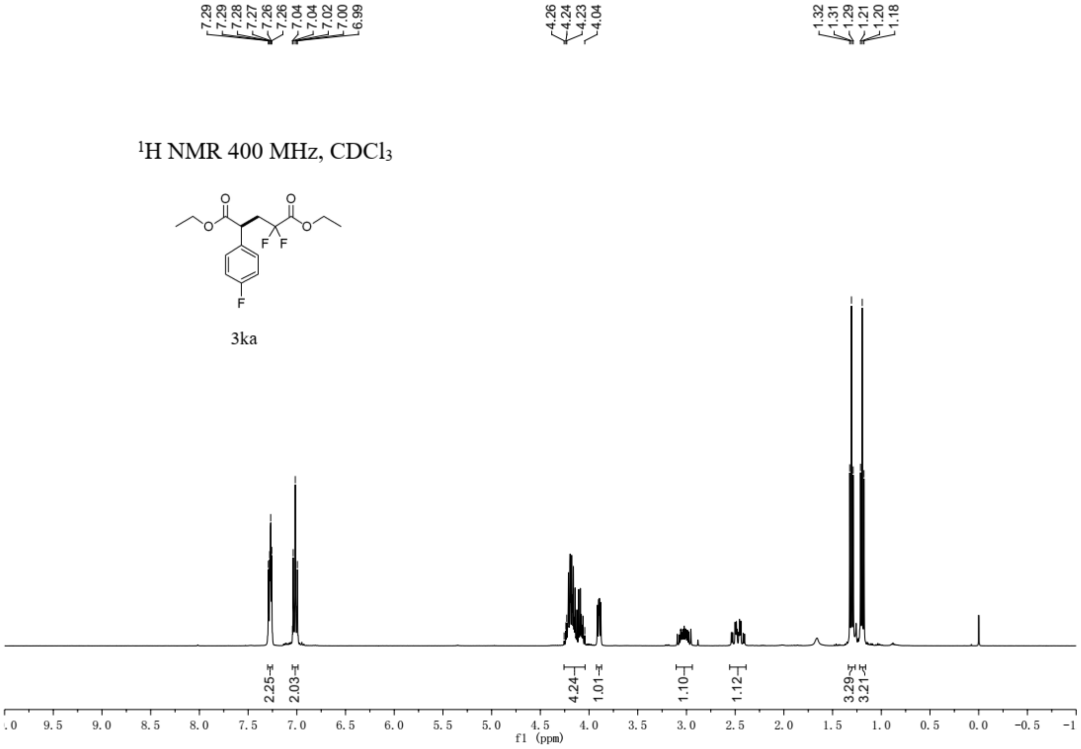
7.29
7.29
7.28
7.27
7.26
7.26
7.04
7.04
7.02
7.00
6.99
4.26
4.24
4.23
4.04
1.32
1.31
1.29
1.21
1.20
1.18
^{1}H NMR 400 MHz, $CDCl_3$
3ka
2.25
2.03
4.24
1.01
1.10
1.12
3.29
3.21
f1 (ppm)

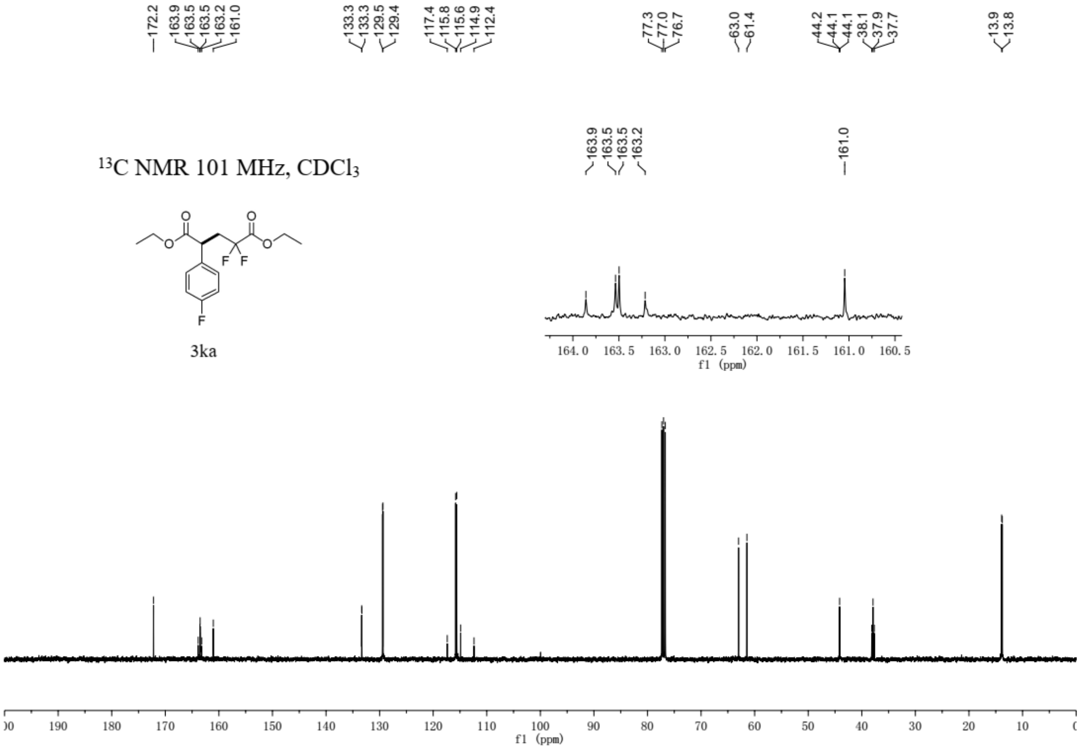
172.2
163.9
163.5
163.5
163.2
161.0
133.3
133.3
129.5
129.4
117.4
115.8
115.6
114.9
112.4
77.3
77.0
76.7
63.0
61.4
44.2
44.1
44.1
38.1
37.9
37.7
13.9
13.8
^{13}C NMR 101 MHz, $CDCl_3$
3ka
163.9
163.5
163.5
163.2
161.0
f1 (ppm)
f1 (ppm)

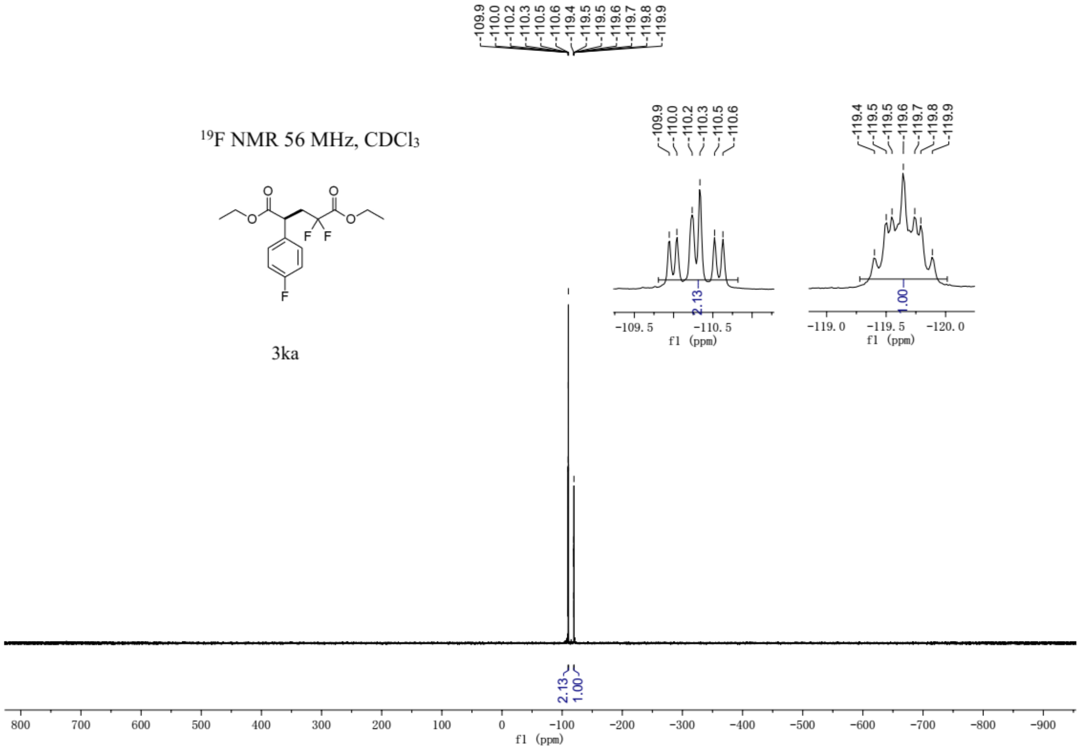
-109.9
-110.0
-110.2
-110.3
-110.5
-110.6
-119.4
-119.5
-119.5
-119.6
-119.7
-119.8
-119.9
^{19}F NMR 56 MHz, CDCl$_3$
3ka
2.13
1.00
-109.5
-110.5
f1 (ppm)
-119.0
-119.5
-120.0
f1 (ppm)
800
700
600
500
400
300
200
100
0
−100
−200
−300
−400
−500
−600
−700
−800
−900
f1 (ppm)

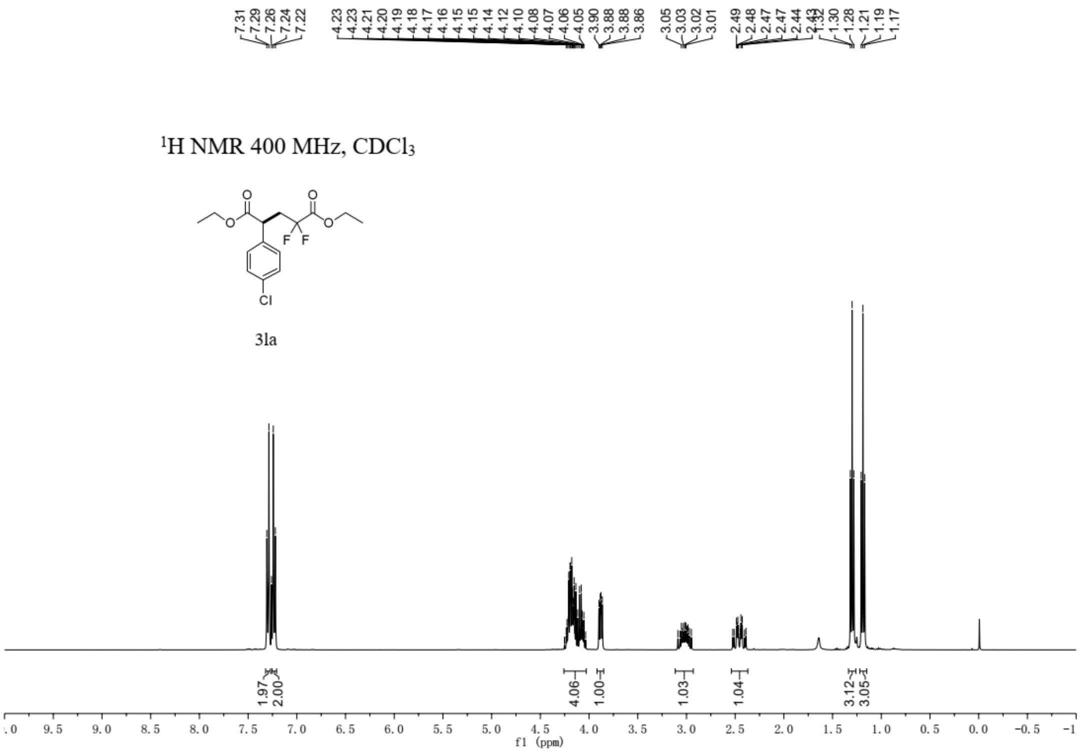
^{1}H NMR 400 MHz, CDCl$_3$
3la

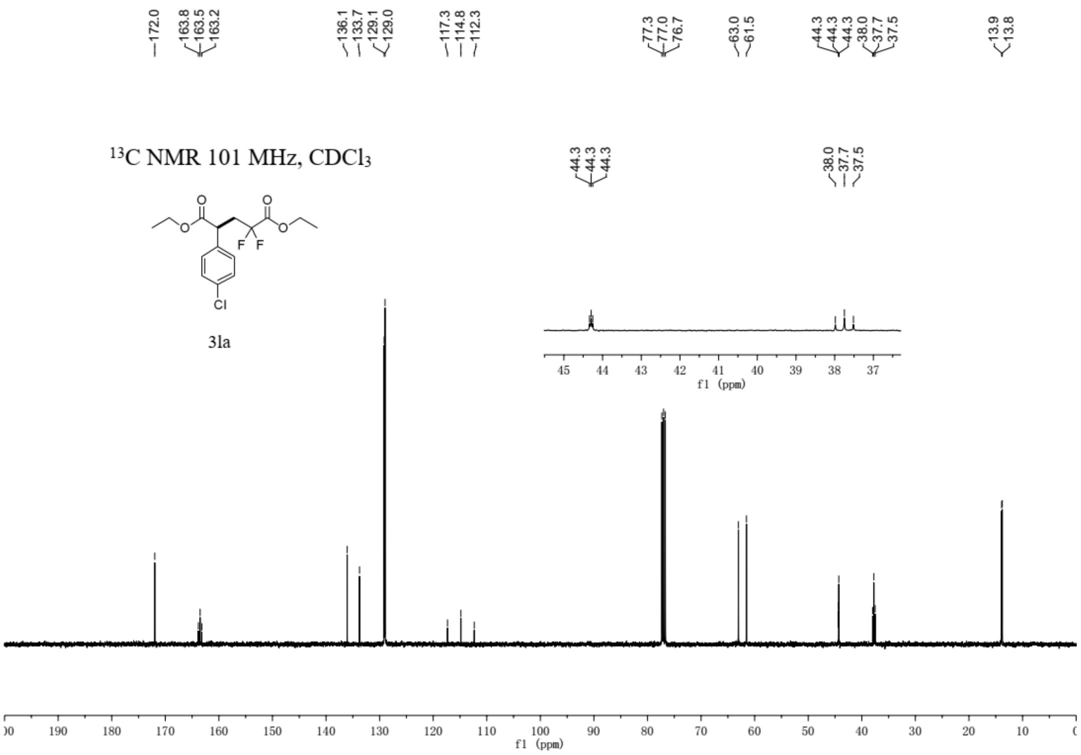
^{13}C NMR 101 MHz, CDCl$_3$
3la

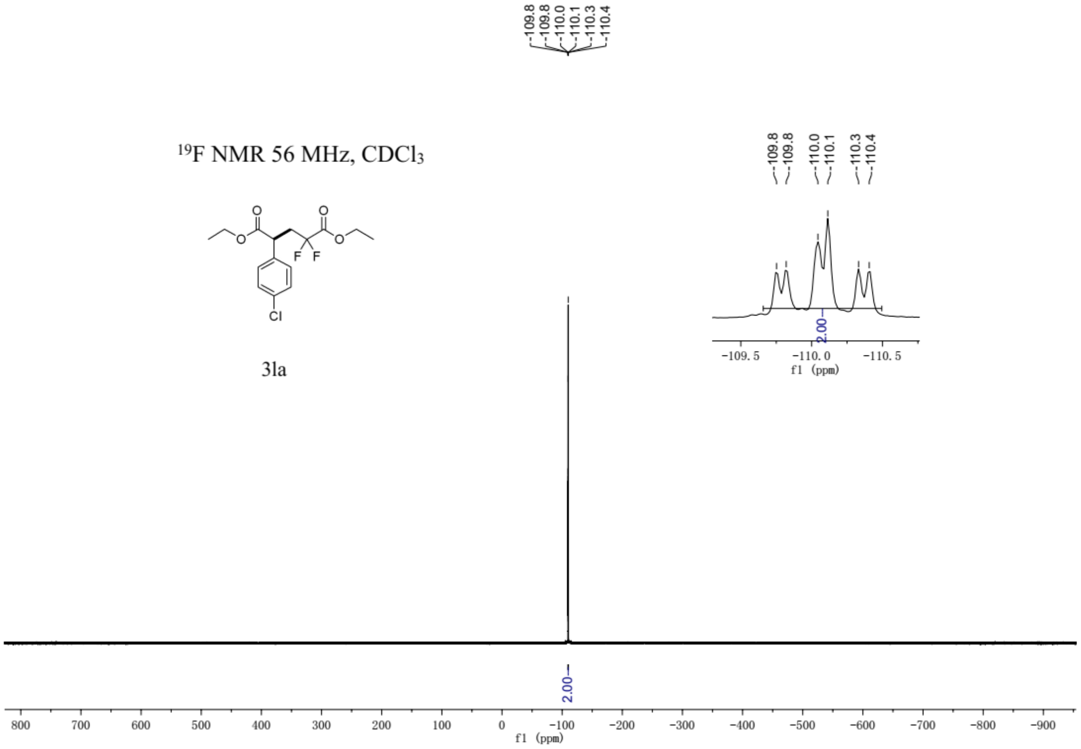
-109.8
-109.8
-110.0
-110.1
-110.3
-110.4
19F NMR 56 MHz, CDCl3
F F
Cl
3la
2.00
-109.5
-110.0
-110.5
f1 (ppm)
800
700
600
500
400
300
200
100
0
-100
-200
-300
-400
-500
-600
-700
-800
-900

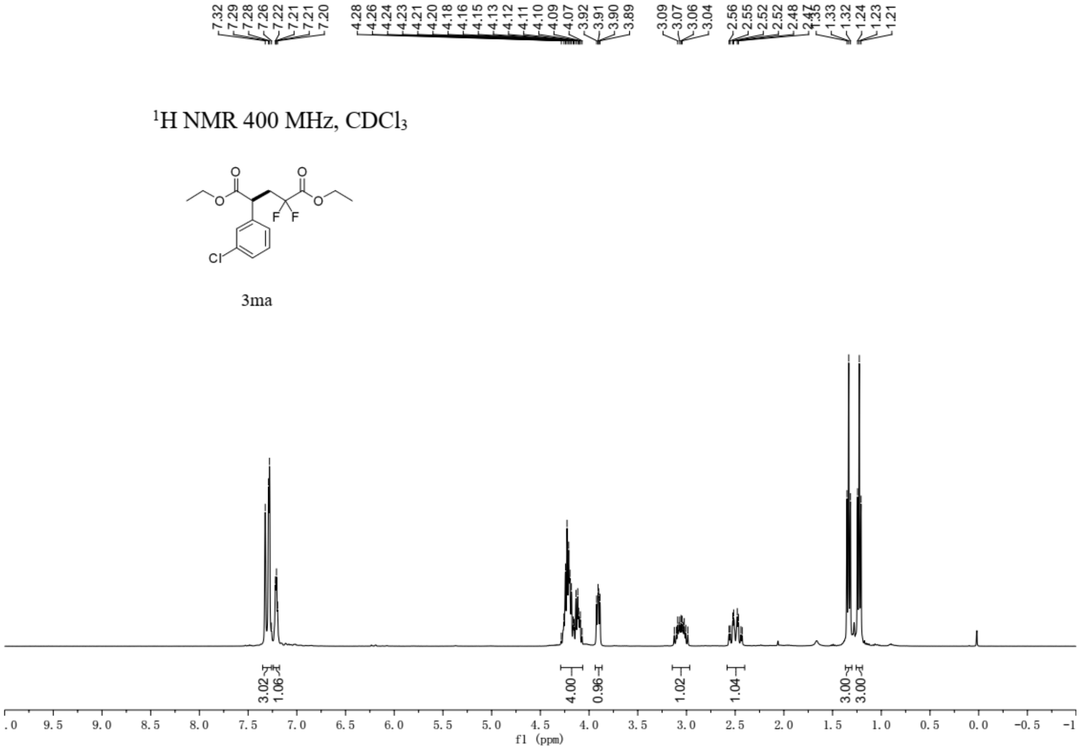

^{1}H NMR 400 MHz, $CDCl_3$
3ma

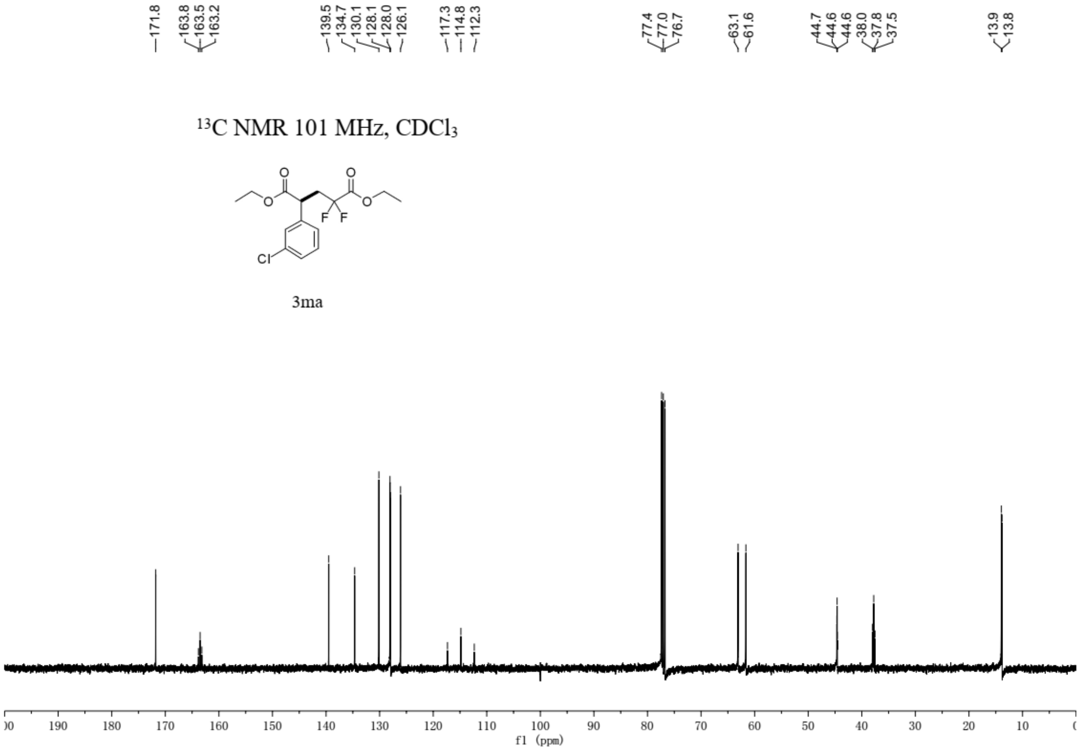

^{13}C NMR 101 MHz, $CDCl_3$
3ma

^{19}F NMR 56 MHz, $CDCl_3$

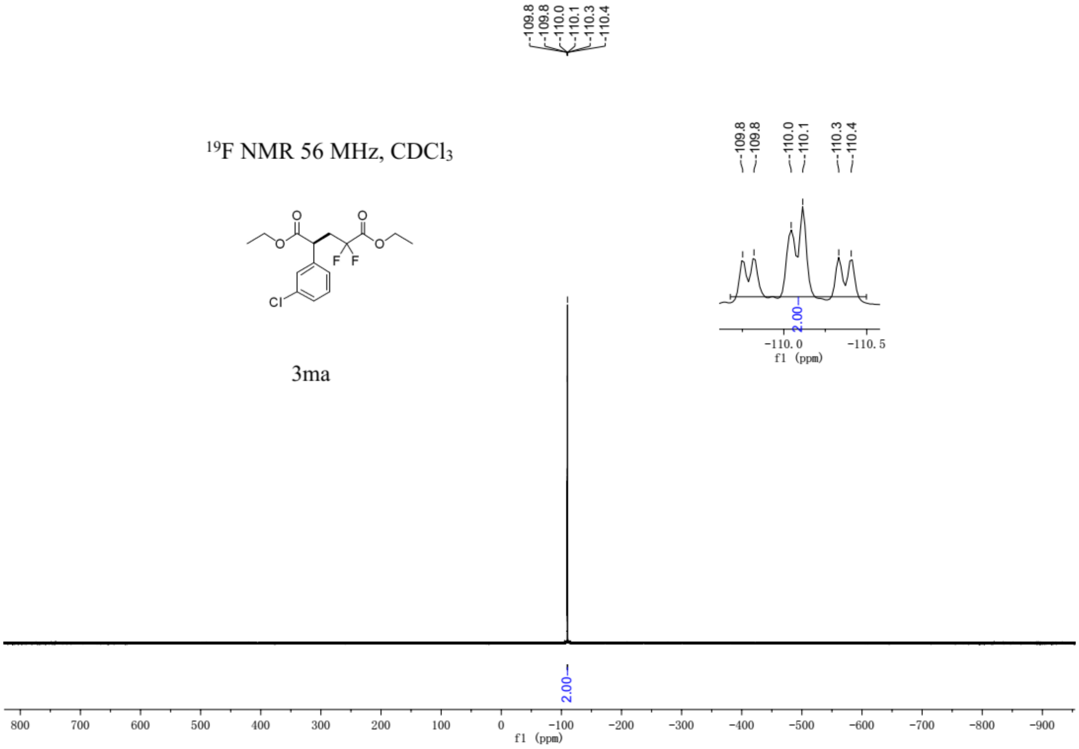

3ma

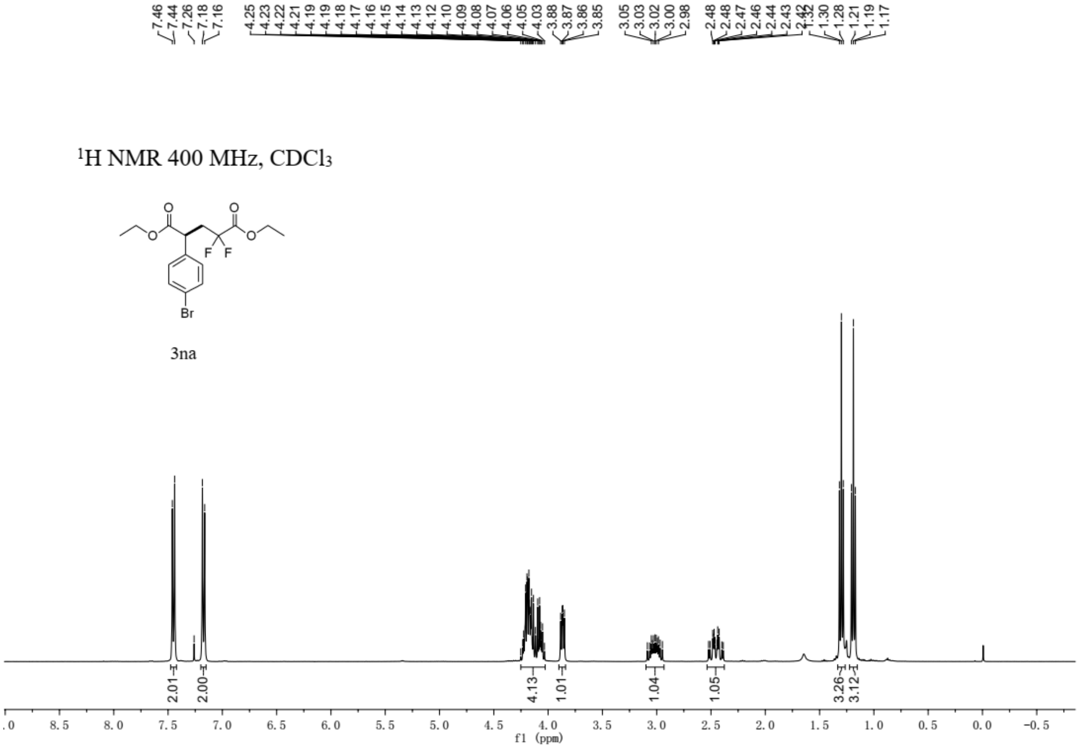
7.46
7.44
7.26
7.18
7.16
4.25
4.23
4.22
4.21
4.19
4.19
4.18
4.17
4.16
4.15
4.14
4.13
4.13
4.12
4.10
4.09
4.08
4.07
4.06
4.05
4.03
3.88
3.87
3.86
3.85
3.05
3.03
3.02
3.00
2.98
2.48
2.48
2.47
2.46
2.44
2.43
2.42
1.32
1.30
1.28
1.21
1.19
1.17
¹H NMR 400 MHz, CDCl₃
O
O
F
F
Br
3na
2.01
2.00
4.13
1.01
1.04
1.05
3.26
3.12
f1 (ppm)

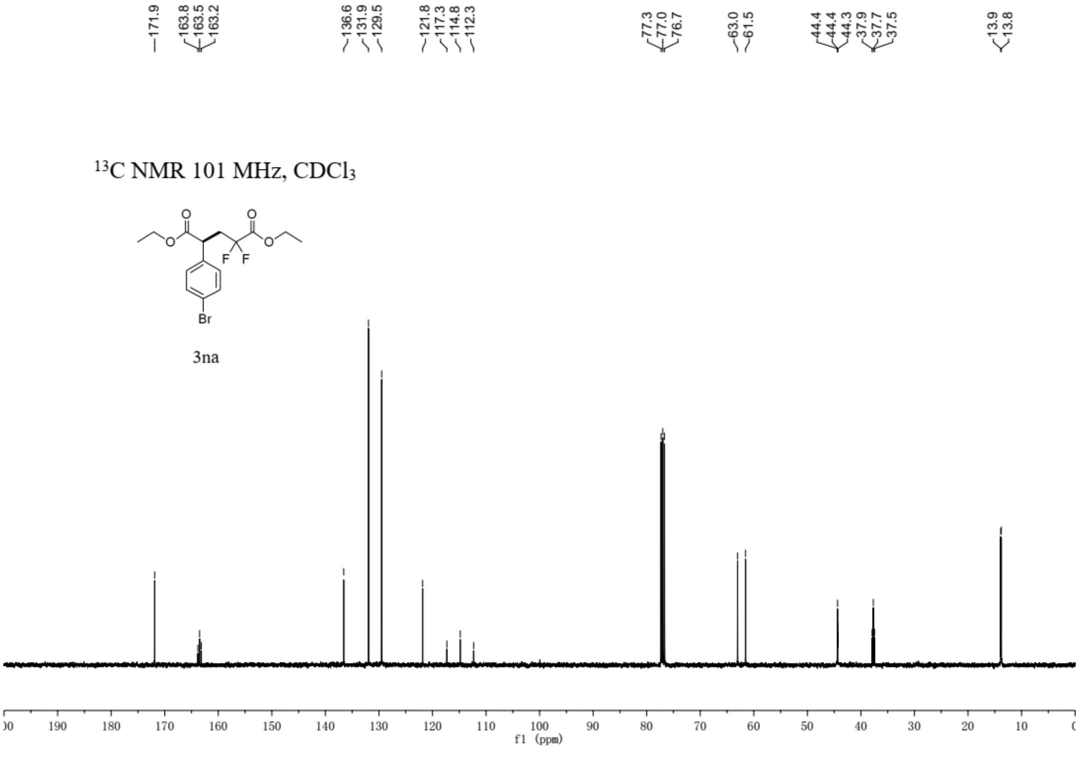
171.9
163.8
163.5
163.2
136.6
131.9
129.5
121.8
117.3
114.8
112.3
77.3
77.0
76.7
63.0
61.5
44.4
44.4
44.3
37.9
37.7
37.5
13.9
13.8
¹³C NMR 101 MHz, CDCl₃
O
O
F
F
Br
3na
f1 (ppm)

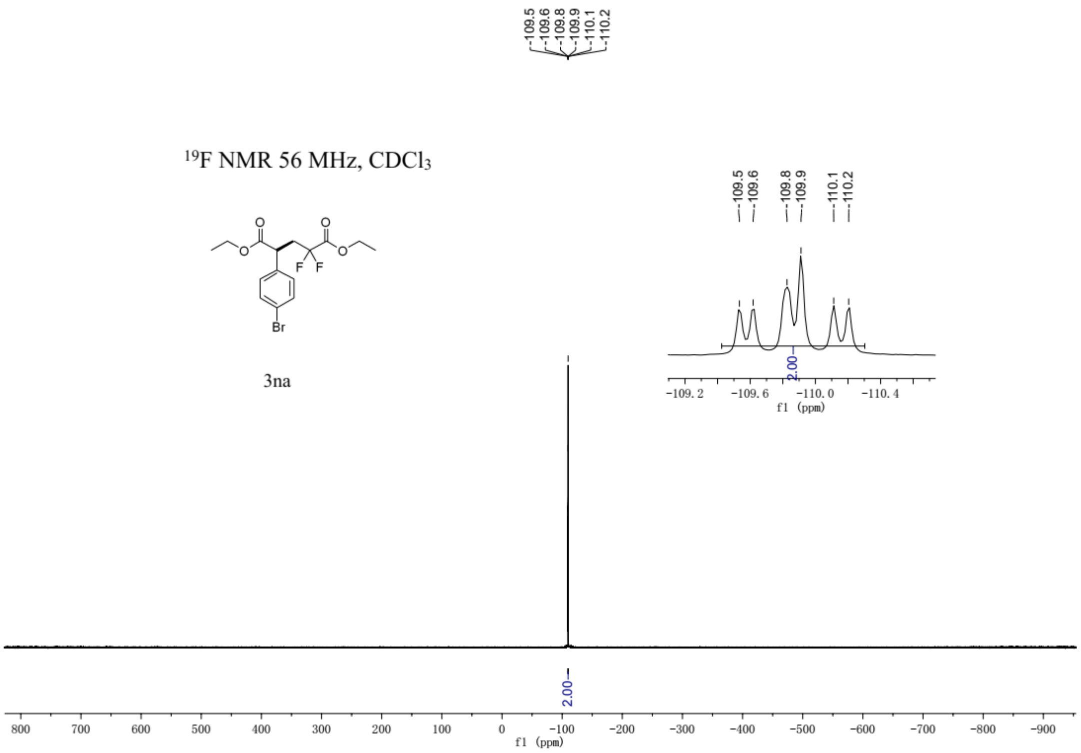
-109.5
-109.6
-109.8
-109.9
-110.1
-110.2
19F NMR 56 MHz, CDCl3
3na
2.00
800
700
600
500
400
300
200
100
0
-100
-200
-300
-400
-500
-600
-700
-800
-900
f1 (ppm)
-109.2
-109.6
-110.0
-110.4

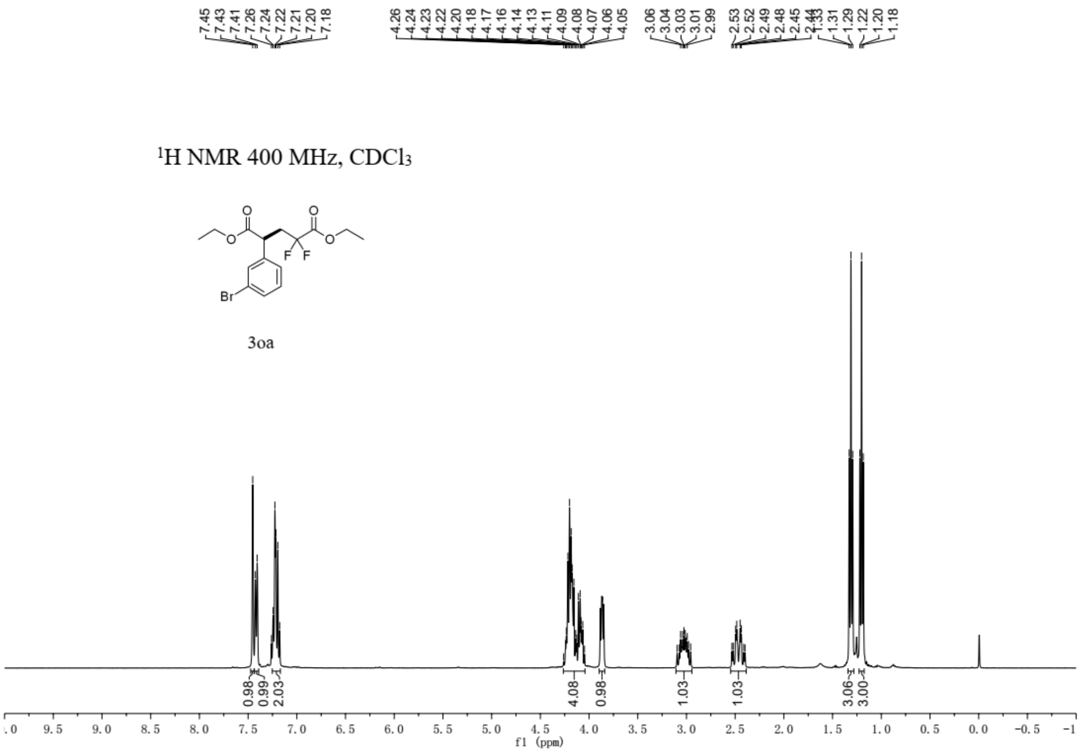

^{1}H NMR 400 MHz, $CDCl_3$
3oa

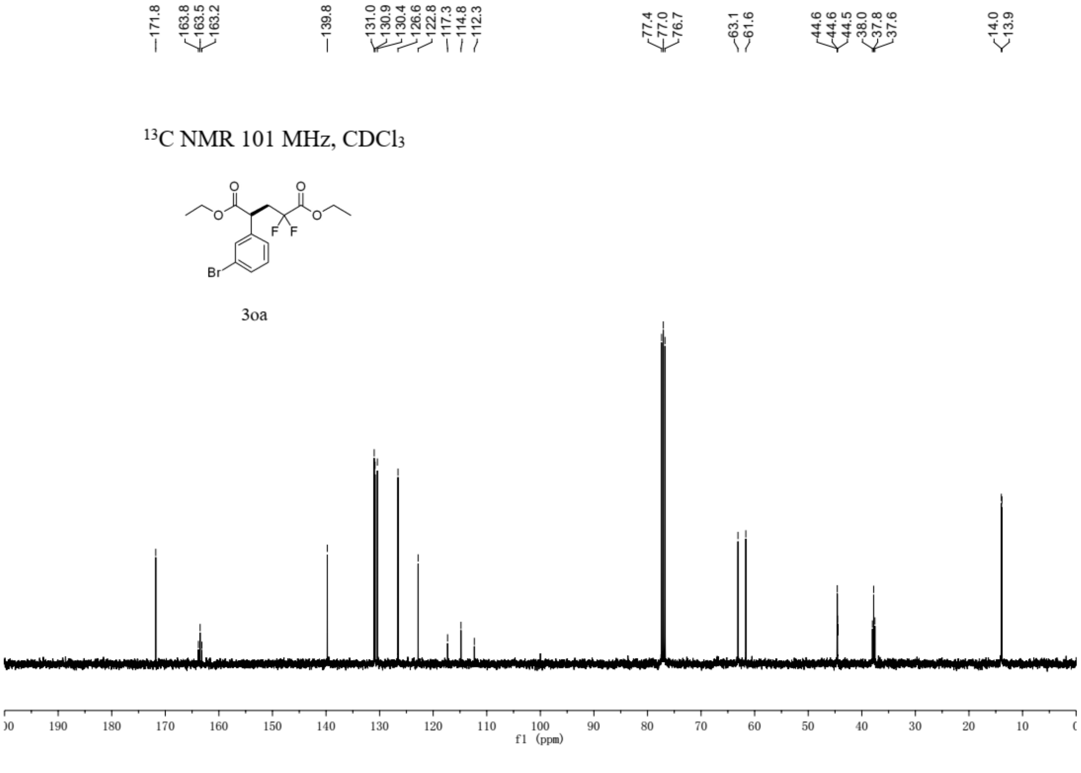

^{13}C NMR 101 MHz, $CDCl_3$
3oa

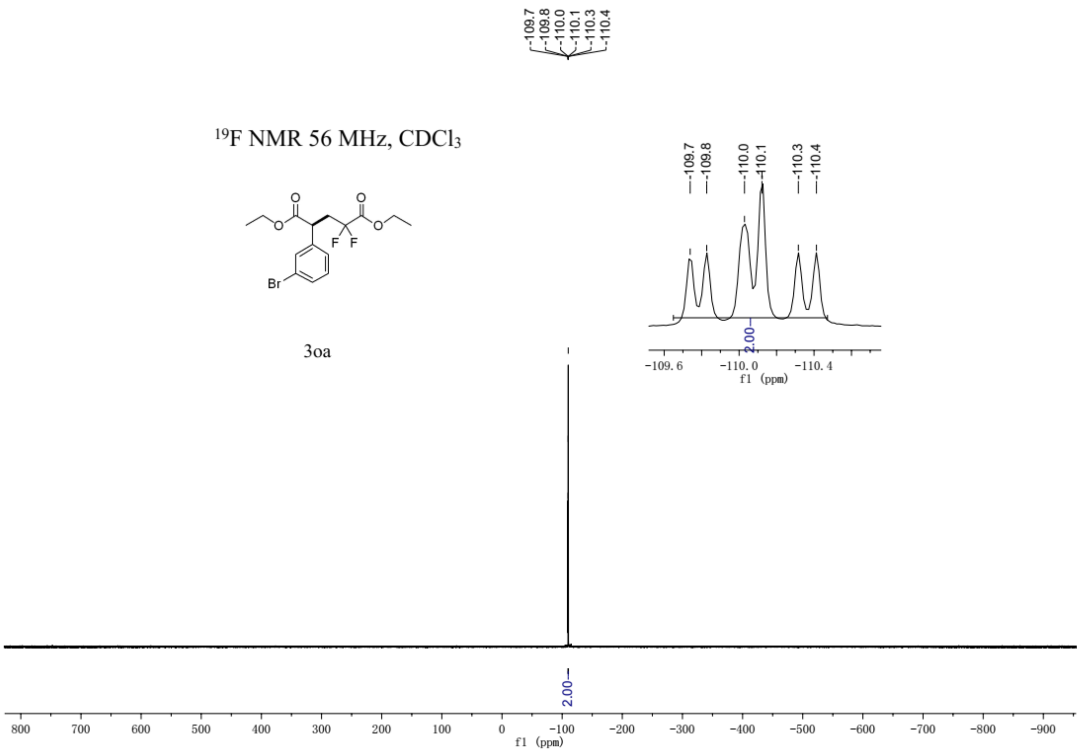
-109.7
-109.8
-110.0
-110.1
-110.3
-110.4
^{19}F NMR 56 MHz, $CDCl_3$
Br
F F
3oa
2.00
-109.6
-110.0
-110.4
f1 (ppm)
800 700 600 500 400 300 200 100 0 -100 -200 -300 -400 -500 -600 -700 -800 -900

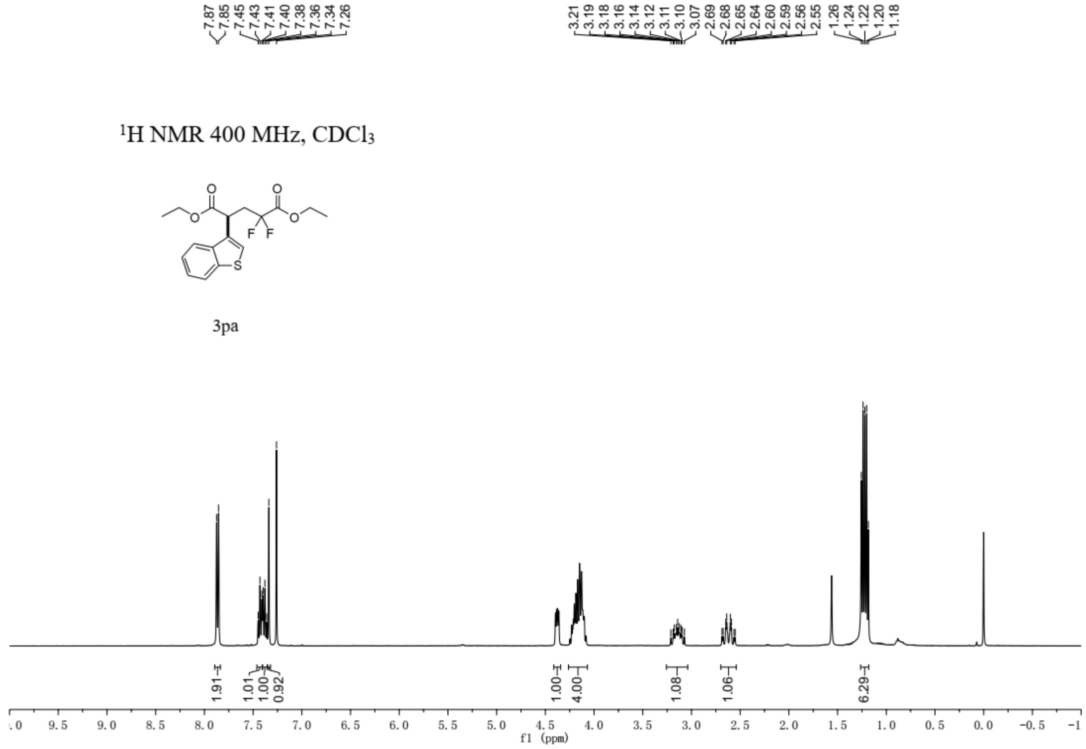
^{1}H NMR 400 MHz, $CDCl_3$
3pa
f1 (ppm)

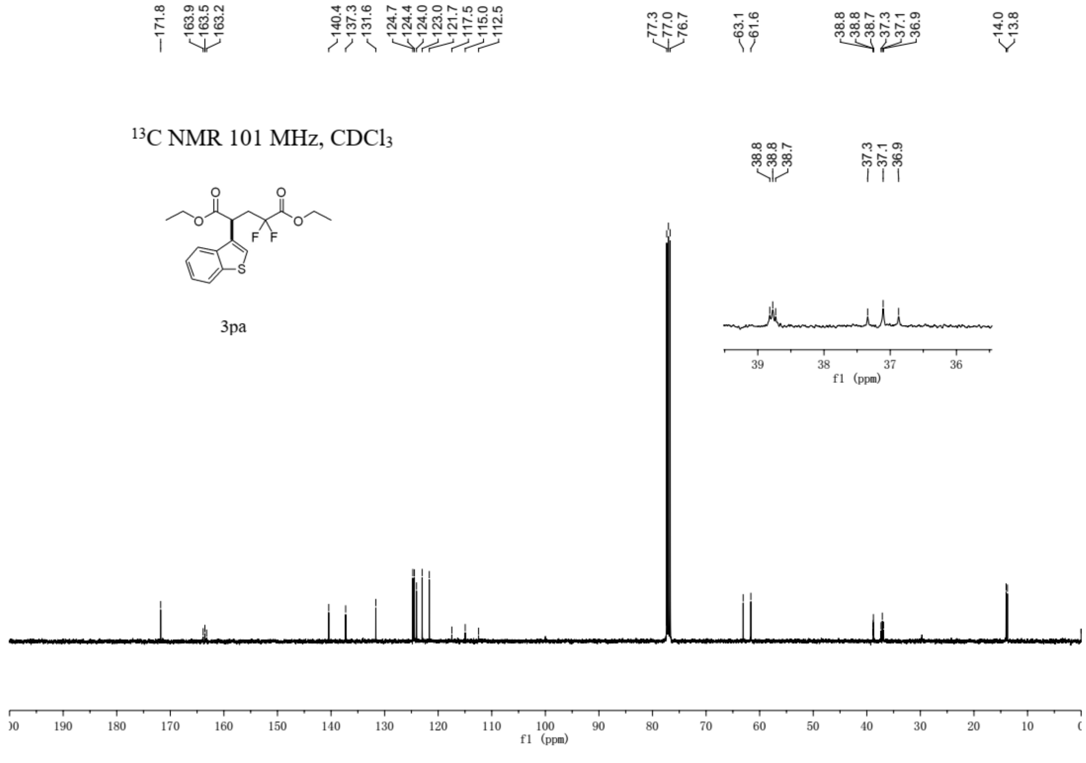
^{13}C NMR 101 MHz, $CDCl_3$
3pa
f1 (ppm)

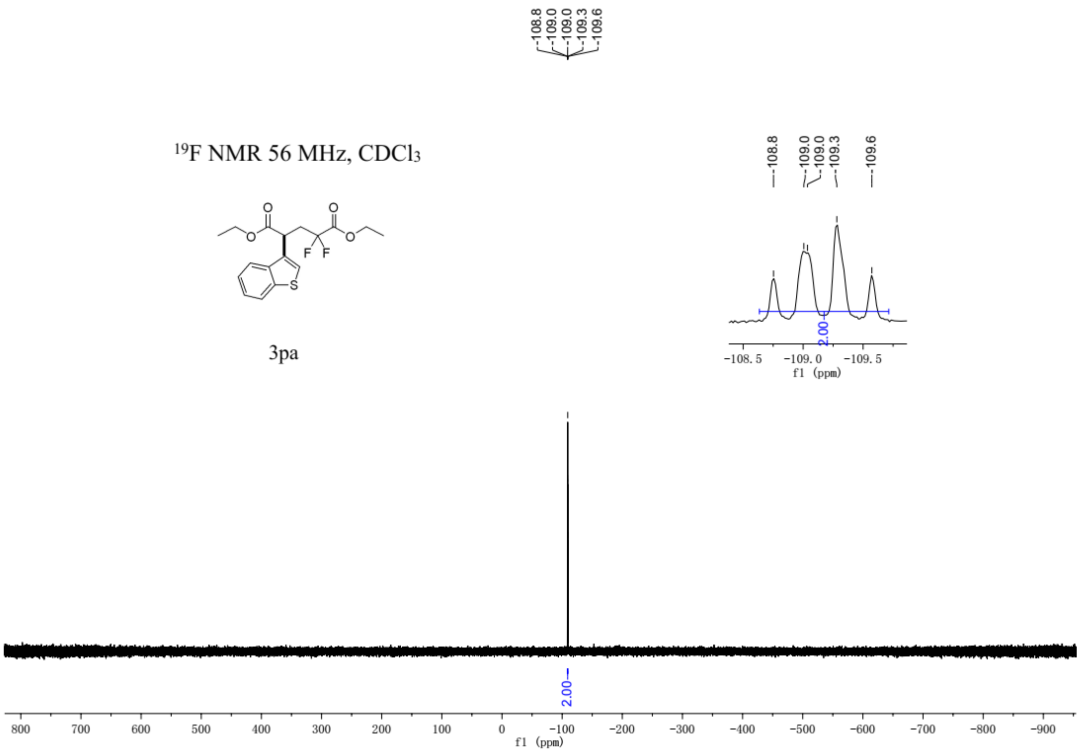
-108.8
-109.0
-109.0
-109.3
-109.6
¹⁹F NMR 56 MHz, CDCl₃
3pa
2.00
−108.5
−109.0
−109.5
f1 (ppm)
800
700
600
500
400
300
200
100
0
−100
−200
−300
−400
−500
−600
−700
−800
−900

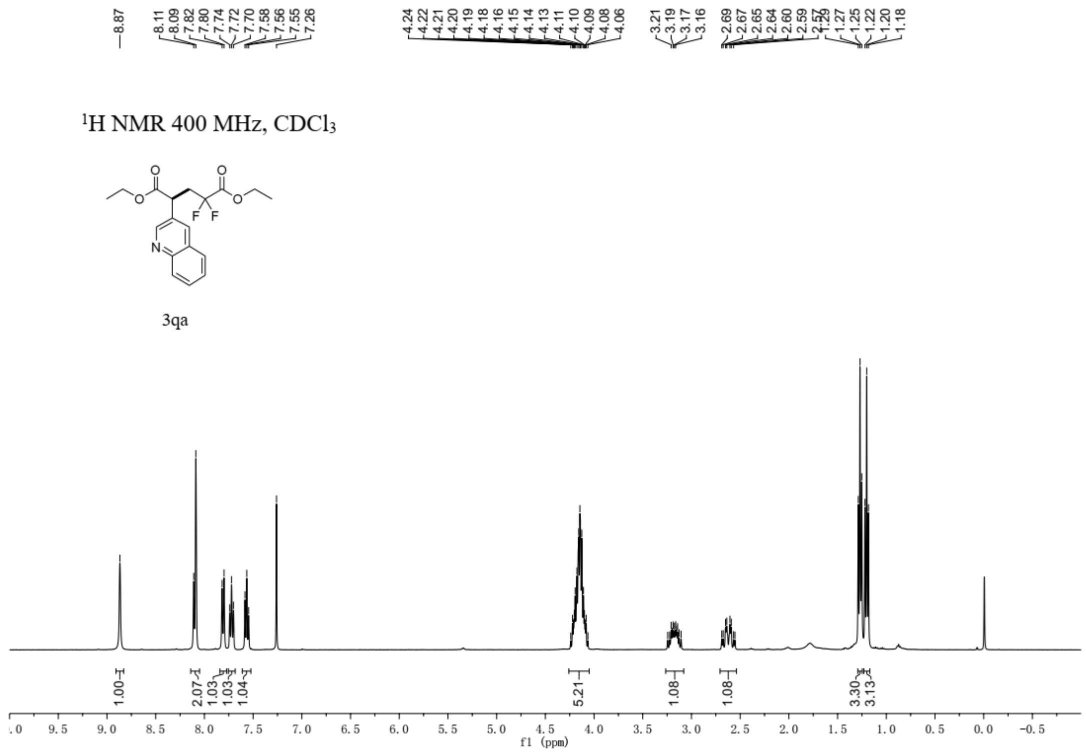

1H NMR 400 MHz, CDCl3
3qa
f1 (ppm)

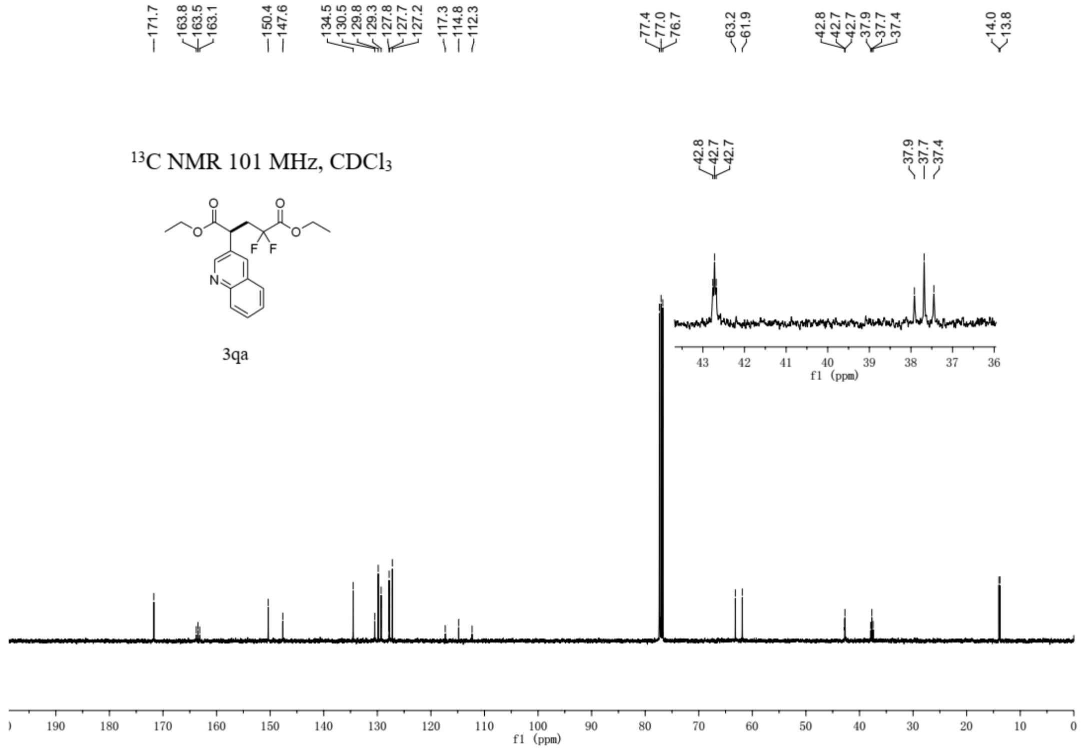

13C NMR 101 MHz, CDCl3
3qa
f1 (ppm)

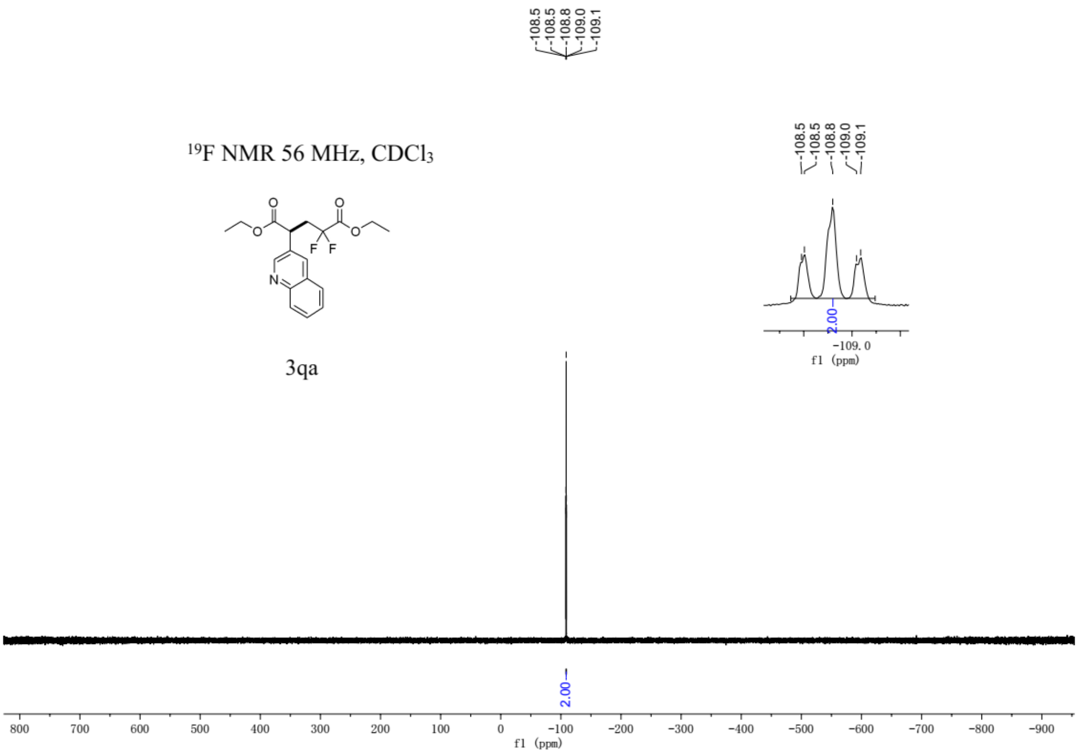

19F NMR 56 MHz, CDCl3
3qa
-108.5
-108.5
-108.8
-109.0
-109.1
2.00
-109.0
f1 (ppm)
800
700
600
500
400
300
200
100
0
-100
-200
-300
-400
-500
-600
-700
-800
-900

^{1}H NMR 400 MHz, $CDCl_3$

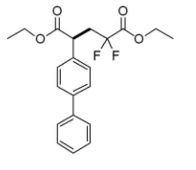

3ra

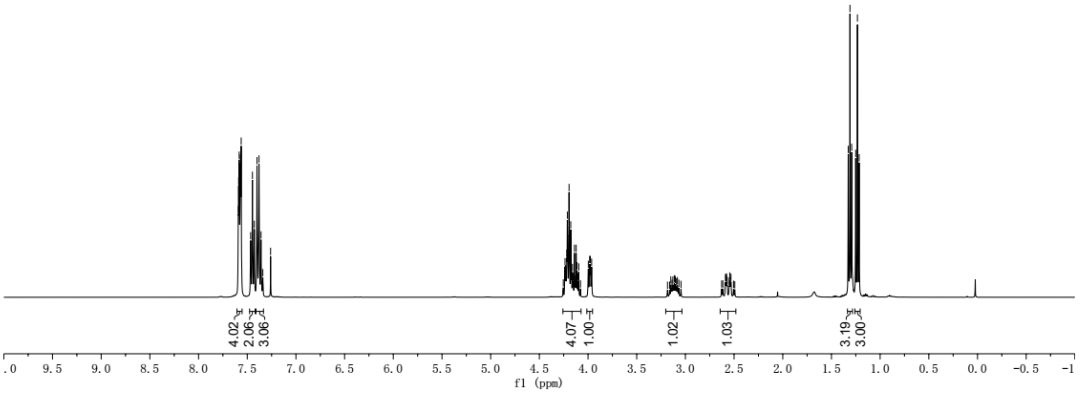

^{13}C NMR 101 MHz, $CDCl_3$

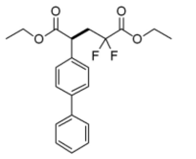

3ra

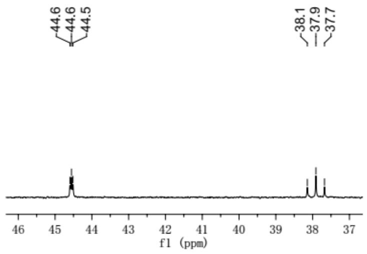

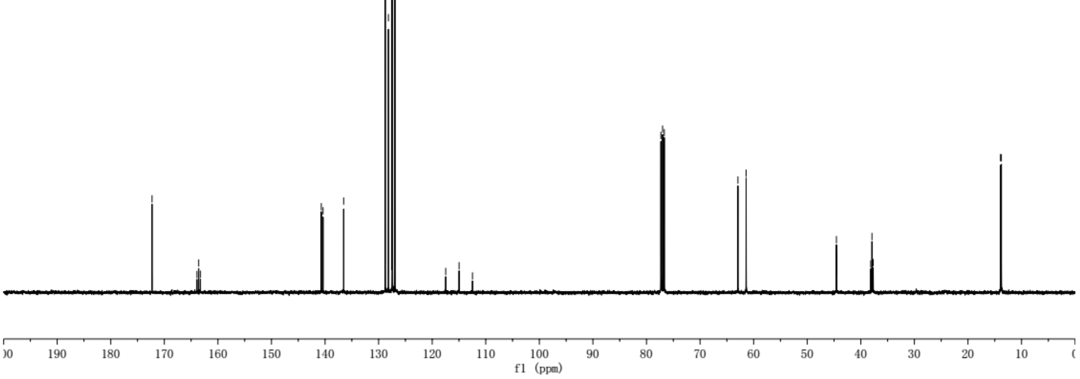

^{19}F NMR 56 MHz, CDCl$_3$

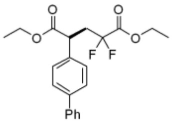

3ra

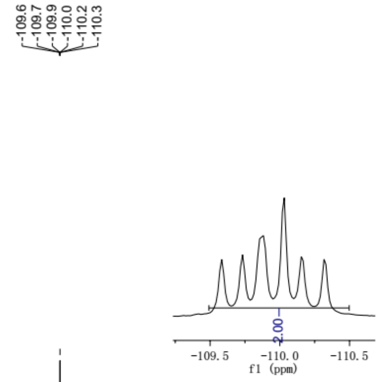

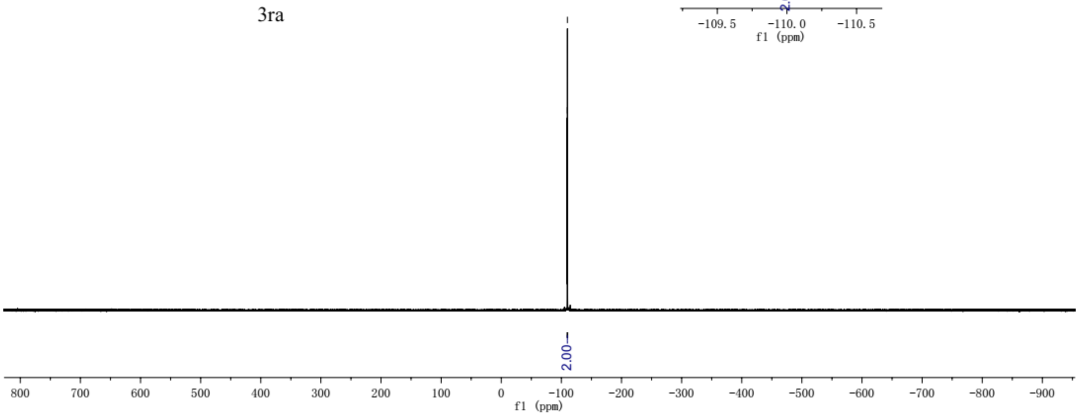

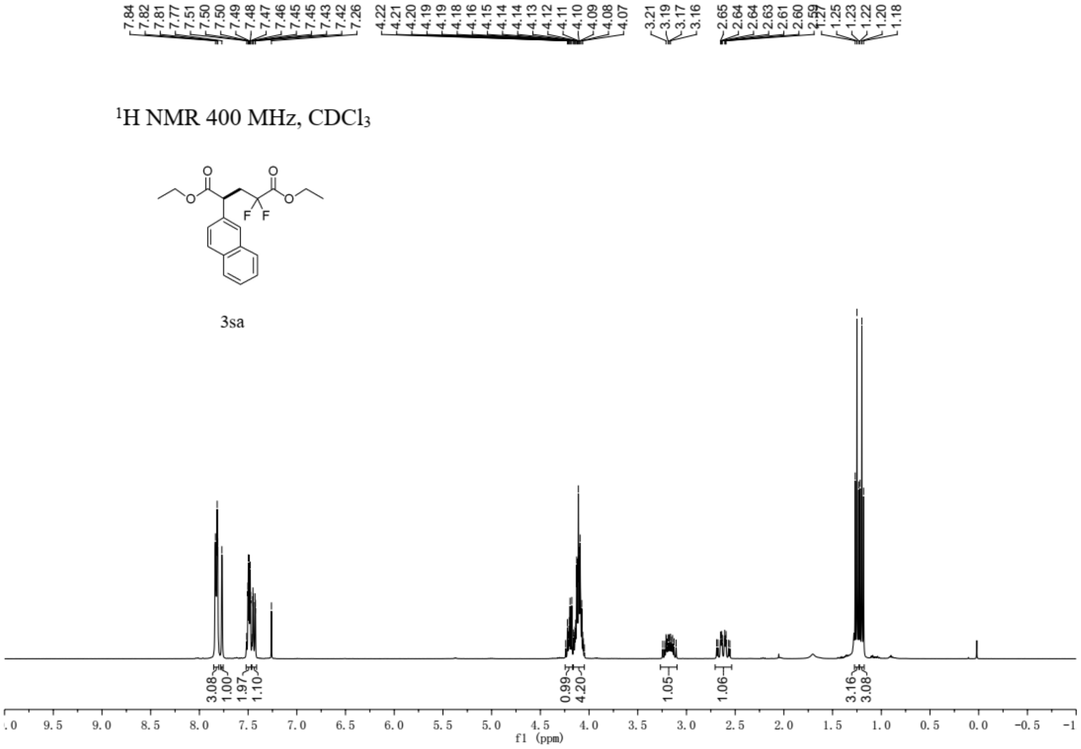

^{1}H NMR 400 MHz, $CDCl_3$

3sa

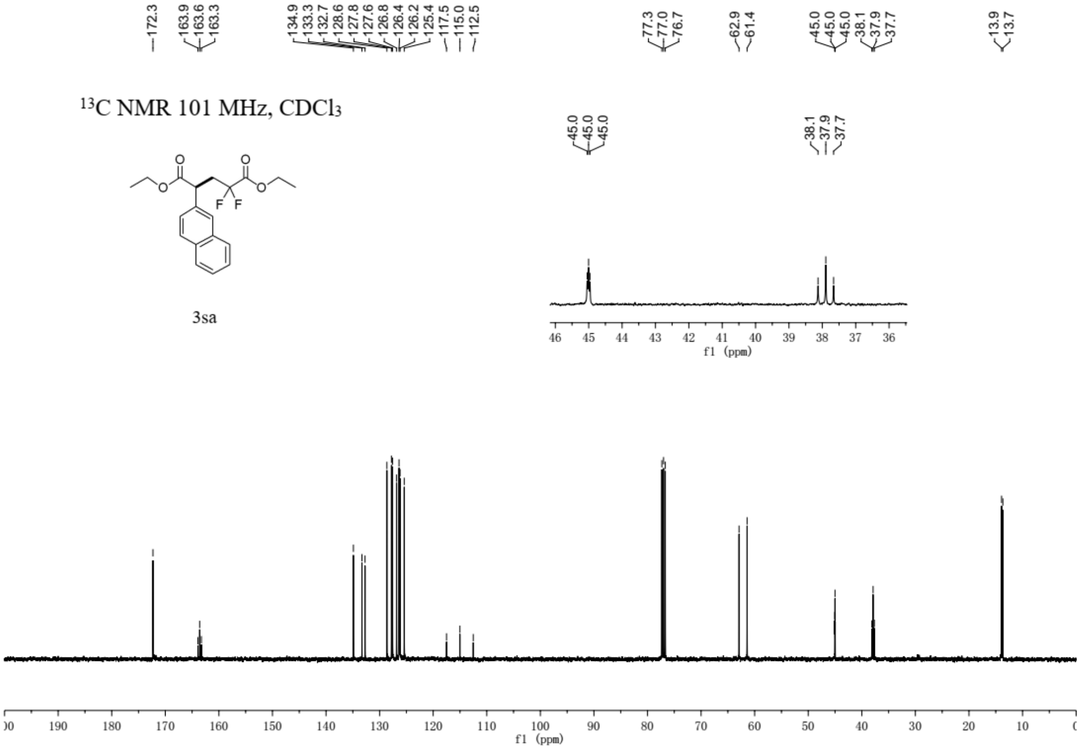

^{13}C NMR 101 MHz, $CDCl_3$

3sa

^{19}F NMR 56 MHz, $CDCl_3$

3sa

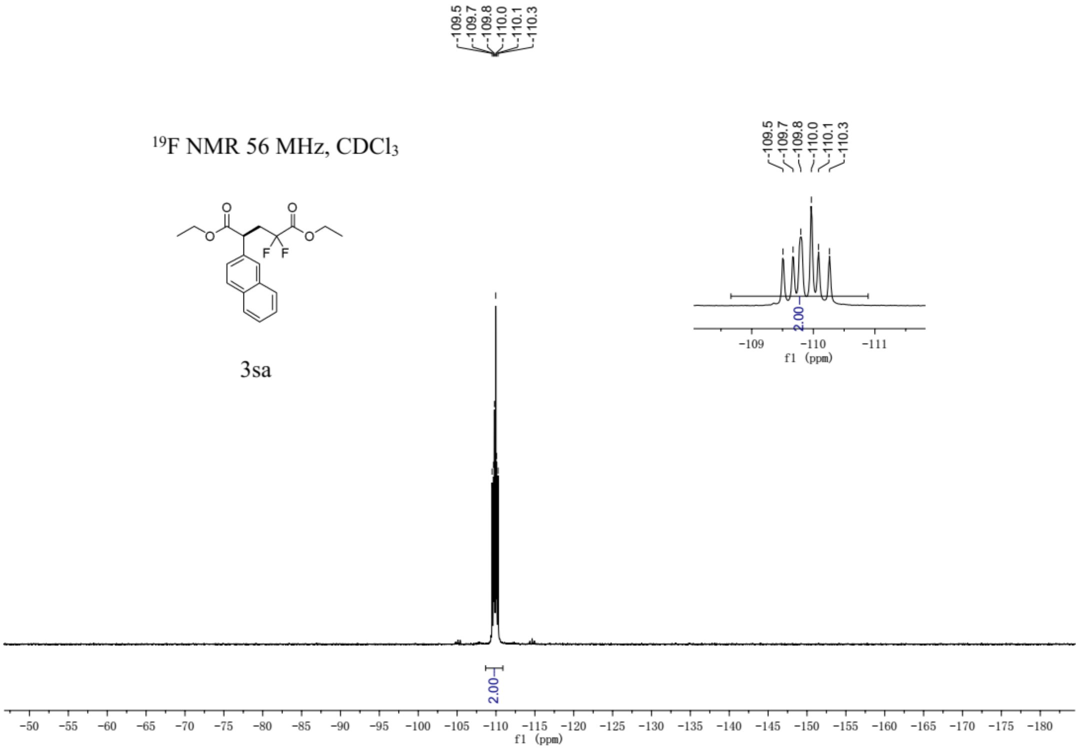

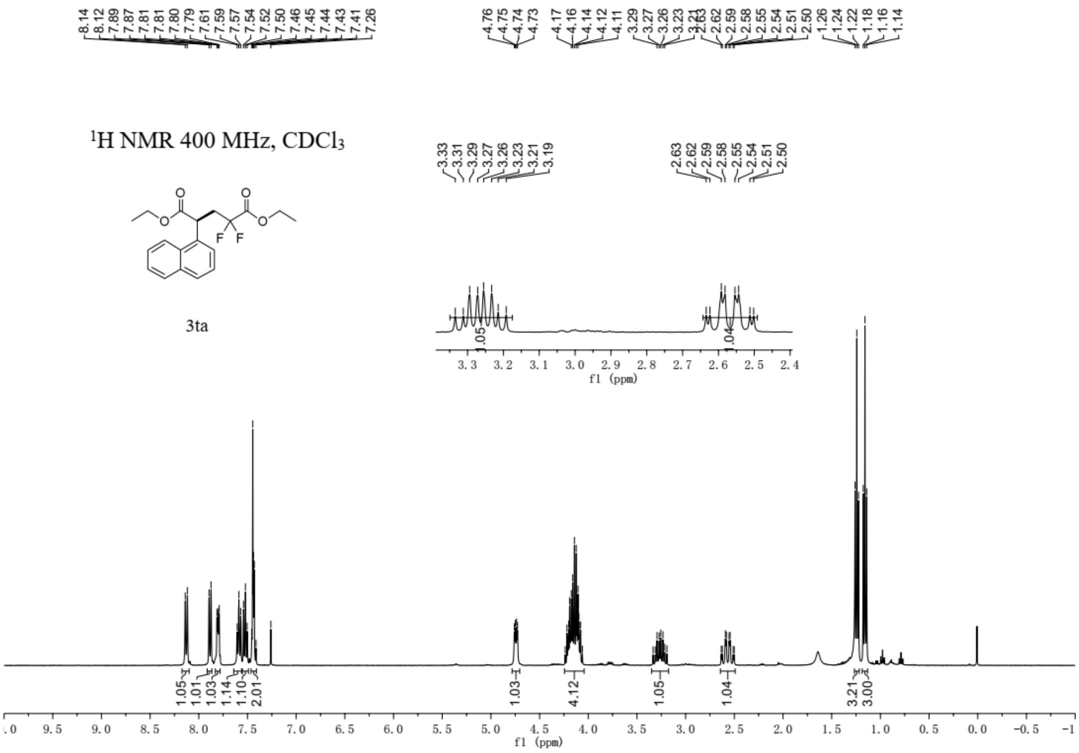
^{1}H NMR 400 MHz, CDCl$_3$
3ta

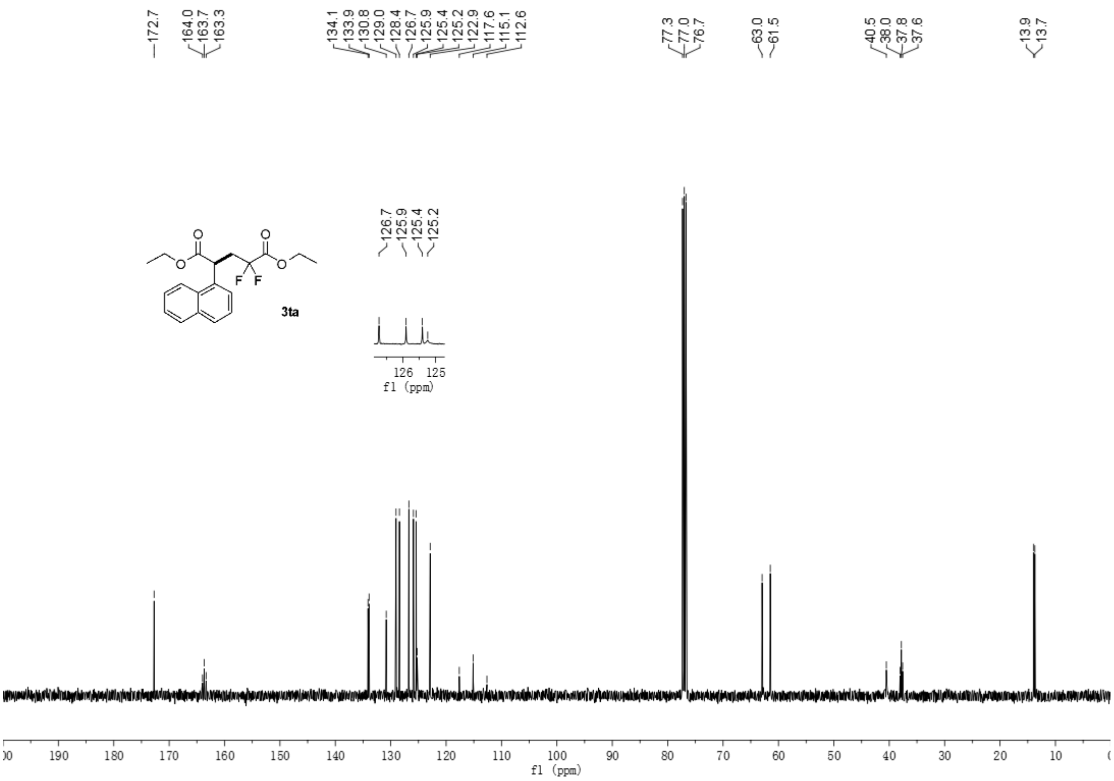
3ta

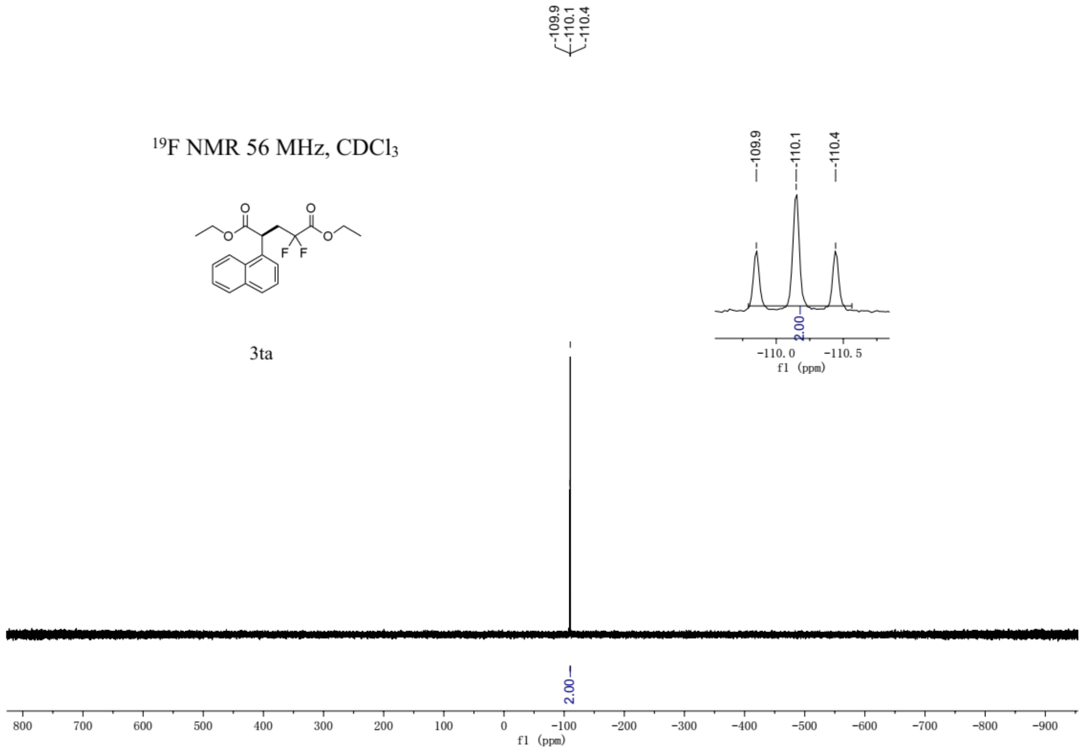

-109.9
-110.1
-110.4
19F NMR 56 MHz, CDCl3
3ta
2.00
f1 (ppm)

^1H NMR 400 MHz, $CDCl_3$

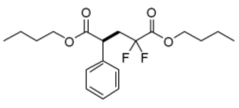

3ab

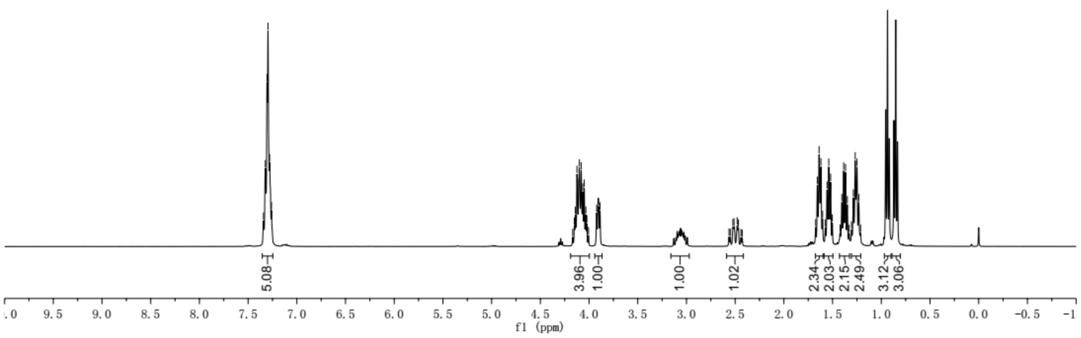

^{13}C NMR 101 MHz, $CDCl_3$

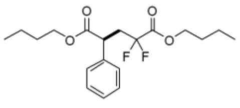

3ab

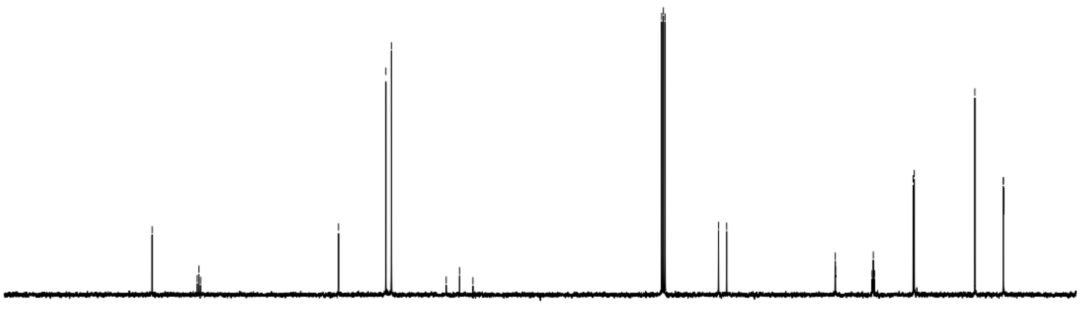

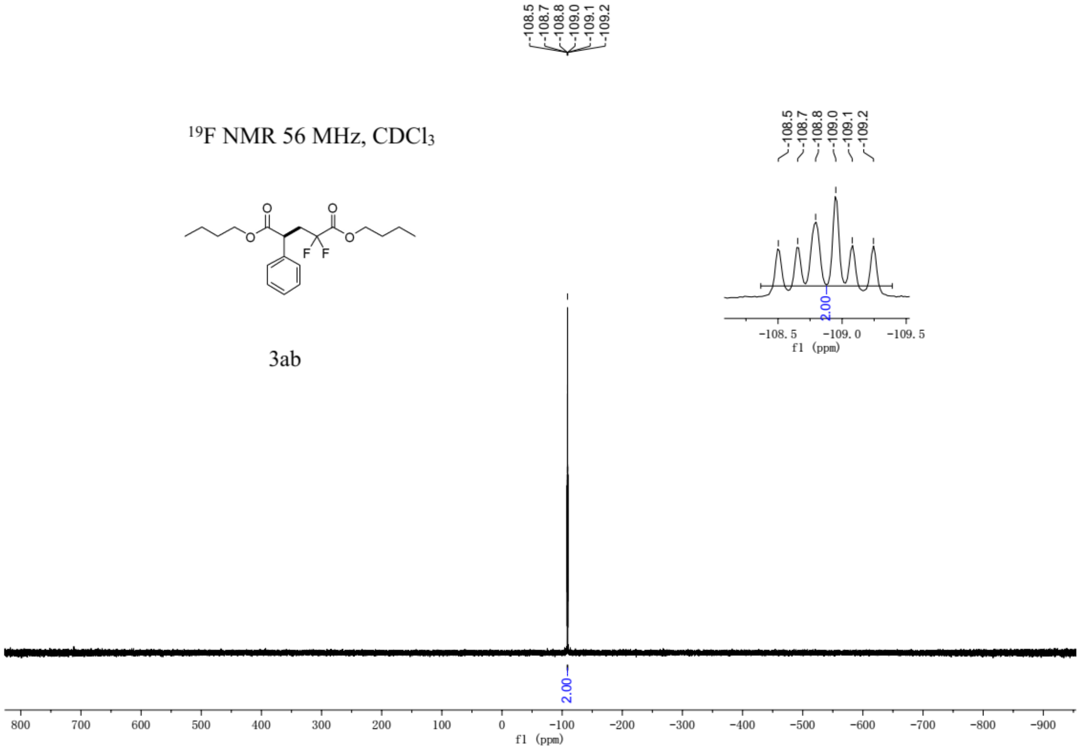

-108.5
-108.7
-108.8
-109.0
-109.1
-109.2
^{19}F NMR 56 MHz, CDCl$_3$
F
F
O
3ab
2.00
-108.5
-109.0
-109.5
f1 (ppm)
800
700
600
500
400
300
200
100
0
-100
-200
-300
-400
-500
-600
-700
-800
-900
f1 (ppm)

^{1}H NMR (700 MHz, $CDCl_3$) 3ac

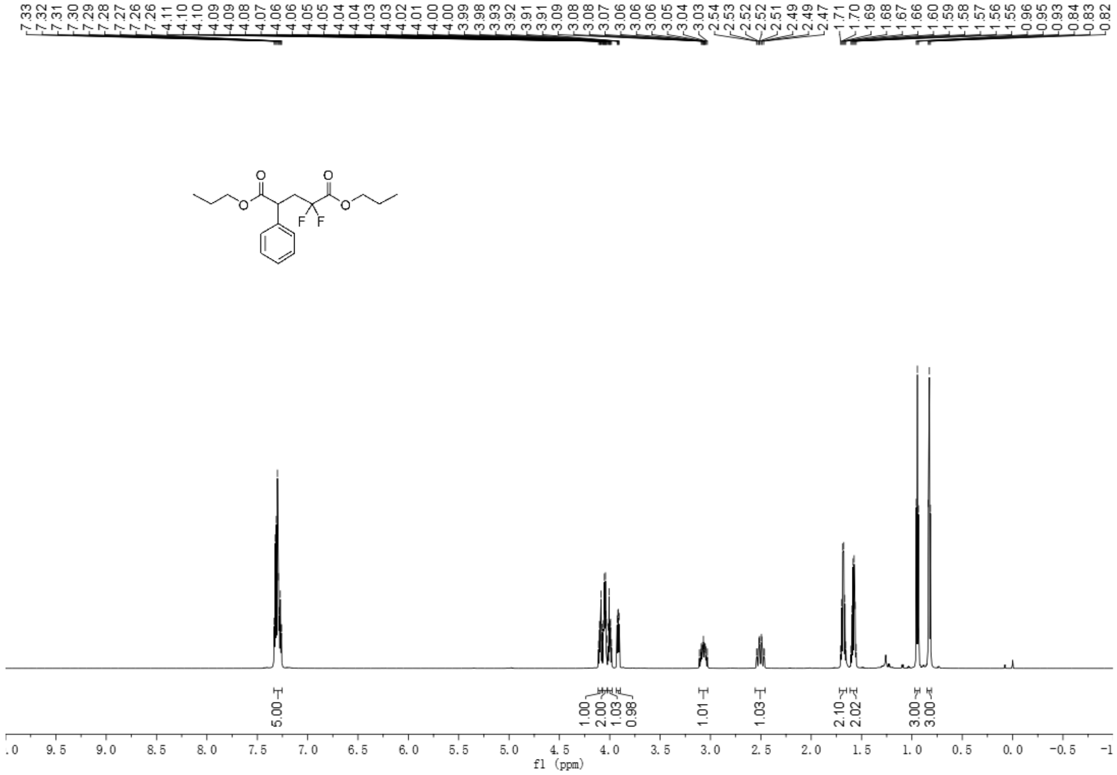

^{13}C NMR (176 MHz, $CDCl_3$) 3ac

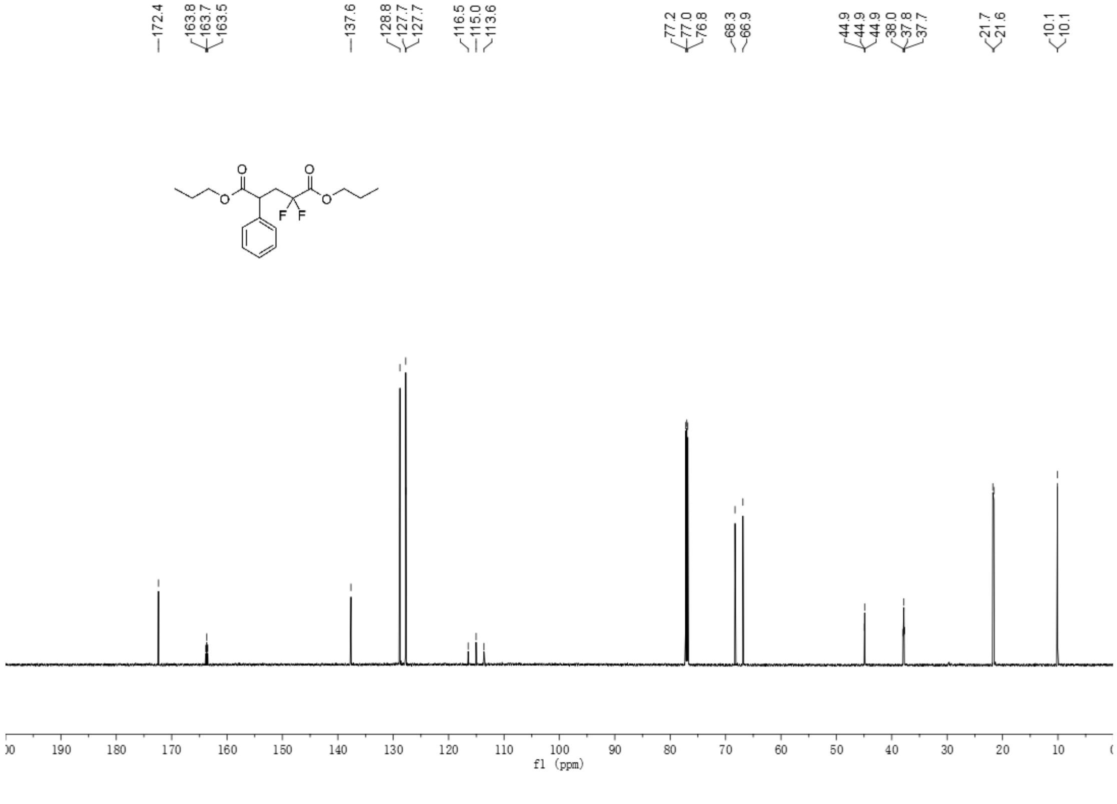

^{19}F NMR (56 MHz, $CDCl_3$) 3ac

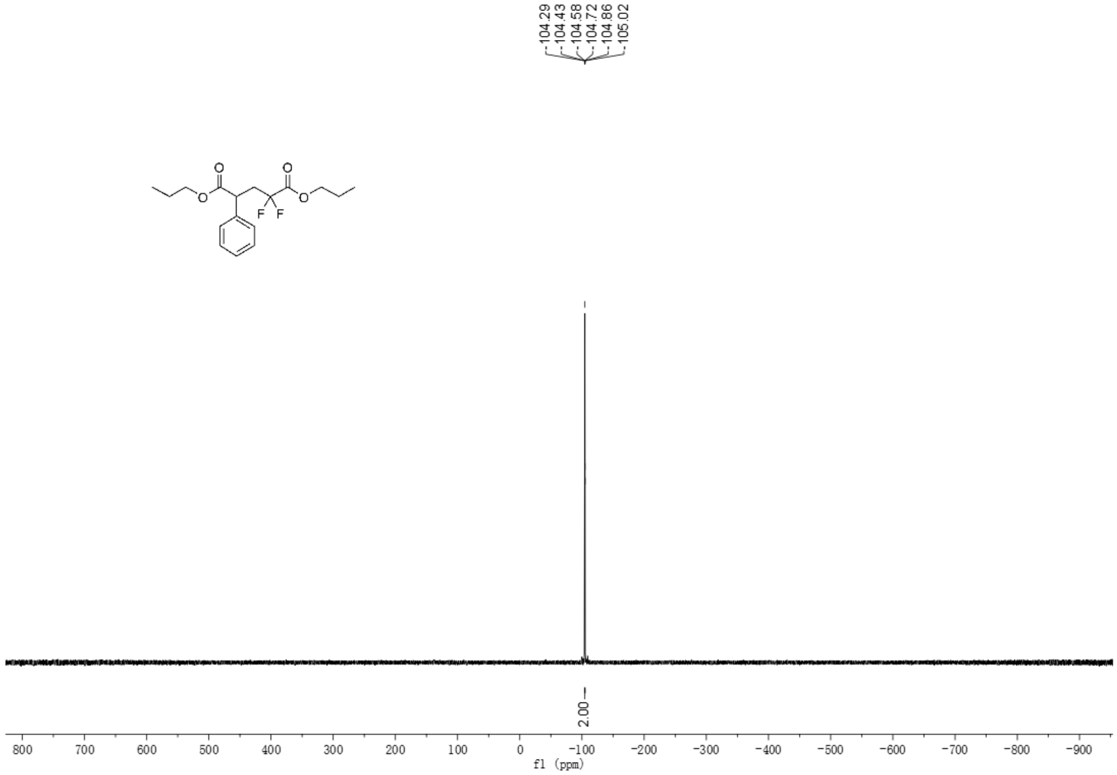

^{1}H NMR (700 MHz, CDCl$_3$) 3ad

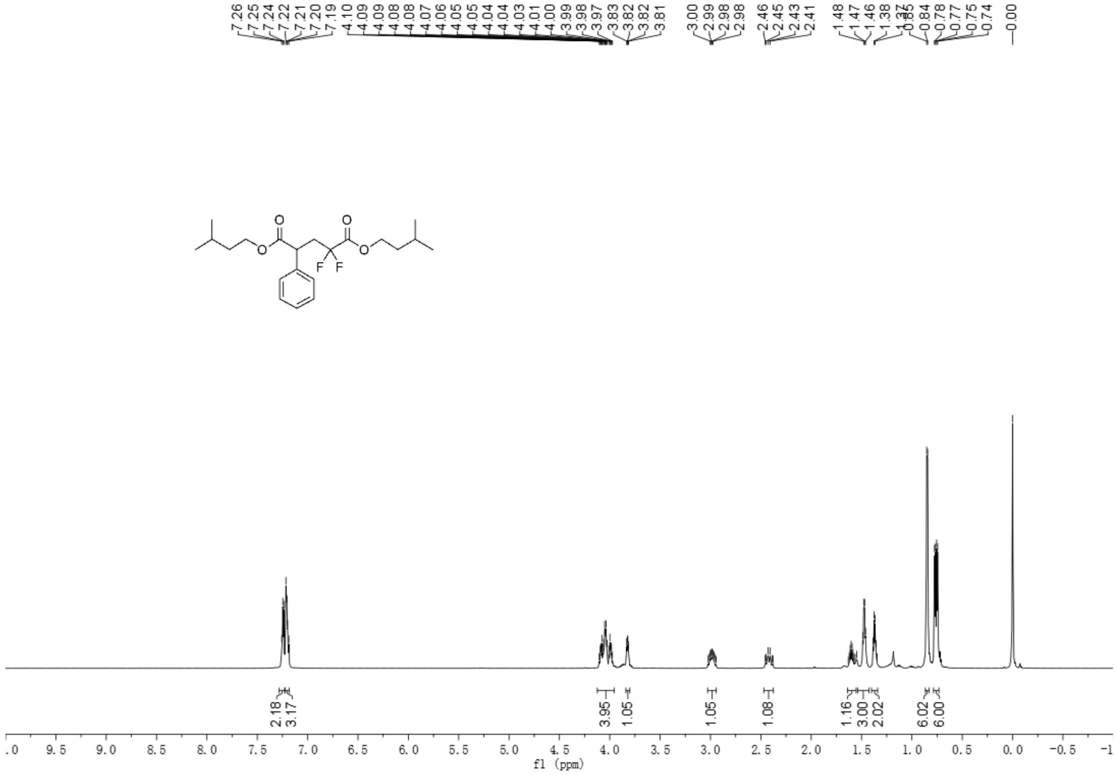

^{13}C NMR (176 MHz, CDCl$_3$) 3ad

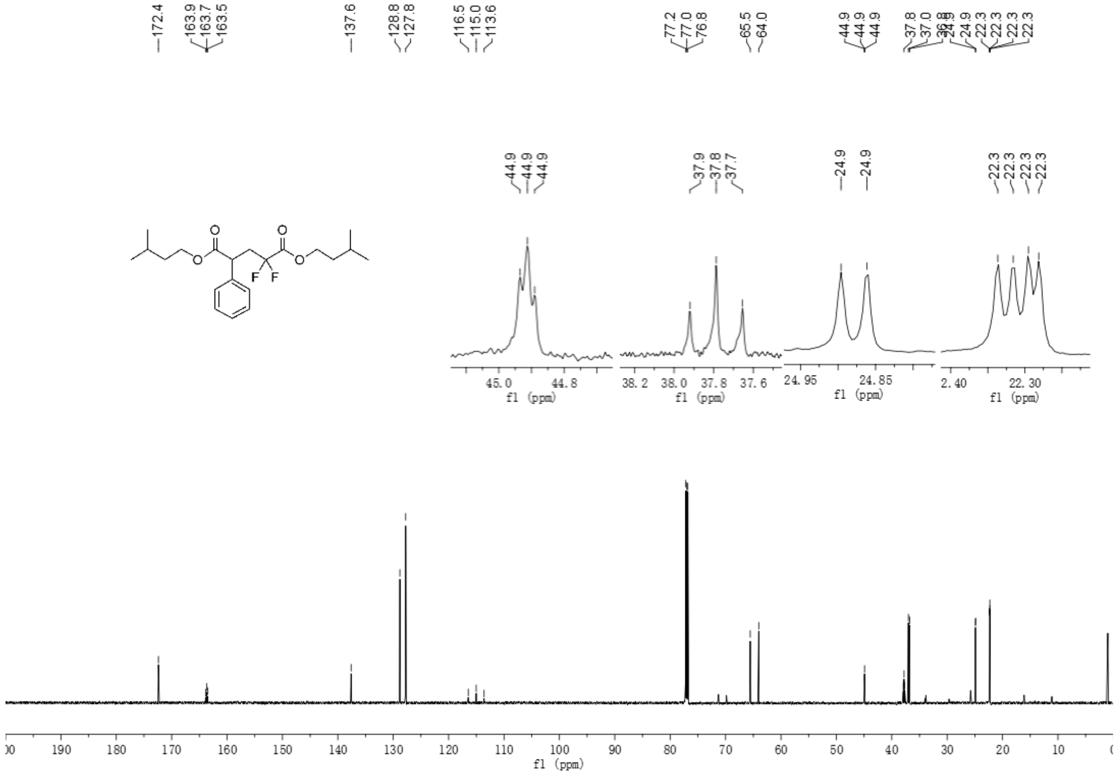

^{19}F NMR (56 MHz, $CDCl_3$) 3ad

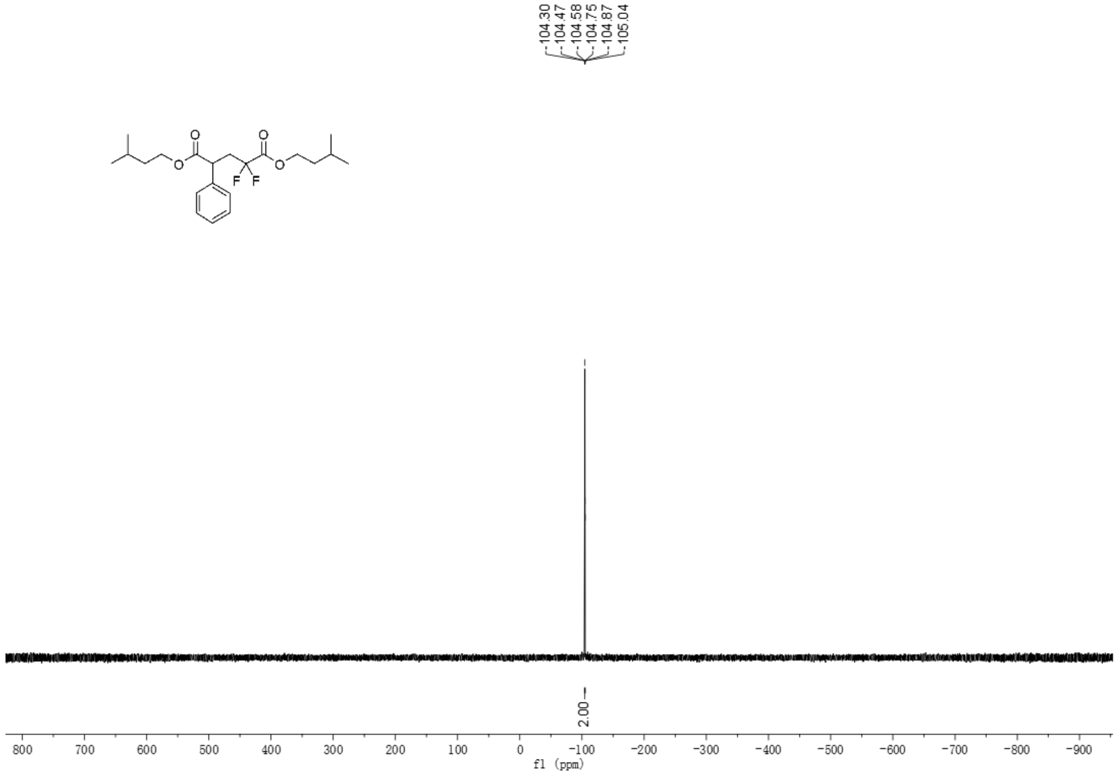

^{1}H NMR (700 MHz, $CDCl_3$) 3ae

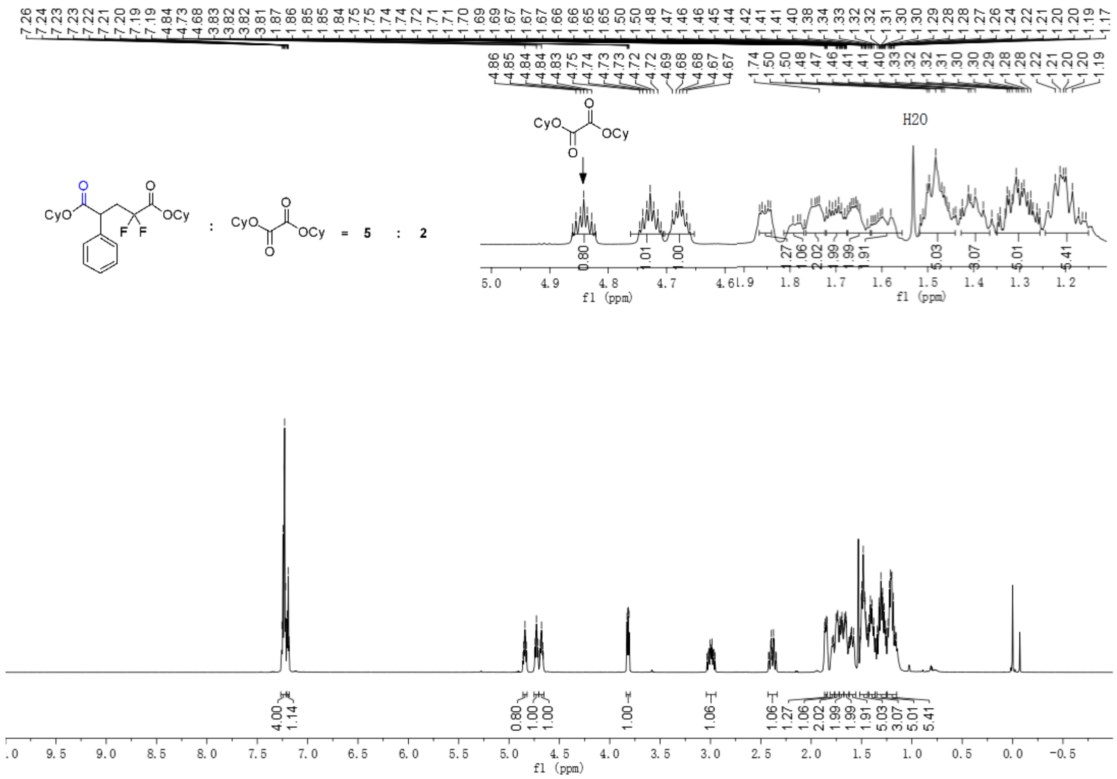

^{13}C NMR (176 MHz, $CDCl_3$) 3ae

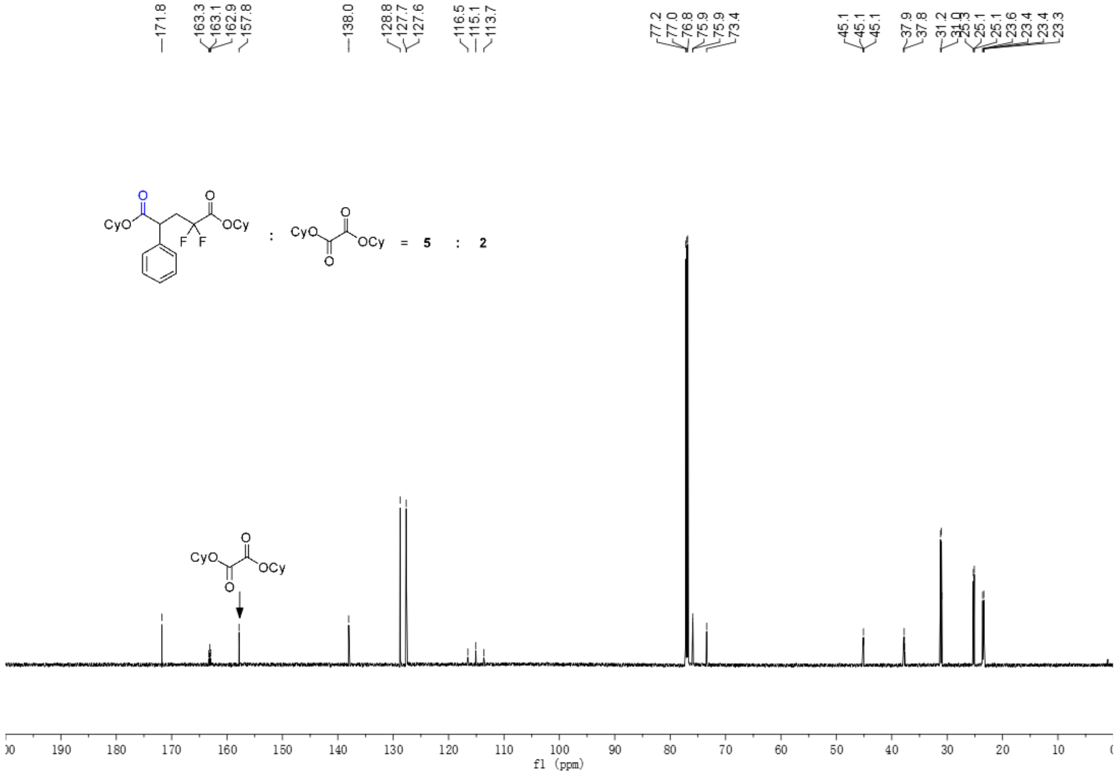

^{19}F NMR (56 MHz, $CDCl_3$) 3ae

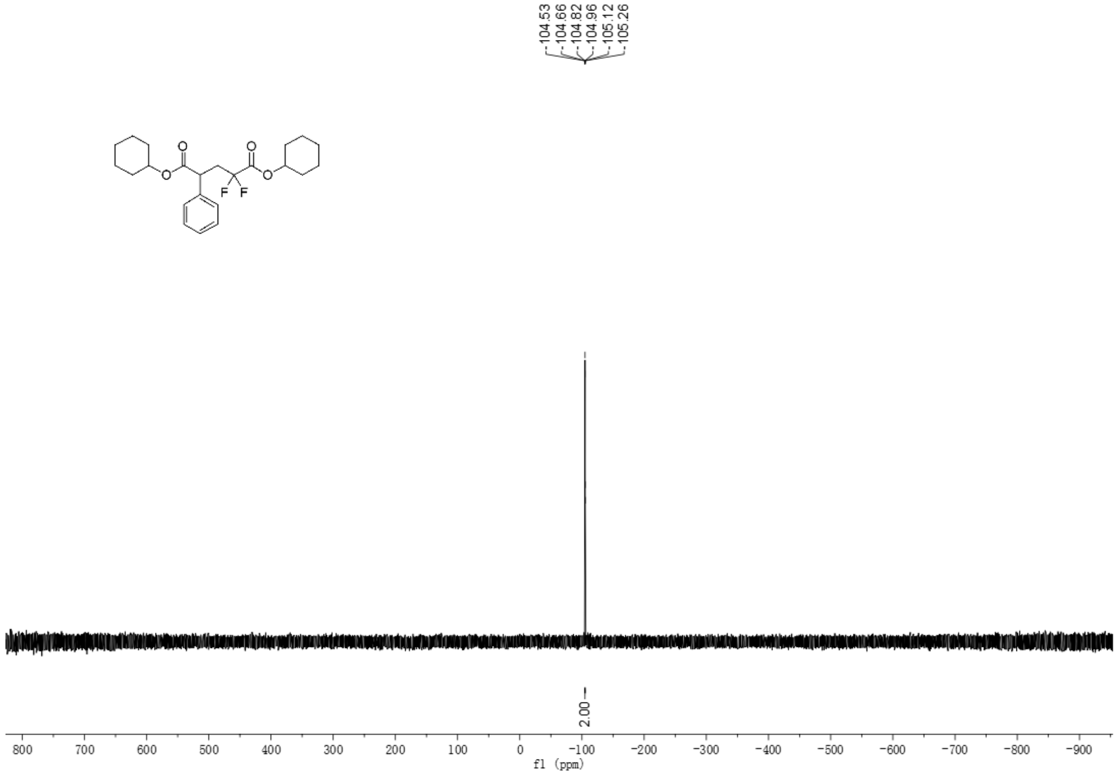

^{1}H NMR (700 MHz, $CDCl_3$) 3af

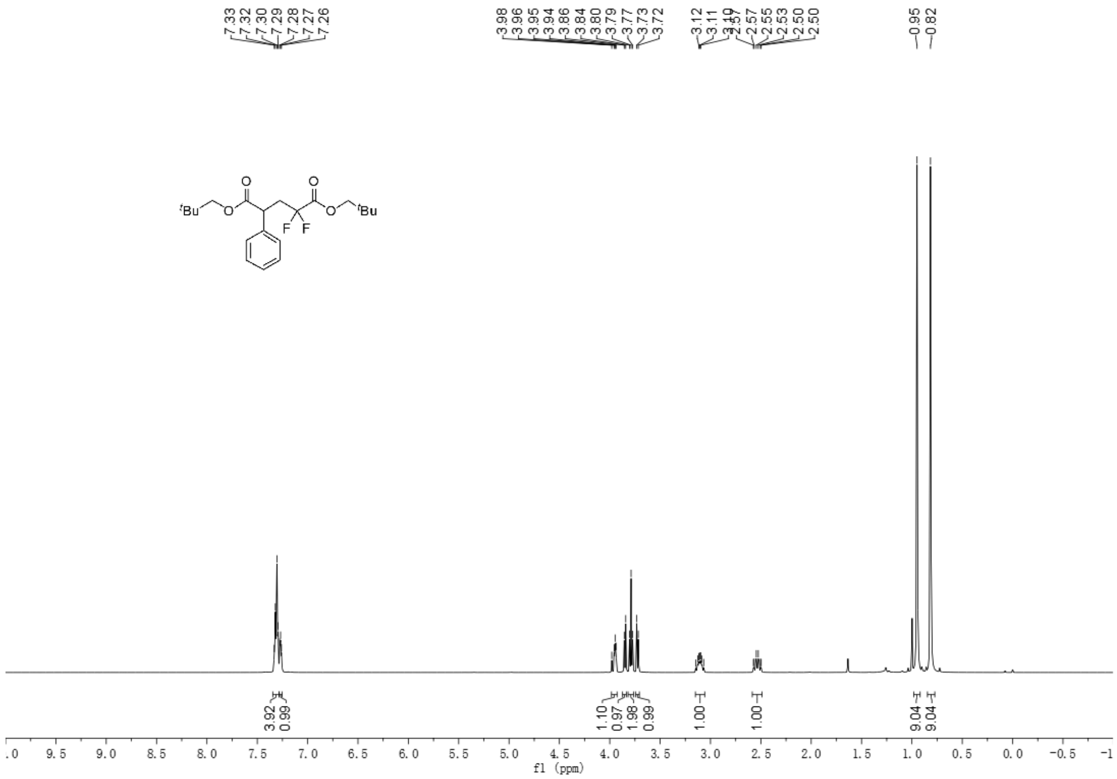

^{13}C NMR (176 MHz, $CDCl_3$) 3af

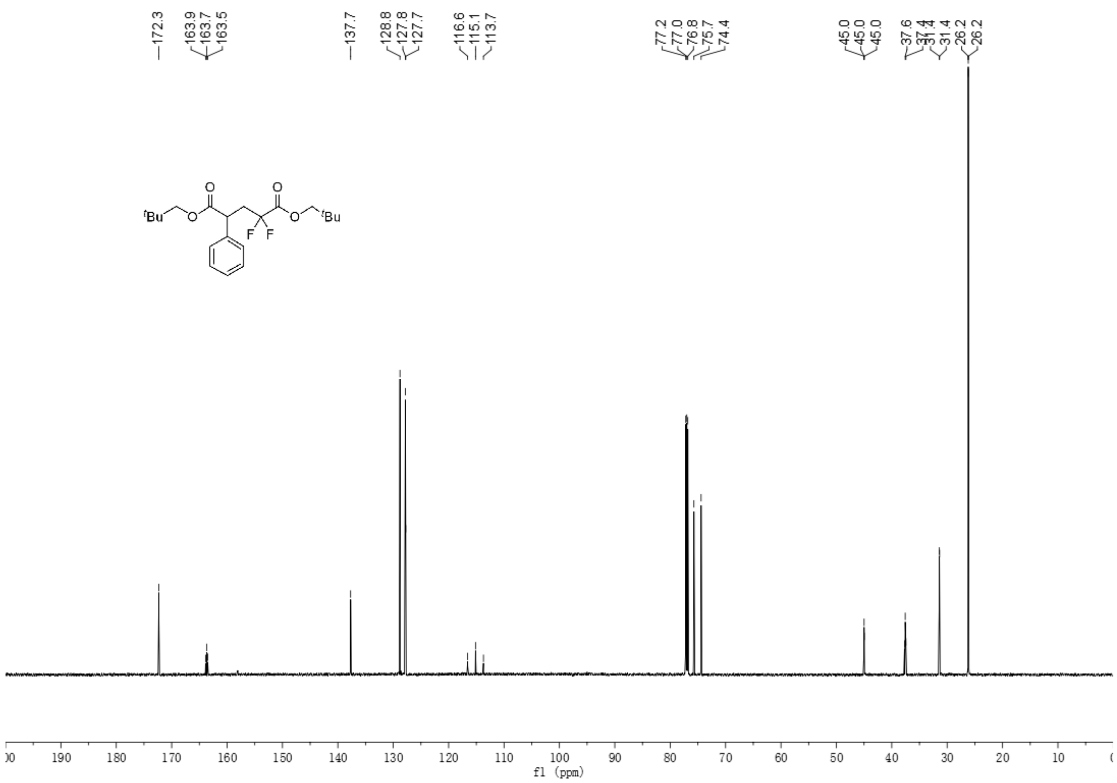

^{19}F NMR (56 MHz, CDCl$_3$) 3af

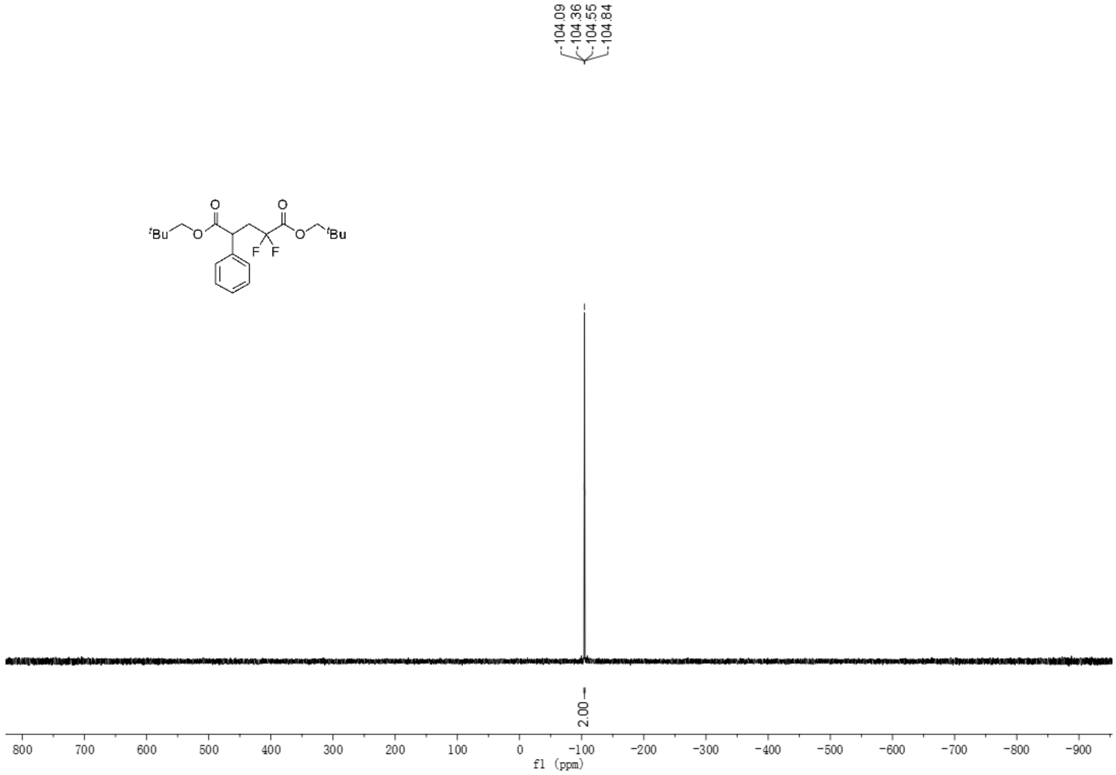

^{1}H NMR (700 MHz, CDCl$_3$) 3ag

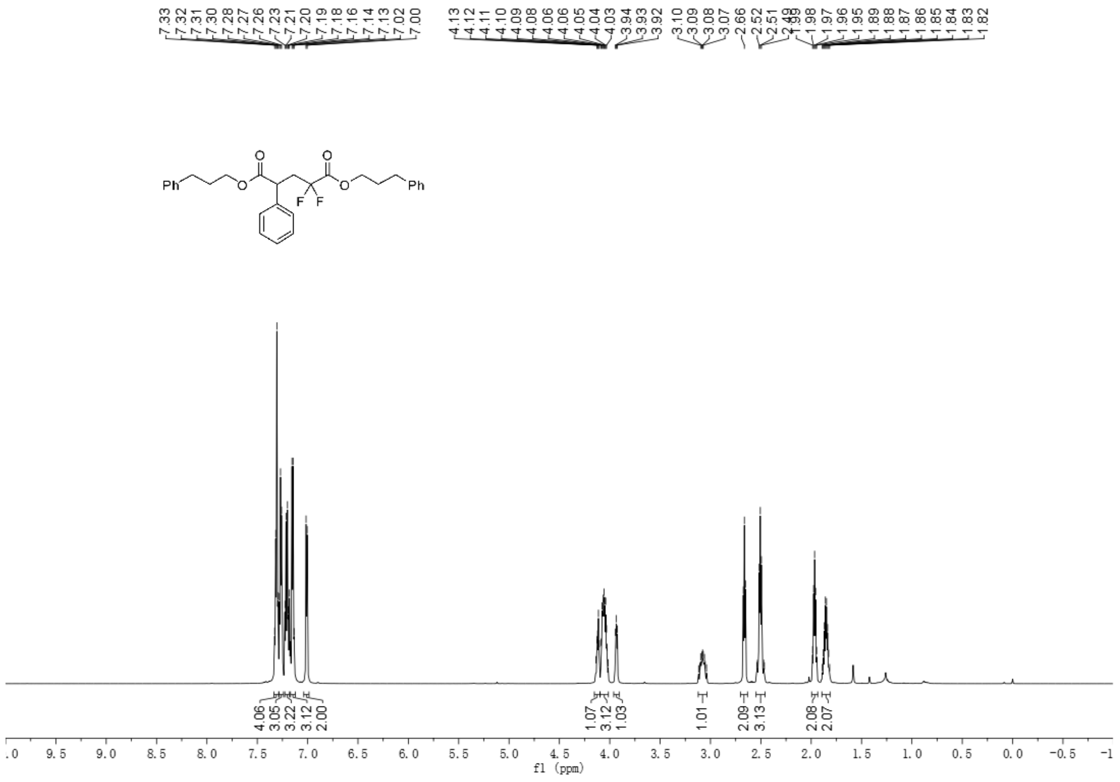

^{13}C NMR (176 MHz, CDCl$_3$) 3ag

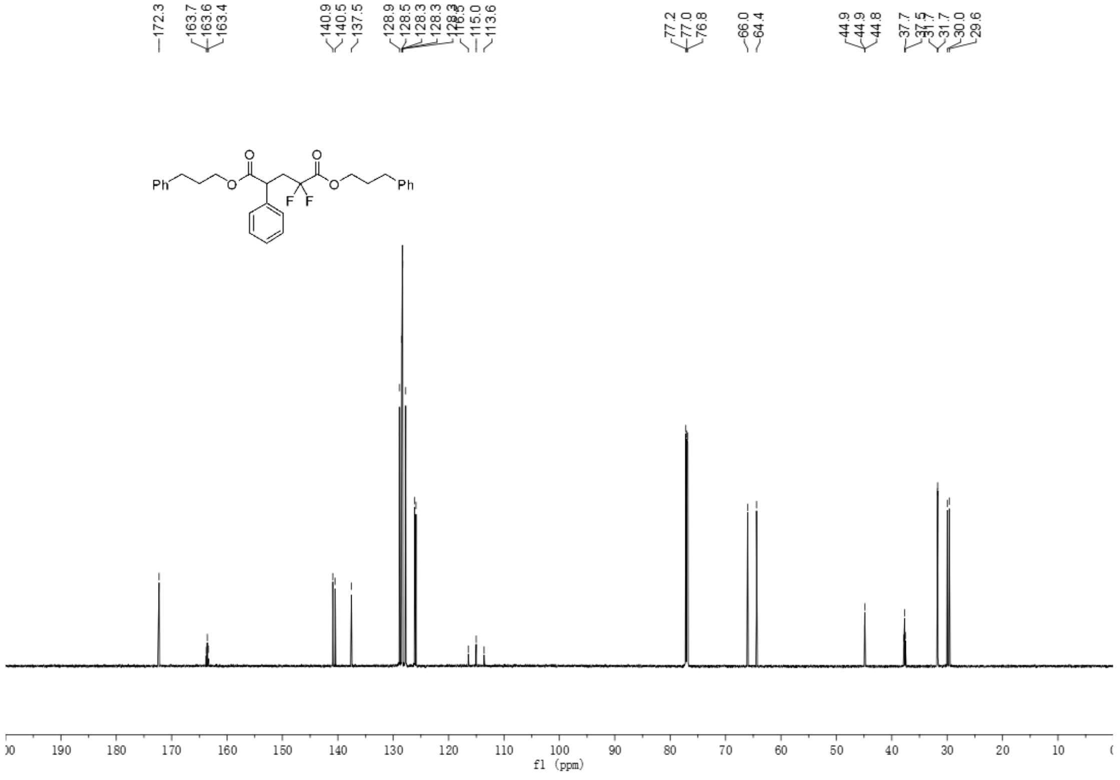

^{19}F NMR (56 MHz, $CDCl_3$) 3ag

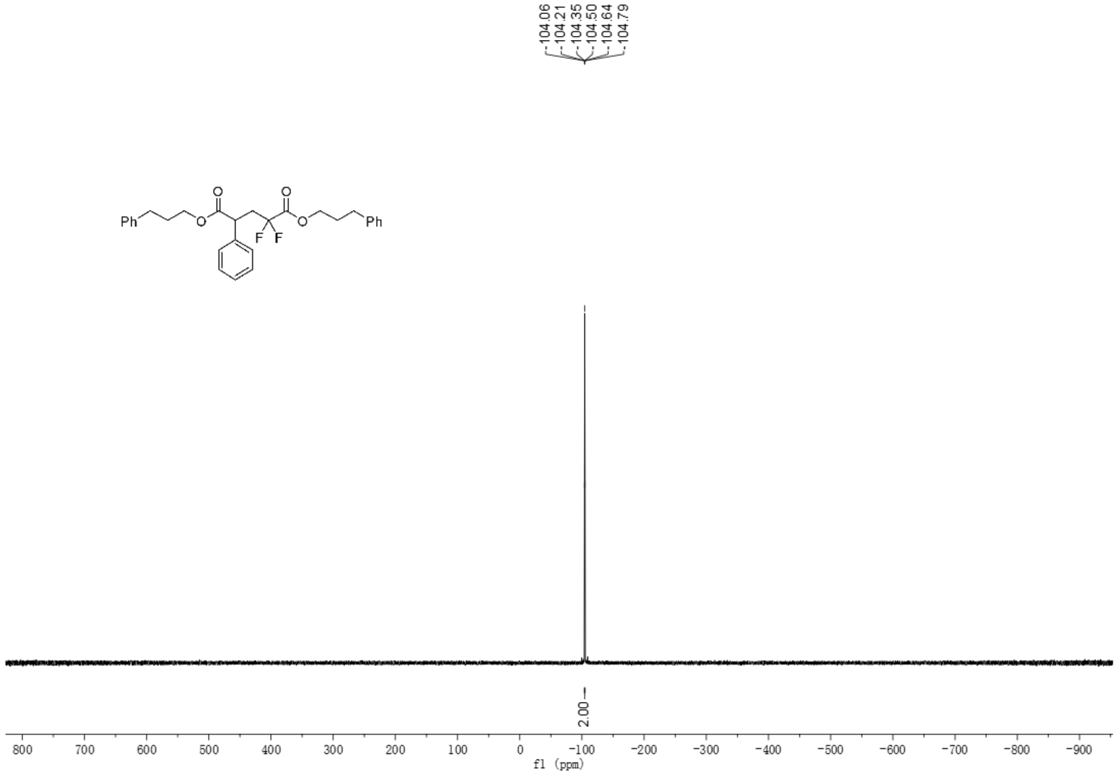

^{1}H NMR 400 MHz, DMSO

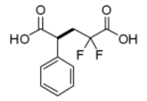

4

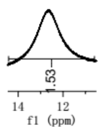

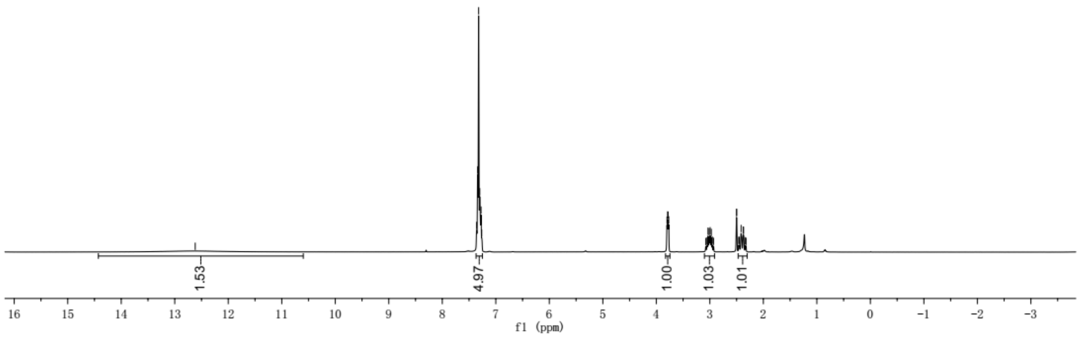

^{13}C NMR 101 MHz, DMSO

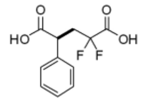

4

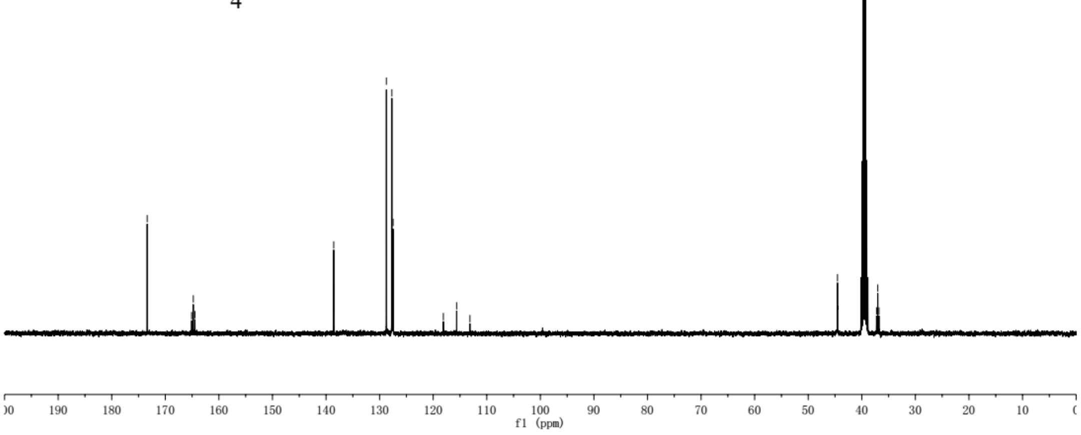

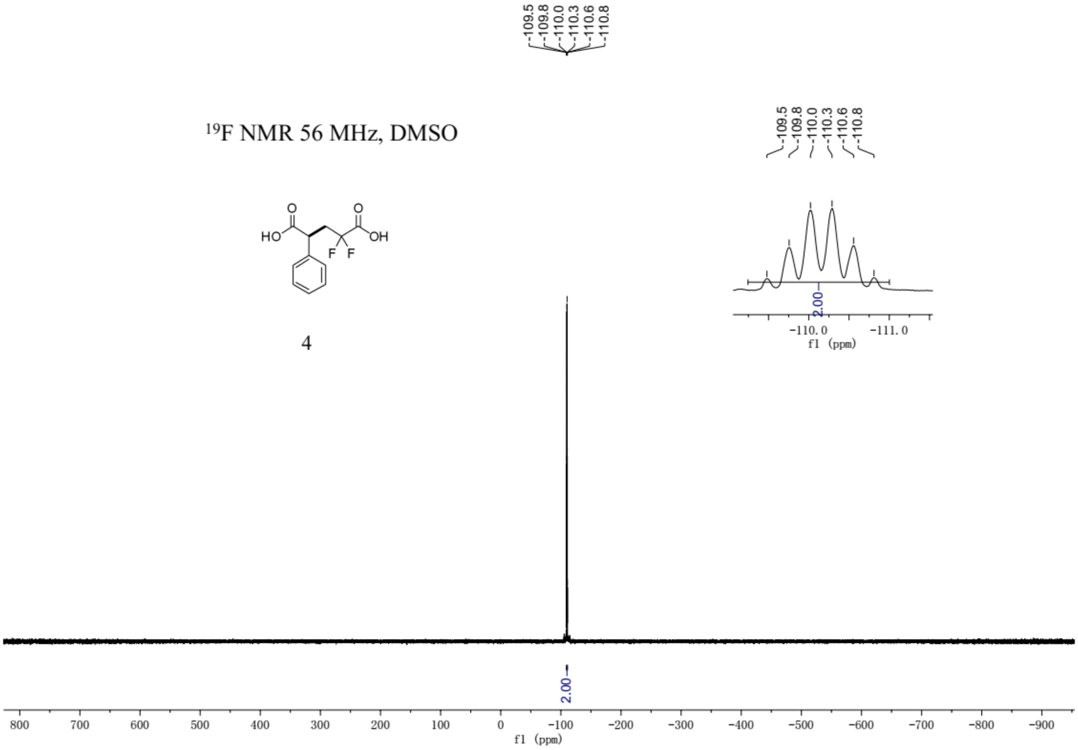

-109.5
-109.8
-110.0
-110.3
-110.6
-110.8
^{19}F NMR 56 MHz, DMSO
4
2.00
-110.0
-111.0
f1 (ppm)
800 700 600 500 400 300 200 100 0 -100 -200 -300 -400 -500 -600 -700 -800 -900

^{1}H NMR 700 MHz, $CDCl_3$

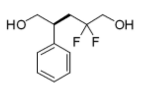

5

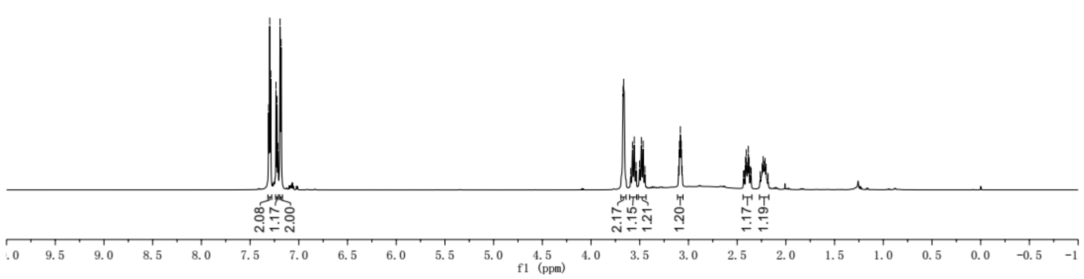

^{13}C NMR 176 MHz, $CDCl_3$

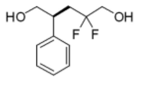

5

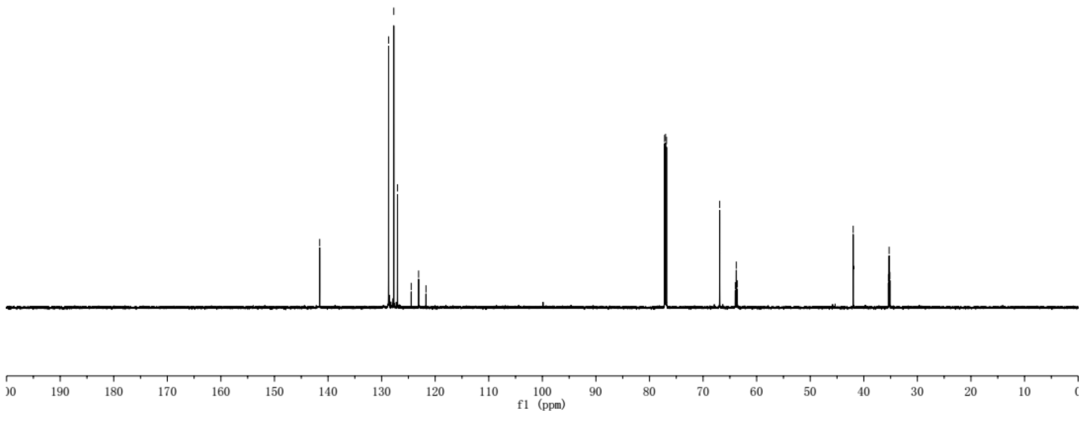

^{19}F NMR 56 MHz, $CDCl_3$

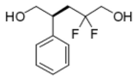

5

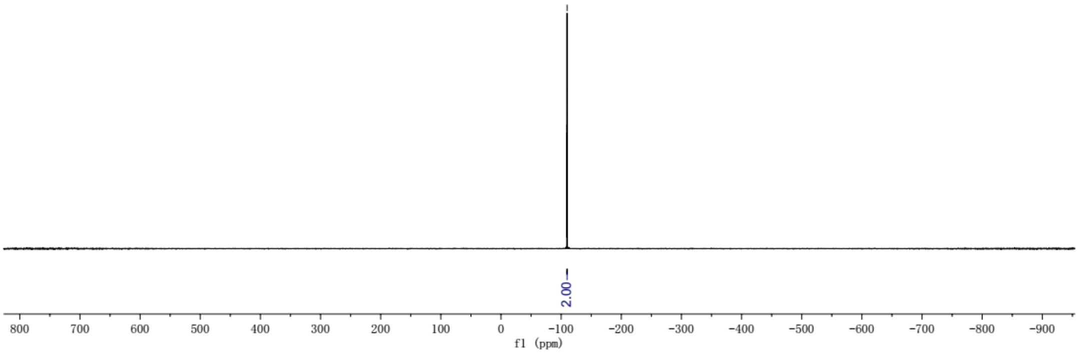

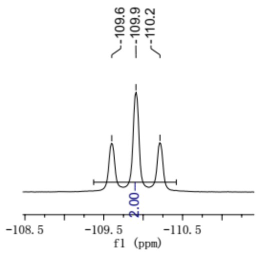

^{1}H NMR (700 MHz, CDCl$_3$) 8

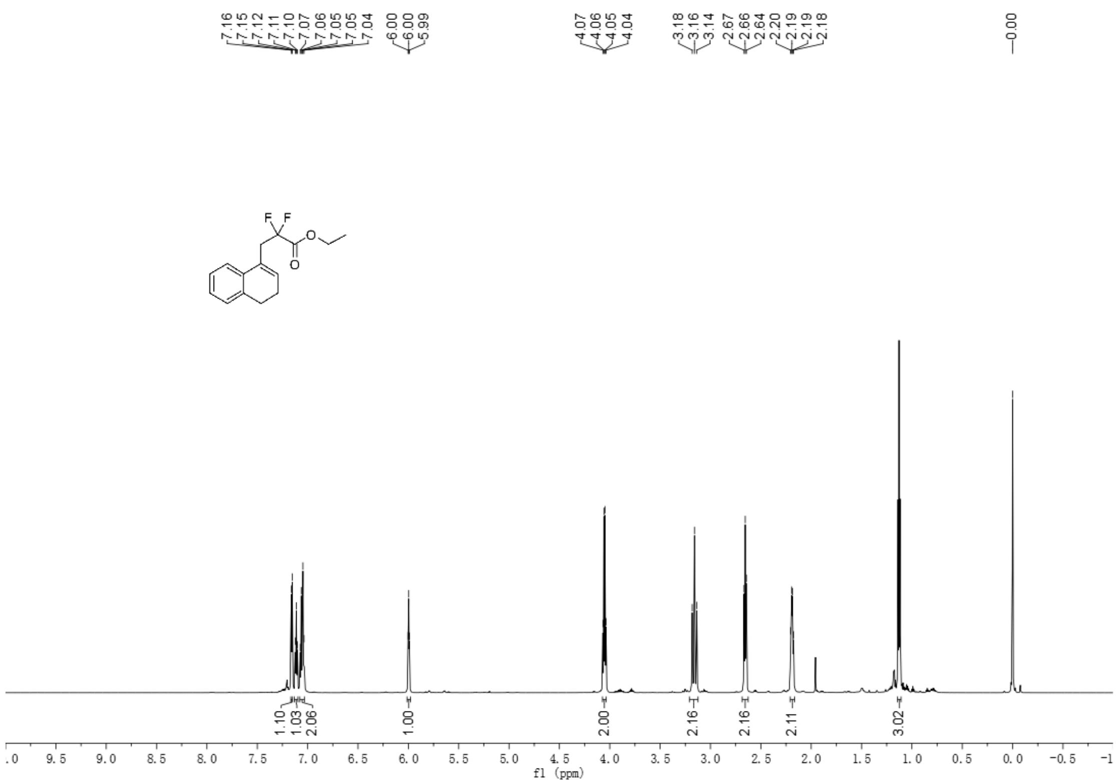

^{13}C NMR (176 MHz, CDCl$_3$) 8

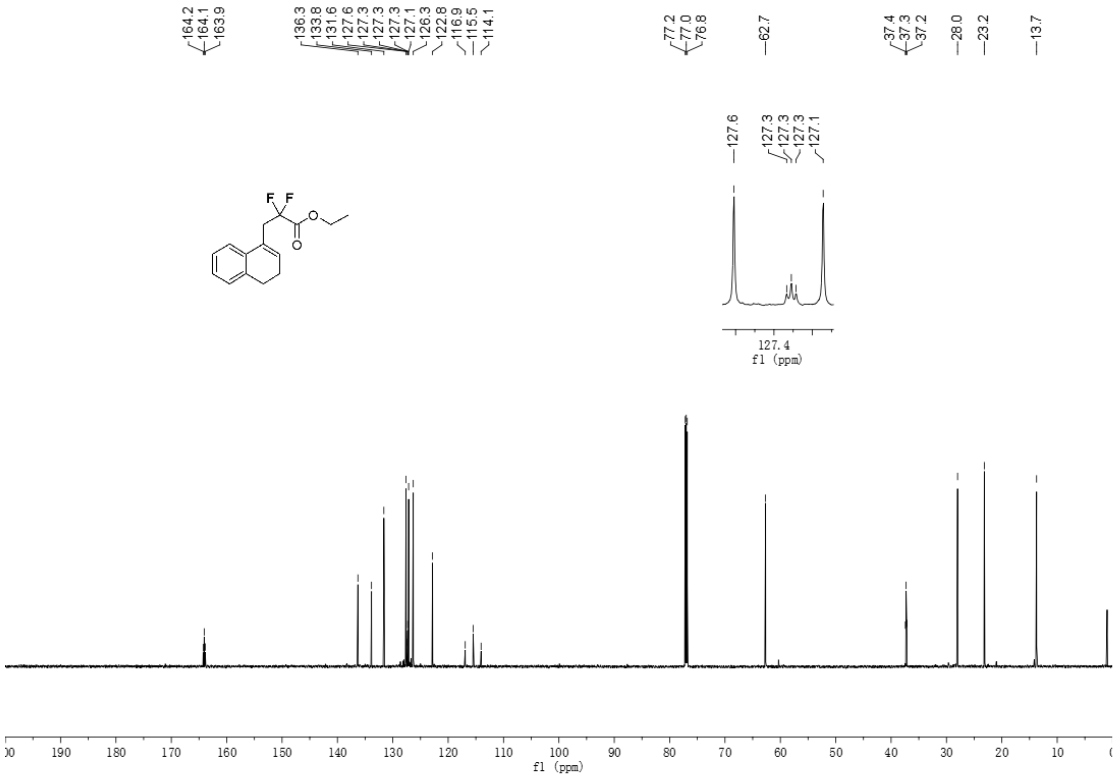

^{19}F NMR (56 MHz, CDCl$_3$) 8

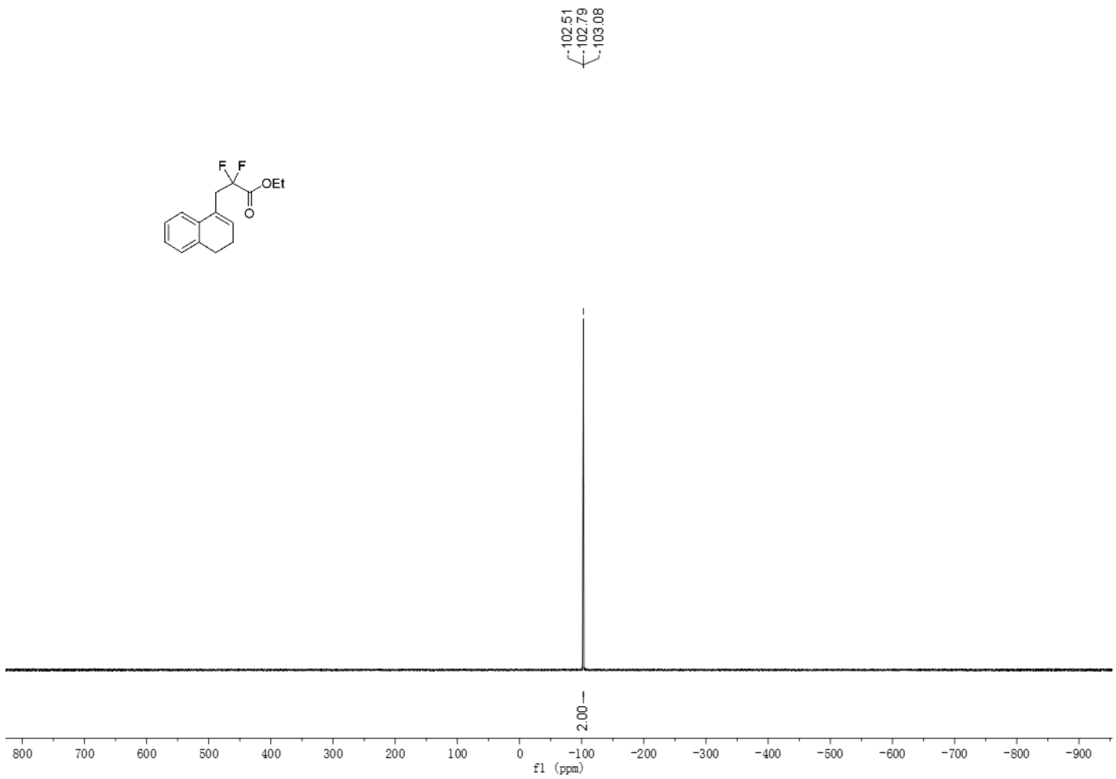

Supplement: SC-013-D2SC02665A-s001 [file SC-013-D2SC02665A-s001.pdf]
